# Neugeborenen-Screening aus Trockenblut (NBS) in Deutschland: Status quo und Vorstellung eines Konzeptes zur Weiterentwicklung

**DOI:** 10.1007/s00103-023-03771-8

**Published:** 2023-10-10

**Authors:** Uta Nennstiel, Birgit Odenwald, Veronika Throner, Oliver Blankenstein, Andreas Vieth, Rudolf Ratzel, Michaela Coenen, Inken Brockow

**Affiliations:** 1 Sachgebiet GP1: Gesundheitsberichterstattung, Epidemiologie, Sozialmedizin, Bayerisches Landesamt für Gesundheit und Lebensmittelsicherheit (LGL), Oberschleißheim, Bayern Deutschland; 2 Lehrstuhl für Public Health und Versorgungsforschung, Institut für Medizinische Informationsverarbeitung, Biometrie und Epidemiologie (IBE), Medizinische Fakultät, Ludwig-Maximilians-Universität München, München, Bayern Deutschland; 3 Institut für Experimentelle Pädiatrische Endokrinologie, Charité-Universitätsmedizin Berlin, Berlin, Deutschland; 4 Philosophisches Seminar, Universität Münster, Münster, NRW Deutschland; 5 Rechtsanwaltskanzlei Ratzel Rechtsanwälte, München, Bayern Deutschland

**Keywords:** Tracking, Screening-Programm, Trockenblut, Qualitätssicherung, Public Health Action Cycle, Tracking, Neonatal screening, Dried blood spots, Screening programme, Quality management

## Abstract

Das Neugeborenen-Screening aus Trockenblut (Newborn Blood Spot Screening, NBS) ist eine seit vielen Jahren etablierte hocheffektive Maßnahme der Sekundärprävention. Aufgrund der Entwicklung neuer diagnostischer und therapeutischer Möglichkeiten für seltene angeborene Krankheiten ist davon auszugehen, dass in Zukunft weitere Zielkrankheiten in das NBS aufgenommen werden. Vor diesem Hintergrund wurde im Auftrag des GKV-Spitzenverbandes ein Konzept für die Weiterentwicklung des NBS entwickelt. Als Grundlage hierfür dienten eine systematische Literaturrecherche und die Erhebung des Status quo in Deutschland anhand von quantitativen und qualitativen Methoden.

Entscheidend für den Erfolg des NBS ist, dass möglichst alle von einer Zielkrankheit betroffenen Neugeborenen frühzeitig diagnostiziert und behandelt werden und der bei jedem Screening zu erwartende Schaden (z. B. durch falsch-positive Befunde) möglichst gering gehalten wird. Hierfür ist die Organisation des Screenings im Sinne eines integrierten Programms durch eine zentrale Koordination mit standardisierten Strukturen, kontinuierlichem Qualitätsmanagement und einer datenschutzkonformen Digitalisierung erforderlich.

Obwohl das NBS in Deutschland insgesamt erfolgreich umgesetzt wird, zeigen sich im Rahmen des hier vorgestellten Forschungsprojekts auch Schwächen und Handlungsbedarf. Erarbeitete Vorschläge und Empfehlungen wurden in einem Konzeptpapier festgehalten, das Ansätze für eine dem aktuellen Forschungsstand entsprechende Weiterentwicklung des NBS aufzeigt und dabei sich ändernde Anforderungen an die Infrastruktur und Prozesse im Gesundheitssystem berücksichtigt. In diesem Übersichtsartikel werden Herausforderungen, aktueller Stand und Lösungsansätze zu zentralen Themenfeldern des Konzeptpapiers zusammenfassend dargestellt.

**Zusatzmaterial online:**

Zusätzliche Informationen sind in der Online-Version dieses Artikels (10.1007/s00103-023-03771-8) enthalten.

## Abstract (en)

Newborn screening from dried blood spots (NBS) is a highly effective secondary prevention measure that has been established for many years. Against the background of the inclusion of “new diseases” that meet the screening criteria, a concept for the further advancement of NBS was developed on behalf of the GKV-Spitzenverband. This was based on a systematic literature review and a survey of the status quo of NBS in Germany using quantitative and qualitative methods.

It is essential for the success of NBS that all newborns affected by a target disease are diagnosed and treated at an early stage and that the harm to be expected with each screening (e.g., due to false positive findings) is kept as low as possible. This requires the organisation of screening in the sense of an integrated programme through central coordination with standardised structures, continuous quality management and digitalisation in line with data protection requirements.

Although in general NBS is being implemented successfully in Germany, the research project presented here also reveals weaknesses and a need for action. Proposals and recommendations were compiled in a concept paper, which shows approaches for further development of NBS in line with the current state of research in consideration of changing demands on the infrastructure and processes in the health system. This review article summarises the challenges, current status and possible solutions for the central topics of the concept paper.

## Hintergrund

Das Neugeborenen-Screening aus Trockenblut (Newborn Blood Spot Screening, NBS) ist eine sehr effektive Maßnahme der Sekundärprävention [1–8], die allen Neugeborenen in Deutschland in den ersten 3 Lebenstagen angeboten werden muss [9, 10]. Das NBS wird durch den Gemeinsamen Bundesausschuss (G-BA) in der Kinder-Richtlinie [9] geregelt und unterliegt dem Gendiagnostikgesetz (GenDG; [11]). Untersucht wird das Blut auf derzeit 17 Zielkrankheiten aus 6 unterschiedlichen pädiatrischen Spezialgebieten [9, 10]. Alle diese Krankheiten sind (sehr) selten und müssen frühzeitig diagnostiziert und behandelt werden, um schwere gesundheitliche Schäden wie lebenslange Behinderung oder Tod zu vermeiden [2, 12–14]. Aus individualmedizinischer Sicht ist dieses Screening für die Betroffenen und deren Familien ein großartiger medizinischer Fortschritt. Um die wenigen betroffenen Kinder zu finden (ca. 1 von 750 Neugeborenen), muss allerdings die zu über 99,8 % nicht betroffene „gesunde“ Neugeborenen-Population mituntersucht werden. Wie jedes Screening kann das NBS neben dem großen Nutzen für die betroffene Person auch Schaden verursachen [12, 15–17]. Beispielsweise führen falsch-positive (auffällige) Befunde zu einer Belastung des Gesundheitssystems und können eine kurz- oder langfristige psychosoziale Belastung der Familien auslösen [2, 4, 15, 18]. Als zentrales ethisches Leitprinzip für Screening-Programme gilt daher: „Der Gesamtnutzen des Screenings soll den Schaden überwiegen“ [17, 19].

Aufgrund neuer therapeutischer und diagnostischer Möglichkeiten wird zunehmend die Aufnahme weiterer Zielkrankheiten in das NBS gefordert [2, 15, 20]. Mit der Aufnahme jeder neuen Krankheit und sich ändernder gesellschaftlicher Infrastruktur und Prozesse im Gesundheitswesen wachsen auch die Anforderungen an das NBS [2, 21]. Vor diesem Hintergrund vergab der GKV-Spitzenverband den Auftrag für ein Forschungsprojekt zur Erarbeitung von Vorschlägen für die Weiterentwicklung der Strukturen und Prozesse des in Deutschland etablierten NBS an die Autor*innen, um auch bei Einführung neuer Zielkrankheiten ein hohes Qualitätsniveau des gesamten Screening-Prozesses zu gewährleisten und dieses nachhaltig zu sichern. Die Strukturen und Prozesse des NBS sollten auf wissenschaftliche Aktualität, Effektivität, praktische Umsetzbarkeit und Weiterentwicklungsbedarf mithilfe quantitativer und qualitativer Verfahren sowie einer systematischen Literaturrecherche überprüft werden. Die Bewertung möglicher neuer Zielkrankheiten hinsichtlich der Evidenz zur Einführung eines Neugeborenen-Screenings erfolgt in Deutschland unter Berücksichtigung der bekannten Screening-Kriterien [17, 19, 22–24] durch das Institut für Qualität und Wirtschaftlichkeit im Gesundheitswesen (IQWiG; [2]) und war daher nicht Inhalt dieses Konzeptes. Erarbeitete Vorschläge und Empfehlungen zur Weiterentwicklung des NBS wurden in einem Konzeptpapier (s. Onlinematerial zu diesem Artikel) festgehalten. Die zentralen Aspekte des Konzeptes werden in der vorliegenden Publikation dargestellt.

## Methode und Datengrundlage

### Datengrundlage

#### Analyse der Qualitätsberichte der DGNS und Befragung in Geburtskliniken in Deutschland.

Über 10 Mio. durch die Deutsche Gesellschaft für Neugeborenen-Screening (DGNS) für die Jahre 2006 bis 2019 erhobene Datensätze [25] wurden im Längs- und Querschnitt vertieft analysiert. Vertreter*innen der insgesamt 650 Geburtskliniken in Deutschland wurden mit einem Online-Tool (LimeSurvey.org: Open Source Survey Tool) anonym zur Aufklärung der Eltern über das NBS, zu dessen Durchführung und zur Mitteilung auffälliger Befunde an die Eltern befragt. Die Teilnahmerate lag bei 30 % mit einer repräsentativen Verteilung der teilnehmenden Geburtskliniken.

#### Interviews und moderierte Diskussionsrunden.

Je 5 Vertreter*innen von Geburtskliniken unterschiedlicher Versorgungslevel, pädiatrischen Praxen und Hebammen aus 10 Bundesländern sowie Vertreter*innen aus jedem Screening-Labor wurden in semistrukturierten Einzelinterviews befragt. Zusätzlich fanden je eine moderierte Fokusgruppendiskussion mit 16 Vertreter*innen aus allen 11 Screening-Laboren in Deutschland und mit 10 Expert*innen aus 7 verschiedenen pädiatrischen Spezialgebieten und 6 Bundesländern sowie 1 Experten aus Österreich statt.

Die Einzelinterviews und Gruppendiskussionen wurden, nach vorheriger Zustimmung der Interviewpartner*innen, unter Anwendung von Interviewleitfäden virtuell über ein gesichertes Videokonferenz-Tool (Zoom) durchgeführt und digital aufgezeichnet. Die Interviews und die Gruppendiskussionen wurden wörtlich transkribiert und die Transkripte inhaltsanalytisch sowohl induktiv als auch deduktiv nach der Methode von Philipp Mayring [26] ausgewertet. Alle Analysen wurden mit SPSS Statistics for Windows Version 25.0. (IBM Corp. Armonk, NY, USA) durchgeführt.

#### Systematische Literaturrecherche.

Gleichzeitig wurde eine systematische Literaturrecherche zum NBS sowie zu Best-Practice-Modellen in anderen Ländern durchgeführt. Aus über 15.000 gefundenen und gesichteten Titeln wurden letztendlich 292 Zeitschriftenartikel und 199 Internetdokumente als relevant identifiziert und für die Konzeptentwicklung ausgewertet. Bei der Suche nach Best-Practice-Modellen des NBS wurden grundlegende Informationen für alle Staaten innerhalb der Organisation für wirtschaftliche Zusammenarbeit und Entwicklung (OECD) aus Publikationen und Internetdokumenten gesammelt sowie für ausgewählte Programme durch persönliche Kontaktaufnahme ergänzt. Methodik und Ergebnisse der Literaturrecherche zu Prozessen und Strukturen des NBS sind ausführlich in einer eigenen Publikation dargestellt [27].

### Konzeptentwicklung

**Figure 1:**
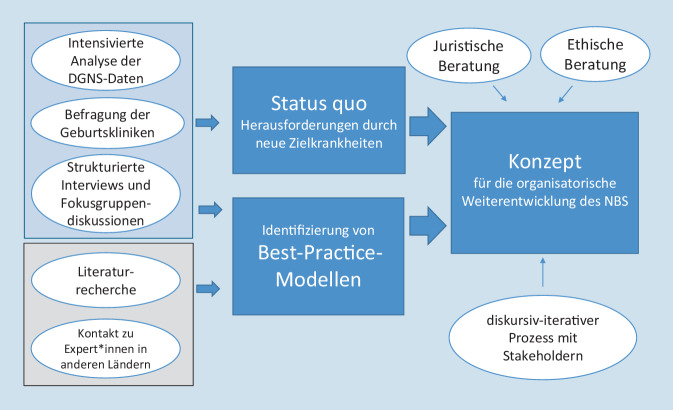

Anhand der strukturierten Ergebnisse aus der Datenanalyse, den Interviews und der Literaturrecherche wurden die Schwerpunkte für die anschließend ausgearbeiteten Vorschläge identifiziert und in einem Konzeptpapier festgehalten (s. Onlinematerial). Optionen für die organisatorische Weiterentwicklung des NBS wurden in einem diskursiv-iterativen Prozess der partizipativen Modellbildung und -anpassung unter Einbindung von juristischer und ethischer Expertise erarbeitet (Abb. 1). In einem letzten Schritt diskutierten Mitglieder der Screening-Kommission der Deutschen Gesellschaft für Kinder- und Jugendmedizin (DGKJ) das Konzept. Die Konzeptvorschläge gehen grundsätzlich von geltendem Recht aus, machen jedoch hinsichtlich der organisatorischen Abläufe Vorschläge zu einer strukturellen Verbesserung, die teilweise eine Anpassung der einschlägigen Normen erfordern.

## Status quo und Weiterentwicklungsbedarf für das NBS in Deutschland

### Strukturen des NBS in Deutschland

**Figure 2:**
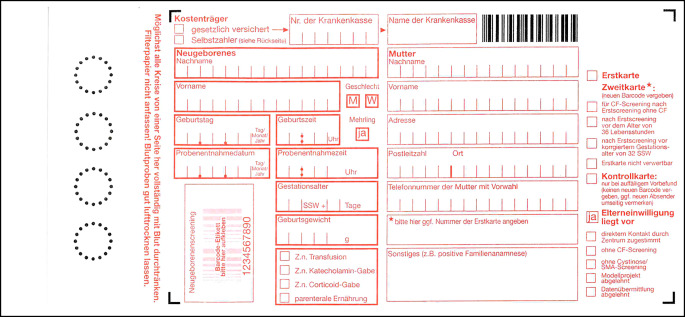

Das NBS in Deutschland ist in der Kinder-Richtlinie [9] folgendermaßen geregelt: Die Verantwortung für die Durchführung des Screenings liegt bei der Person, die die Geburt verantwortlich geleitet hat (Einsender). Vor der Blutentnahme ist eine ärztliche Aufklärung mit schriftlicher Einwilligung der Eltern notwendig. Die Blutprobe wird dann zwischen 36 und 72 Lebensstunden meist in der Geburtsklinik abgenommen und auf eine Testkarte getropft (Abb. 2).

**Figure 3:**
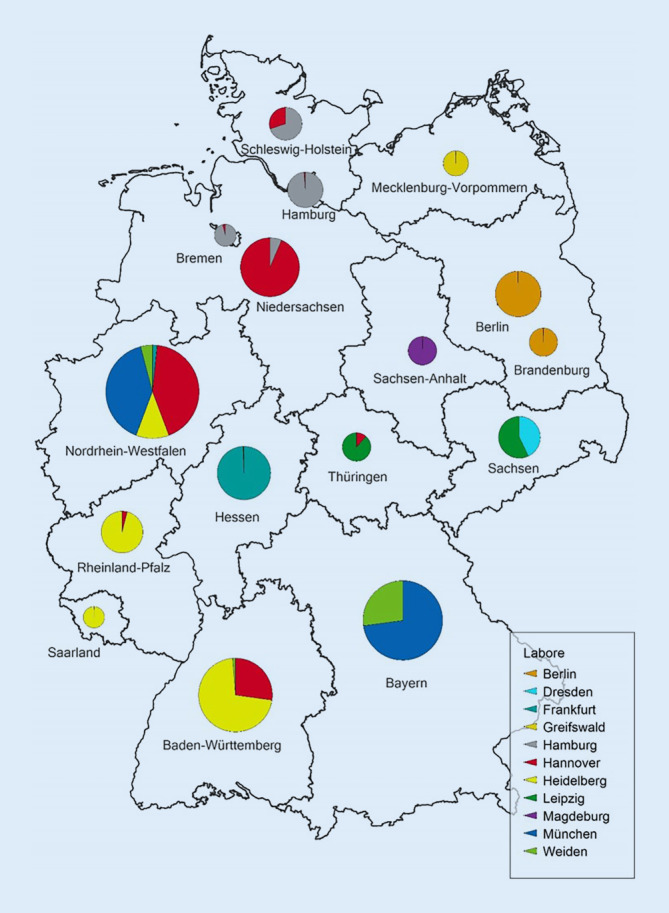

Zur Analytik werden diese Testkarten in eines von 11 für das Screening in Deutschland akkreditierten Labore geschickt, die teils bundeslandübergreifend arbeiten (Abb. 3; [25]) und nur in wenigen Ländern, wie Bayern, Berlin/Brandenburg und Sachsen-Anhalt bei der Qualitätssicherung des Screening-Prozesses durch ein zentrales Screening-Zentrum unterstützt werden. Das Labor teilt den Screening-Befund dem Einsender mit. Dieser muss im Falle eines auffälligen Befundes umgehend die Eltern informieren und ihnen, wenn eine weiterführende Diagnostik (Konfirmationsdiagnostik) erforderlich ist, die Adressen und Kontaktdaten der spezialisierten Zentren für die jeweilige Krankheit nennen. Dort sollen die Eltern dann einen baldigen Termin vereinbaren.

Die Labore haben regelmäßige Qualitätsberichte abzugeben, die jährlich gemeinsam für alle deutschen Screening-Labore als Nationaler Screeningreport von der DGNS ehrenamtlich erstellt und publiziert werden [25].

### Vollständigkeit des NBS

International besteht Konsens, dass eine systematische Erfassung der Zielpopulation Grundbedingung für eine hohe Effektivität des NBS ist. Um sicherzustellen, dass jedes Neugeborene erreicht wird, müssen durch geeignete Trackingverfahren der Eingang der Testkarten im Labor überprüft und die Eltern ggf. auf das fehlende Screening aufmerksam gemacht werden [2, 6, 7, 17, 28].

#### Situation in Deutschland.

In Deutschland soll durch die Festschreibung der Verantwortlichkeiten die systematische Erfassung der Zielpopulation gesichert werden und auch die DGNS-Datenanalyse lässt mit einer Screening-Rate von über 99 % auf eine hohe Vollständigkeit schließen. Allerdings ist dieser Wert möglicherweise aufgrund von fälschlicherweise als Erstscreening eingeordneten Folgeuntersuchungen überschätzt. Auf Individualebene wird nicht flächendeckend überprüft, ob das Blut wirklich abgenommen wurde und die Testkarte im Labor eingegangen ist. Aus Bundesländern, die einen Abgleich von geborenen mit gescreenten Kindern durchführen, ist bekannt, dass ca. 1 von 1300 Testkarten verloren gegangen ist und bei weiteren Kindern das NBS z. B. bei Verlegung vergessen wurde.

### Qualitätssicherung im Screening-Labor

Wichtige Qualitätsparameter in einem Screening-Labor sind valide und reliable Analysen der festgelegten Screening-Parameter sowie eine valide Befundung der Testergebnisse als unauffällig, grenzwertig oder auffällig [1, 4, 17, 29]. Grenzwerte (Cut-offs) müssen für jeden Parameter so festgelegt werden, dass möglichst alle Betroffenen erkannt werden können (hohe Sensitivität; [4, 7]). Trotzdem soll gleichzeitig der Anteil falsch-positiver Befunde möglichst geringgehalten werden (hohe Spezifität; [7, 29, 30]). Erreicht werden kann dies im NBS bei einigen Krankheiten durch multiple, biochemische oder molekulargenetische Parameter, 2‑ oder auch 3‑stufige Testverfahren [4, 20, 31] und regelmäßige Überprüfung und ggf. Anpassung der Grenzwerte. Mit gleichem Ziel werden in den letzten Jahren weltweit zunehmend auch postanalytische multivariate digitale Interpretationswerkzeuge genutzt [20, 30, 32, 33].

#### Situation in Deutschland.

**Figure 4:**
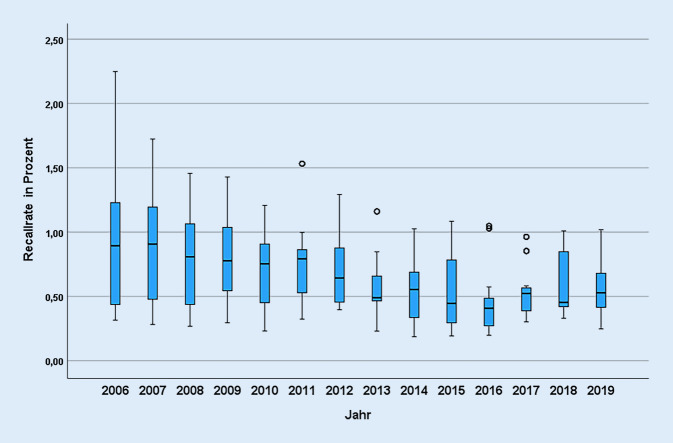

Die Kinder-Richtlinie [9] legt die Voraussetzungen für die Zulassung als Screening-Labor fest. Die seit 2005 in der Richtlinie festgelegten Analysemethoden wurden seitdem nicht aktualisiert. Vorgaben zu Qualitätsparametern bei der Befundung der Testergebnisse fehlen bisher. Die Analyse der DGNS-Daten zeigt erhebliche Unterschiede zwischen den Laboren beispielsweise bei der Rate an auffälligen Befunden (Recall-Raten; Abb. 4; [7]), so dass aufgrund der Daten sowie auch in den Interviews und Diskussionen mit den Vertreter*innen der Labore Handlungsbedarf hinsichtlich einer weitergehenden Qualitätssicherung gesehen wird.

### Befundmitteilung

Ein auffälliger Screening-Befund bedeutet noch keine Diagnose, sondern zunächst nur einen Krankheitsverdacht, der durch geeignete Methoden zeitnah bestätigt oder ausgeschlossen werden muss [15, 17, 28]. Die Mitteilung eines auffälligen Befundes an die Eltern stellt insofern eine Herausforderung dar, als einerseits die Dringlichkeit der weiteren Untersuchungen vermittelt und andererseits dabei unnötige Verunsicherung möglichst vermieden werden soll [2, 34, 35]. Die Bedeutung der kompetenten Befundmitteilung durch eine sowohl über die Prozesse als auch über die Krankheit gut informierte Person wird in der Literatur betont [34–36] und sowohl in der Befragung der Geburtskliniken als auch bei den Fokusgruppendiskussionen gefordert. Als optimal gilt die Befundmitteilung an die Eltern durch Expert*innen, die dann auch die Abklärung durchführen [34, 35].

#### Situation in Deutschland.

In Deutschland muss das Labor dem Einsender (meist aus der Geburtsklinik) einen auffälligen Befund, entsprechend den Regelungen des GenDG, übermitteln und nur dieser darf den Eltern den Befund mitteilen [9, 11]. Die zugrunde liegende Regelung des § 11 GenDG [11] ist aus der Sicht der Gendiagnostikkommission für das NBS ungeeignet [37]. Sie wird in der Online-Befragung, den Interviews und Diskussionen heftig kritisiert, da von dem Einsender die notwendige Kompetenz bei der Vielzahl der sehr seltenen Krankheiten nicht erwartet werden kann. Auch geht häufig wertvolle Zeit durch die komplexen Abläufe verloren und die Familien sind aufgrund der unzureichenden Information sehr beunruhigt [36, 38].

### Abklärung aller auffälligen Screening-Befunde (Tracking)

Die Sicherstellung einer vollständigen zeitnahen Abklärung aller wiederholungsbedürftigen oder auffälligen Screening-Befunde bis zum Ausschluss oder der Bestätigung einer Zielkrankheit (Tracking) ist entscheidend für den Erfolg eines NBS-Programms. Ohne ein systematisches Tracking besteht das Risiko, dass aufgrund vermeidbarer Fehler, Nachlässigkeiten oder Fehleinschätzungen betroffene Kinder trotz NBS zu spät entdeckt oder ganz übersehen werden, was in einer Tragödie für diese Kinder und deren Familien enden kann [2, 6, 28].

#### Situation in Deutschland.

In der Kinder-Richtlinie fehlen Vorgaben zu einem Tracking. Die Daten der DGNS zeigen, dass in Deutschland bei ca. 20 % der wiederholungsbedürftigen Testkarten und für ca. 10 % der Kinder mit hochgradigem Krankheitsverdacht unklar ist, ob eine endgültige Abklärung erfolgt ist („loss to follow-up“; [7, 8]). Diese Rate liegt in einigen Laboren bei über 30 %, während in anderen die Ergebnisse der Konfirmationsdiagnostik fast vollständig vorliegen [7]. Als besonders effektiv erweist sich hier die Kooperation mit einem Screening-Zentrum [2, 6, 39].

### Konfirmationsdiagnostik

Essentiell für den Erfolg des NBS sind sowohl eine zeit- und leitliniengerechte Diagnostik zur Abklärung eines auffälligen Screening-Befundes (Konfirmationsdiagnostik) und ggf. ein frühzeitiger Therapiebeginn als auch eine von Anfang an fachlich und kommunikativ kompetente Begleitung der Eltern [1, 15, 20, 28, 34, 40]. Da alle Zielkrankheiten des NBS zu den (sehr) seltenen Krankheiten gehören, bei denen die Patient*innen auf eine hochkompetente, spezialisierte Diagnostik und medizinische Versorgung angewiesen sind, wird diese am besten in spezialisierten Behandlungszentren gewährleistet [29, 41, 42]. In den Diskussionen mit Vertreter*innen der Labore und Zentren bestand, wie auch in der Literatur, Einigkeit, dass die Konfirmationsdiagnostik ausschließlich in kompetenten Zentren erfolgen sollte.

#### Situation in Deutschland.

In Deutschland fehlen Regelungen für eine Zulassung von Zentren oder Kliniken zur qualifizierten Konfirmationsdiagnostik. Das bedeutet, dass jede Kinderklinik und jede kinderärztliche Praxis die Abklärung bei Verdacht auf eine der sehr seltenen Krankheiten vornehmen dürfen, unabhängig von der dort vorhandenen Kompetenz.

### Digitalisierung

In der Literatur, den Interviews und Diskussionen bestand Einigkeit, dass digitale Übermittlungs- oder Abfragewege den Screening-Prozess vereinfachen, beschleunigen und absichern können. Dies gilt z. B. für die Erfassung der auf der Testkarte manuell eingetragenen Angaben zum Kind [33], die Nachverfolgung des Versands der Testkarte bis zum Eingang im Labor, die Übermittlung der Screening-Befunde und der Ergebnisse der Konfirmationsdiagnostik sowie die Langzeitbeobachtung bzw. Patientenregister [15, 33, 43]. Eine zentrale Datenplattform (Datenbank) kann darüber hinaus dem Monitoring und der Qualitätssicherung sowie einer Evaluation des NBS [34] dienen.

#### Situation in Deutschland.

In Deutschland sind die Prozessabläufe sowie der Datenfluss im Rahmen des NBS nicht mehr zeitgemäß und dringend reformbedürftig. So werden Daten zunächst manuell auf der Testkarte erfasst (Abb. 2) und anschließend im Labor in die dortige Datenbank eingegeben. Die Screening-Ergebnisse werden vom Labor schriftlich an den Einsender übermittelt, auffällige Befunde häufig telefonisch und zusätzlich per Fax. Für die ggf. rasch notwendige Konfirmationsdiagnostik soll der Einsender den Eltern und diese dem Behandlungszentrum den Screening-Befund übermitteln. Insgesamt sind die Abläufe nicht ausreichend standardisiert, zu aufwändig, fehleranfällig, datenschutzrechtlich problematisch und können bei Befunden mit hochgradigem Krankheitsverdacht zu einem kritischen Zeitverlust führen.

### Dokumentation und Evaluation

Dokumentation und Evaluation von Screening-Programmen einschließlich der Ergebnisse der Konfirmationsdiagnostik, der Langzeitbeobachtung und der gesundheitsökonomischen Aspekte [1, 15, 24] gelten als essentiell für die kontinuierliche Qualitätsentwicklung der Programme [1, 15, 20, 28, 34]. Zunächst sollten Zielparameter für die Qualitätsbewertung definiert werden [15, 28, 40], deren Zielerreichung regelmäßig überprüft und in Qualitätsberichten dargestellt wird [17, 34].

#### Situation in Deutschland.

Die DGNS erstellt zwar wie oben beschrieben jährliche Qualitätsberichte [25]. Eine Ableitung und Umsetzung kontinuierlicher qualitätssichernder Maßnahmen auf deren Basis ist jedoch weder vorgesehen noch etabliert, obwohl hier seit Jahren in verschiedenen Bereichen erhebliche Qualitätsunterschiede zwischen den Laboren erkennbar sind (Beispiel Abb. 4). Auch die Rückmeldung des Ergebnisses der Konfirmationsdiagnostik ist nicht geregelt und kann daher nicht systematisch in die Qualitätsbewertung oder eine Evaluation eingehen.

### Neugeborenen-Screening als Public-Health-Programm

Der wichtigste Nutzen des NBS ist der direkte gesundheitliche Benefit für Kinder mit einer der Zielkrankheiten [2, 12–14, 21]. Dabei gilt es, den Nutzen für diese Kinder zu maximieren und möglichen Schaden auch für die gesunde Population zu minimieren, indem das NBS als ein Public-Health-Programm und nicht nur als „Screening-Test“ organisiert wird [2, 4, 13, 17, 44–47]. Das Screening-Programm sollte von der Erfassung der Zielpopulation über die Aufklärung bis zur Diagnosestellung optimierte standardisierte Abläufe sowie ein permanentes Qualitätsmanagement im Sinne eines Public Health Action Cycle [48] vorsehen. Dies erfordert sowohl eine Qualitätssicherung der einzelnen Programmelemente und -prozesse [4, 15, 17, 45–47] als auch die Evaluation des gesamten Programms, inklusive der Zielkrankheiten [15]. Aus nicht erreichten Zielen müssen Konsequenzen folgen [2–4, 17, 45, 47, 48].

#### Situation in Deutschland.

Wie oben beschrieben sind in Deutschland die Grundsätze, die Durchführung und Laboranalytik der Screening-Tests sowie die Dokumentation nur weniger Qualitätsparameter in der Kinder-Richtlinie [9] geregelt. Das NBS wird bisher nicht als Public-Health-Programm gedacht, geschweige denn ist es als solches etabliert. Die notwendige Qualitätssicherung im Sinne eines Public Health Action Cycle [48] ist ebenfalls nicht vorgesehen.

## Diskussion und Konzeptvorschläge

In Deutschland wird das NBS mit einer hohen Vollständigkeit und der frühzeitigen Diagnosestellung bei mittlerweile ca. 1000 betroffenen Kindern im Jahr insgesamt sehr erfolgreich umgesetzt. Allerdings zeigen die Analysen, Interviews und Diskussionen im Rahmen dieses Projektes sowie die aktuelle Literatur [49] auch Schwächen und Handlungsbedarf. Defizite werden insbesondere in den Bereichen Prozesskoordination, Dokumentation, Evaluation und Qualitätssicherung mit fehlenden Mechanismen zur Optimierung und zur Minimierung möglicher Risiken des Screenings gesehen. Das Potenzial digitaler Datenaustauschsysteme zur Erleichterung, Beschleunigung und Sicherung des Screening-Prozesses wird bisher wenig genutzt. Die Umsetzung von Verbesserungen in diesen Bereichen wird z. B. erschwert durch den deutschen Föderalismus und ein mehrgliedriges Gesundheitssystem, die fehlende Koordination im Prozess, beim Datenaustausch und der Qualitätssicherung der 11 überregional voneinander unabhängigen Screening-Labore, das traditionelle Privileg der freien Arzt- und Klinikwahl, die geringe Digitalisierung im Gesundheitswesen, strenge Datenschutzbestimmungen und die für das NBS ungeeigneten Regelungen des GenDG.

Vor diesem Hintergrund wurden im Rahmen dieses Projektes an die Situation in Deutschland angepasste Empfehlungen (s. Infobox 1 und Onlinematerial) entwickelt, aus denen nachfolgend die wichtigsten zusammengefasst werden.

### Public-Health-Programm mit zentraler Koordination.

Die Koordination und Qualitätssicherung des NBS als Public-Health-Programm werden optimalerweise von einer zentralen, ausreichend finanzierten Koordinierungsstelle für ganz Deutschland übernommen. Denkbar sind jedoch auch regionale Koordinierungsstellen, die eng miteinander kooperieren. Entscheidend ist dabei, dass Organisation und Steuerung sowie Qualitätsmanagement für ganz Deutschland einheitlich geregelt und umgesetzt werden. Die Hauptaufgaben der Koordinierungsstelle sind in der Programmkoordination und Qualitätssicherung des NBS zu sehen. Darunter fallen die regelmäßige Analyse der Qualitätsberichte (DGNS-Report), die Identifizierung von Qualitätsmängeln und Anpassungs- bzw. Optimierungsbedarf sowie die Erarbeitung von Verbesserungsvorschlägen. Auch Öffentlichkeitsarbeit, die Bereitstellung von Schulungsmaterialien für alle am NBS-Prozess Beteiligten sowie auf den besten verfügbaren Erkenntnissen basierende Handlungsleitfäden (SOPs) für jeden Schritt des Screening-Programms würden hier erarbeitet und regelmäßig angepasst werden.

### Dokumentation und Evaluation.

**Figure 5:**
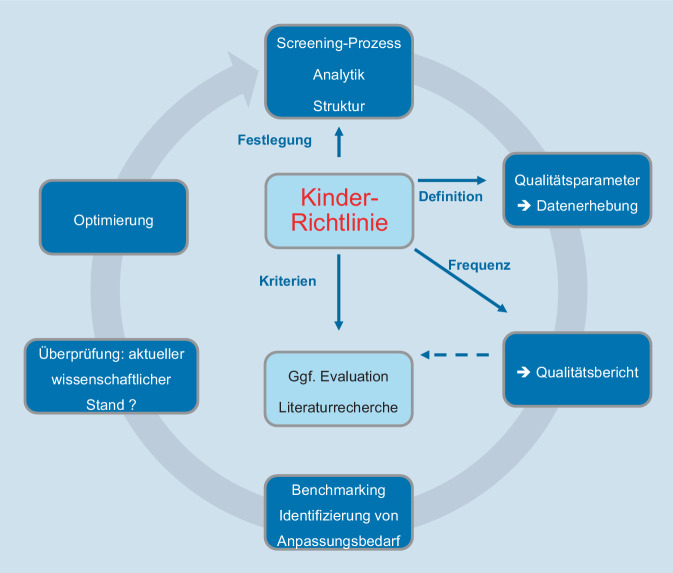

Für die Qualitätssicherung des NBS müssen in der Kinder-Richtlinie Zielparameter für die Qualitätsbewertung definiert werden, deren Zielerreichung regelmäßig überprüft werden soll. Auch eine standardisierte Dokumentation und Rückmeldung der Ergebnisse der Konfirmationsdiagnostik sollten etabliert und Patientenregister in Kooperation mit den Fachgesellschaften geführt werden. In den regelmäßigen Qualitätsberichten (z. B. DGNS-Report) aufgedeckte Schwächen und Qualitätsdefizite müssen im Sinne des Public Health Action Cycle (Abb. 5) innerhalb festgesetzter Fristen bearbeitet und daraus Konsequenzen, wie z. B. eine weitergehende Evaluation und Maßnahmen zur Qualitätsverbesserung in den betreffenden Programmschritten, gezogen werden. In Abhängigkeit vom Thema würden Vertreter*innen aus den Screening-Laboren, behandelnde Ärzt*innen (Expert*innen) und ggf. weitere Stakeholder partizipativ in den Diskurs zur Qualitätsverbesserung eingebunden.

### Digitalisierung.

Für die während des Screening-Prozesses laufend erforderliche Erfassung und Übermittlung von Daten wird die Etablierung einer zentralen bundesweiten digitalen Plattform auf einem sicheren Server mit rollenbasierter Zugriffskontrolle und einem datenschutzkonformen Schlüsselkonzept empfohlen. Diese Plattform enthält Daten aus dem gesamten Screening-Prozess. Jedes Neugeborene wird kurz nach der Geburt mit einer eineindeutigen Screening-ID als Pseudonym in die zentrale Plattform aufgenommen. Jede Untersuchung im Verlauf des Screening-Prozesses (erste Testkarte, Folgekarten, Konfirmationsdiagnostik) und auch später diagnostizierte Fälle nach unauffälligem Screening werden dieser Screening-ID zugeordnet. Der Screening-Datensatz kann über eine Schnittstelle in die krankheitsspezifischen Register der Fachgesellschaften eingespielt werden. Die oben genannten Qualitätsberichte können zu großen Teilen aus der Auswertung der anonymisierten Daten der Plattform erstellt werden, was die Datenqualität verbessern und den Arbeitsaufwand verringern dürfte.

### Tracking.

Das Tracking fehlender Screenings kann über die zentrale Datenplattform automatisiert erfolgen, indem den eineindeutigen Screening-IDs geborener Kinder die in den Laboren eingegangenen Testkarten zugeordnet und standardisierte Anschreiben an Eltern nicht gescreenter Kinder erzeugt werden. Die hierfür notwendige Zuordnung von Personendaten zu der Screening-ID kann über eine Vertrauensstelle erfolgen und ist für ca. 98 % der Neugeborenen nicht erforderlich, da bei ihnen ein unauffälliges Screening-Ergebnis vorliegt.

Das notwendige Tracking der Wiederholungs- und Kontrolluntersuchungen sowie der Konfirmationsdiagnostik könnte, mit entsprechender Finanzierung, über ein systematisches regionales Tracking durch Screening-Zentren oder Befundkoordinator*innen in den Laboren, ggf. mit Supervision durch eine zentrale Koordinierungsstelle, erfolgen. Für dieses Tracking kann das Labor auf der Datenplattform sehen, ob eine notwendige Kontrolluntersuchung durchgeführt wurde bzw. das Kind im Zentrum angekommen ist, und kann das Ergebnis der Konfirmationsdiagnostik zur Qualitätssicherung der Analytik und Befundung heranziehen.

### Qualitätssicherung im Screening-Labor.

Eine regelmäßige Überprüfung der in der Richtlinie vorgesehenen Analysemethoden für die einzelnen Zielkrankheiten ist aus wissenschaftlicher Sicht unerlässlich. Für die Analytik und die Prozesse im Labor müssen Qualitätsziele laborübergreifend festgelegt und regelmäßig evaluiert werden. Diese Aufgabe könnte auf Basis der jährlichen DGNS-Datenerhebung und einer kontinuierlichen Überprüfung des aktuellen wissenschaftlichen Standes durch die zentrale Koordinierungsstelle in Kooperation mit den Screening-Laboren übernommen werden. Eine regelmäßige Überprüfung und ggf. Korrektur der Grenzwerte und, soweit möglich, mehrstufige Testverfahren sollen zur Senkung der Recall-Rate eingesetzt werden. Durch die Teilnahme aller Screening-Labore an einem Verfahren mit postanalytischen multivariaten digitalen Interpretationswerkzeugen könnte die Effektivität des NBS in Deutschland weiter verbessert werden.

### Mitteilung auffälliger Screening-Befunde und Konfirmationsdiagnostik.

Eine deutschlandweite Vereinheitlichung des Prozessablaufs nach einem auffälligen Screening-Befund mit der Zuweisung zu einem qualifizierten Zentrum wird dringend empfohlen und sollte verbindlich geregelt werden. Diese Zentren sollten unter Federführung der jeweiligen Fachgesellschaft nach objektiv festgelegten und transparenten Qualitätskriterien ausgewählt und regelmäßig reevaluiert werden. Befunde mit hochgradigem Krankheitsverdacht sollten den Eltern in der Regel von Expert*innen aus diesen Zentren mitgeteilt werden. So können eine kompetente Information der Eltern über den abzuklärenden Krankheitsverdacht und eine rasche Diagnostik sichergestellt werden. Kontrollbedürftige Befunde, bei denen keine Abklärung in einem Zentrum erforderlich ist, könnten den Eltern direkt aus dem Labor von geschultem Personal mitgeteilt werden. Ein solches Konzept kann medizinische Nachteile für die Kinder vermeiden und das Kindeswohl schützen. Die Gendiagnostikkommission hält eine Befundmitteilung in Notsituationen, in denen Gefahr für das Leben und die körperliche Unversehrtheit der betroffenen Person besteht, durch andere Personen als den Einsender, die „in gleicher Weise kompetent“ sind, für zulässig. In ihrer Richtlinie zur Aufklärung [50] sieht sie eine solche Notsituation bei auffälligen Befunden im derzeitigen NBS als gegeben an. Standardisierte Schulungs- und Informationsmaterialien für Ärzt*innen und gut zugängliche Informationen für Eltern in der Situation „auffälliger Screening-Befund“ sollten auf einer zentralen Screening-Website z. B. durch die Koordinierungsstelle zur Verfügung gestellt werden.

### Weitere wichtige Aspekte.

In der vorliegenden Publikation werden ausgewählte zentrale Themenfelder des Konzeptes vorgestellt. Weitere wichtige Aspekte sind im ausführlichen Konzeptpapier nachlesbar, hierunter auch die Empfehlung einer pränatalen Eltern-Aufklärung, die den Autor*innen wichtig ist. Das Konzeptpapier (s. Onlinematerial) soll als Dokument verstanden werden, das für verschiedene Themenfelder die Herausforderungen und Weiterentwicklungsbedarfe in den Strukturen und Prozessen des NBS in Deutschland aufzeigt und Entscheidungsträger*innen in Politik und dem G‑BA Vorschläge zu Lösungsansätzen im Sinne des Screening-Leitprinzips „Nutzen maximieren, Schaden minimieren“ aufzeigt.

## Fazit

Die Neugeborenenzeit, während der das NBS stattfindet, ist eine einzigartige, vulnerable und prägende Phase für junge Familien, weshalb das Prinzip der Schadensvermeidung hier einen besonders hohen Stellenwert haben sollte. Obwohl das NBS in Deutschland mit einer hohen Vollständigkeit und frühen Diagnosestellungen insgesamt erfolgreich umgesetzt wird, zeigen sich im Rahmen des hier vorgestellten Forschungsprojekts auch Schwächen und Handlungsbedarf. Erarbeitete Vorschläge und Empfehlungen zur Weiterentwicklung des NBS wurden in einem Konzeptpapier festgehalten, das Ansätze für eine dem aktuellen Forschungsstand entsprechende Weiterentwicklung des NBS aufzeigt. Die wichtigsten Komponenten des Konzeptes betreffen die Organisation des Screenings im Sinne eines integrierten Public-Health-Programms durch eine zentrale Koordination mit kontinuierlichem Qualitätsmanagement, den Aufbau einer datenschutzkonformen digitalen Infrastruktur und die Etablierung von schlanken Trackingstrukturen. Auch praktikable Vorschläge zu einzelnen Prozesskomponenten wie zu einer möglichst wenig traumatisierenden Befundmitteilung wurden erarbeitet. Jede Weiterentwicklung des NBS muss die bestehenden Rahmenbedingungen und die sich ändernden gesellschaftlichen Anforderungen an die Infrastruktur und Prozesse im Gesundheitssystem berücksichtigen. Ein NBS-Programm, dessen Gesamtnutzen den Schaden überwiegt, soll für jedes Neugeborene zu einem bestmöglichen Start ins Leben beitragen.

### Infobox Konzeptvorschläge zur Weiterentwicklung des Neugeborenen-Screenings aus Trockenblut (NBS) in Deutschland

- NBS als *integriertes Public-Health-Programm *mit effizienter Organisation und Steuerung des gesamten Screening-Prozesses, um eine möglichst hohe Qualität erreichen zu können
- *Einrichtung einer zentralen Koordinierungsstelle* für Programmkoordination und umfassendes Qualitätsmanagement
- *Digitalisierung *mit dem Ziel einer Optimierung des Screening-Prozesses, z. B. durch Etablierung einer zentralen digitalen Plattform und Einführung einer eineindeutigen ID für jedes Neugeborene
- *Tracking* zur Sicherstellung einer hohen Teilnahmerate und der Durchführung aller notwendigen Folgeuntersuchungen im Screening-Prozess
- *Mitteilung auffälliger Screening-Befunde* mit möglichst geringer Beunruhigung der Eltern durch Expert*innen aus den Fachzentren, möglichst zeitnah zum Termin der Konfirmationsdiagnostik
- *Konfirmationsdiagnostik *nur in qualifizierten Zentren und Aufnahme aller diagnostizierten Fälle in ein *Register*
- *Dokumentation und Evaluation* mit dem Ziel einer kontinuierlichen Qualitätssicherung des NBS im Sinne eines Public Health Action Cycle

### Supplementary Information





## References

[1] Hoffmann GF, Lindner M, Loeber JG (2014). 50 years of newborn screening. J Inherit Metab Dis.

[2] Gramer G, Hoffmann GF, Nennstiel-Ratzel U (2015). Das erweiterte Neugeborenenscreening.

[3] Jansen ME, Lister KJ, van Kranen HJ, Cornel MC (2017). Policy making in newborn screening needs a structured and transparent approach. Front Public Health.

[4] González-Irazabal Y, Hernandez de Abajo G, Martínez-Morillo E (2021). Identifying and overcoming barriers to harmonize newborn screening programs through consensus strategies. Crit Rev Clin Lab Sci.

[5] Mütze U, Garbade SF, Gramer G (2020). Long-term outcomes of individuals with metabolic diseases identified through newborn screening. Pediatrics.

[6] Nennstiel-Ratzel U, Lüders A, Blankenstein O (2015). Neugeborenenscreening: ein Paradebeispiel für effektive Sekundärprävention. Bundesgesundheitsblatt Gesundheitsforschung Gesundheitsschutz.

[7] Lüders A, Blankenstein O, Brockow I (2021). Neugeborenen-Screening auf angeborene Stoffwechsel- und Hormonstörungen. Ergebnisse der Jahre 2006–2018 aus Deutschland. Dtsch Ärztebl Int.

[8] Zimmer K-P (2021). Neugeborenen-Screening: noch Platz für Verbesserungen. Editorial. Dtsch Ärztebl Int.

[9] Gemeinsamer Bundesausschuss (G-BA) (2021) Richtlinie des Gemeinsamen Bundesausschusses über die Früherkennung von Krankheiten bei Kindern (Kinder-Richtlinie). zuletzt geändert am 16. September 2021, veröffentlicht im Bundesanzeiger AT 03.11.2021 B4, in Kraft getreten am 1. Januar 2022. https://www.g-ba.de/richtlinien/15/. Zugegriffen: 3. Febr. 2022

[10] Gesellschaft für Neonatologie und Pädiatrische Intensivmedizin (GNPI) (2019) AWMF-S2k-Leitlinie 024/012 „Neugeborenen-Screening auf angeborene Stoffwechselstörungen, Endokrinopathien, schwere kombinierte Immundefekte (SCID), Sichelzellkrankheit, 5q-assoziierte spinale Muskelatrophie (SMA) und Mukoviszidose“. https://www.awmf.org/leitlinien/detail/ll/024-012.html. Zugegriffen: 7. März 2022

[11] Bundesgesundheitsministerium (2009) Gesetz über genetische Untersuchungen bei Menschen (Gendiagnostikgesetz, GenDG). https://www.bundesgesundheitsministerium.de/service/begriffe-von-a-z/g/gendiagnostikgesetz.html. Zugegriffen: 9. Febr. 2022

[12] Nicholls SG, Wilson BJ, Etchegary H (2014). Benefits and burdens of newborn screening: public understanding and decision-making. Per Med.

[13] McCabe ERB (2014). Newborn screening: a complex system that requires a culture of safety. Mol Genet Metab.

[14] Therrell BL, Padilla CD, Loeber JG (2015). Current status of newborn screening worldwide: 2015. Semin Perinatol.

[15] Cornel M, Rigter T, Weinreich S et al (2011) Newborn screening in Europe. Expert opinion document. https://www.isns-neoscreening.org/guidelines/. Zugegriffen: 15. Okt. 2021

[16] Goldenberg AJ, Comeau AM, Grosse SD (2016). Evaluating harms in the assessment of net benefit: a framework for newborn screening condition review. Matern Child Health J.

[17] WHO (2020) Vorsorgeuntersuchung und Screening: ein kurzer Leitfaden. Wirksamkeit erhöhen, Nutzen maximieren und Schaden minimieren. https://apps.who.int/iris/bitstream/handle/10665/330853/9789289054805-ger.pdf. Zugegriffen: 12. Nov. 2021

[18] Anderson R, Rothwell E, Botkin JR (2011). Newborn screening: ethical, legal, and social implications. Annu Rev Nurs Res.

[19] Andermann A, Blancquaert I, Beauchamp S, Déry V (2008). Revisiting Wilson and Jungner in the genomic age: a review of screening criteria over the past 40 years. Bull World Health Organ.

[20] Cornel MC, Rigter T, Jansen ME, Henneman L (2021). Neonatal and carrier screening for rare diseases: how innovation challenges screening criteria worldwide. J Community Genet.

[21] van Dijk T, Kater A, Jansen M (2021). Expanding neonatal bloodspot screening: a multi-stakeholder perspective. Front Pediatr.

[22] Wilson J, Jungner G (1968) PRINCIPLES AND PRACTICE OF SCREENING FOR DISEASE. https://apps.who.int/iris/handle/10665/37650. Zugegriffen: 11. Nov. 2021

[23] Andermann A, Blancquaert I, Beauchamp S, Costea I (2011). Guiding policy decisions for genetic screening: developing a systematic and transparent approach. Public Health Genomics.

[24] Dobrow MJ, Hagens V, Chafe R, Sullivan T, Rabeneck L (2018). Consolidated principles for screening based on a systematic review and consensus process. CMAJ (Ottawa).

[25] Deutsche Gesellschaft für Neugeborenenscreening e. V. (DGNS) DGNS Screeningreports. https://www.screening-dgns.de/reports.php. Zugegriffen: 13. Apr. 2022

[26] Mayring P (2007). Qualitative Inhaltsanalyse. Grundlagen und Techniken.

[27] Odenwald B, Brockow I, Hanauer M, Lüders A, Nennstiel U (2023). Is our newborn screening good enough? In search of the perfect newborn blood spot screening (NBS) programme: a literature review of NBS structures and procedures. Int J Neonatal Screen.

[28] Clinical and Laboratory Standards Institute (CLSI) (2013). NBS02-A2: newborn screening follow-up; approved guideline—second edition.

[29] Wilcken B (2012). Screening for disease in the newborn: the evidence base for blood-spot screening. Pathology.

[30] Malvagia S, Forni G, Ombrone D, la Marca G (2020). Development of strategies to decrease false positive results in newborn screening. Int J Neonatal Screen.

[31] Gramer G, Hoffmann GF (2022). Second-tier strategies in newborn screening—potenzial and limitations. medgen.

[32] Hall PL, Wittenauer A, Hagar A (2020). Post-analytical tools for the triage of newborn screening results in follow-up can reduce confirmatory testing and guide performance improvement. Int J Neonatal Screen.

[33] Loeber JG, Platis D, Zetterström RH (2021). Neonatal screening in Europe revisited: an ISNS perspective on the current state and developments since 2010. Int J Neonatal Screen.

[34] Cornel MC, Rigter T, Weinreich SS (2013). A framework to start the debate on neonatal screening policies in the EU: an expert opinion document. Eur J Hum Genet.

[35] Moody L, Atkinson L, Kehal I, Bonham JR (2017). Healthcare professionals’ and parents’ experiences of the confirmatory testing period: a qualitative study of the UK expanded newborn screening pilot. BMC Pediatr.

[36] Chudleigh J, Ren CL, Barben J, Southern KW (2019). International approaches for delivery of positive newborn bloodspot screening results for CF. J Cyst Fibros.

[37] Gendiagnostik-Kommission (GEKO) beim Robert Koch-Institut (2022) Tätigkeitsbericht der Gendiagnostik-Kommission. Vierter Bericht gemäß § 23 Abs. 4 Gendiagnostikgesetz (GenDG) für den Zeitraum vom 01.01.2019 bis 31.12.2021. https://www.rki.de/geko-bericht. Zugegriffen: 31. Aug. 2022

[38] Brockow I, Nennstiel U (2019). Parents’ experience with positive newborn screening results for cystic fibrosis. Eur J Pediatr.

[39] Liebl B, Nennstiel-Ratzel U, von Kries R (2002). Expanded newborn screening in Bavaria: tracking to achieve requested repeat testing. Prev Med.

[40] Deutsche Gesellschaft für Epidemiologie (DGEpi) (2018) Leitlinien und Empfehlungen zur Sicherung von Guter Epidemiologischer Praxis (GEP). https://www.dgepi.de/de/berichte-und-publikationen/leitlinien-und-empfehlungen/. Zugegriffen: 2. März 2022

[41] Deutsche Gesellschaft für Kinder- und Jugendmedizin (DGKJ) (2019) AWMF-S1-Leitlinie 027-021 „Konfirmationsdiagnostik bei Verdacht auf angeborene Stoffwechselkrankheiten aus dem Neugeborenenscreening“. https://www.awmf.org/leitlinien/detail/ll/027-021.html. Zugegriffen: 20. Apr. 2022

[42] Deutscher Ethikrat (2018) Herausforderungen im Umgang mit seltenen Erkrankungen. AD-HOC-EMPFEHLUNG. https://www.ethikrat.org/publikationen/publikationsdetail/?tx_wwt3shop_detail%5Bproduct%5D=116&tx_wwt3=&cHash=b3e78fc99b523a5226a74aca8f971b95. Zugegriffen: 13. Dez. 2021

[43] Nationales Aktionsbündnis für Menschen mit Seltenen Erkrankungen (NAMSE) (2013) Nationaler Aktionsplan für Menschen mit Seltenen Erkrankungen. Handlungsfelder, Empfehlungen und Maßnahmenvorschläge. https://www.namse.de/. Zugegriffen: 3. Febr. 2022

[44] Wasserstein MP (2016). Long-term follow-up in newborn screening: the role of collaboration. Genet Med.

[45] Bonham JR (2013). Impact of new screening technologies: should we screen and does phenotype influence this decision?. J Inherit Metab Dis.

[46] Miller FA, Cressman C, Hayeems R (2015). Governing population screening in an age of expansion: the case of newborn screening. Can J Public Health.

[47] Boyle CA, Bocchini JA (2014). Reflections on 50 years of newborn screening. Pediatrics.

[48] Ruckstuhl B, Somaini B, Twisselmann W (2008) Förderung der Qualität in Gesundheitsprojekten. Der Public Health Action Cycle als Arbeitsinstrument. http://www.quint-essenz.ch/de/files/Foerderung_der_Qualitaet.pdf. Zugegriffen: 3. Dez. 2021

[49] Murko S, Gramer G, Santer R (2023). Neugeborenenscreening auf angeborene Störungen des Stoffwechsels, des Hormon-, des Blut, des Immunsystems und des neuromuskulären Systems. Kinder Jugendarzt.

[50] Robert Koch-Institut (RKI) (2020). Richtlinie der Gendiagnostik-Kommission (GEKO) für die Anforderungen an die Durchführung genetischer Reihenuntersuchungen gemäß § 23 Abs. 2 Nr. 6 GenDG. Bundesgesundheitsblatt Gesundheitsforschung Gesundheitsschutz.

[51] Nennstiel U, Tönnies H (2020). Grundlagen und aktueller Stand des Neugeborenen-Screenings auf angeborene Störungen des Stoffwechsels, des Hormon- und Immunsystems in Deutschland. medgen.

