# Supplementary material for: Newborn blood spot screening (NBS) in Germany: Status quo and presentation of a concept for further development
Source: Bundesgesundheitsblatt Gesundheitsforschung Gesundheitsschutz. 2023 Oct 10;66(11):1195–204. [Article in German] doi: 10.1007/s00103-023-03771-8 (PMC10622373; doi:10.1007/s00103-023-03771-8)
Supplement: Supplementary file 1 [file 103_2023_3771_MOESM1_ESM.pdf]

Bayerisches Landesamt für
Gesundheit und Lebensmittelsicherheit

LUDWIG-MAXIMILIANS-UNIVERSITÄT MÜNCHEN

PETTENKOFER SCHOOL OF PUBLIC HEALTH

CHARITÉ UNIVERSITÄTSMEDIZIN BERLIN

Organisatorische Weiterentwicklung des erweiterten Neugeborenen-Screenings (ENS)

Konzept

der Bewerbergemeinschaft LGL, LMU, Charité

im Auftrag des GKV-Spitzenverbands

Stand: 10.08.2022

Korrespondierende Autorin:
Dr. med. Uta Nennstiel MPH
Bayerisches Landesamt für Gesundheit und Lebensmittelsicherheit (LGL)
Veterinärstr. 2
85764 Oberschleißheim
E-Mail: uta.nennstiel@lgl.bayern.de

Weitere Autoren:
Bayerisches Landesamt für Gesundheit und Lebensmittelsicherheit (LGL)
Dr. med. Inken Brockow MPH
Dr. med. Birgit Odenwald MPH

Ludwig-Maximilians-Universität (LMU) München: Pettenkofer School of Public Health
Dr. rer. biol. hum. Michaela Coenen MPH
Veronika Throner MPH

Charité-Universitätsmedizin Berlin
Dr. med. Oliver Blankenstein

Ethische Beratung: PD Dr. phil. Andreas Vieth
Philosophisches Seminar, Universität Münster

Juristische Beratung: Dr. jur. Rudolf Ratzel, Fachanwalt für Medizinrecht
Seniorpartner: Ratzel Rechtsanwälte

Zusammenfassung

Das Neugeborenen-Screening (NBS) aus Trockenblut (Testkarten) gilt weltweit als die erfolgreichste Maßnahme der Sekundärprävention im Kindesalter. Durch die Früherkennung behandelbarer angeborener Krankheiten können Gesundheitsfolgen wie schwere lebenslange Behinderung vermieden und Todesfälle verhindert werden. Voraussetzung ist eine Reihenuntersuchung (Screening) der gesamten Neugeborenen-Population in den ersten Lebenstagen, um die wenigen betroffenen Kinder zu finden – nur eines von etwa 750 Neugeborenen ist betroffen. Das NBS ist deshalb in erster Linie eine bevölkerungsmedizinische Maßnahme, die nach definierten Kriterien in einem von der Elternaufklärung bis zur Nachsorge gut strukturierten und organisierten Programm durchgeführt werden sollte.

In vielen europäischen und angloamerikanischen Ländern wird das NBS allen Neugeborenen im Sinne einer Public-Health-Maßnahme angeboten, deren wichtigster Nutzen der direkte gesundheitliche Benefit für Kinder mit einer im NBS diagnostizierten Zielkrankheit ist. Allerdings kann Screening auch schaden. Daher gilt es durch die Etablierung eines Screening-Programms, den „Nutzen zu maximieren“ und „möglichen Schaden zu minimieren“. Dieses Programm sollte von der Aufklärung bis zur Diagnosestellung optimierte standardisierte Abläufe vorsehen. Solche Prozesse und Verfahren sind grundsätzlich nicht zu einem bestimmten Zeitpunkt „fertig und optimiert“, sie müssen vielmehr in einem fortlaufenden Verfahren im Sinne eines „Public Health Action Cycle“ überprüft und angepasst werden.

In Deutschland wird das „Erweiterte Neugeborenen-Screening“ (ENS) seit 2005 sehr erfolgreich umgesetzt. Dies zeigen die Qualitätsberichte der Deutschen Gesellschaft für Neugeborenenscreening (DGNS-Reporte) sowie eine Gesamtübersicht über das Screening der Jahre 2006-2018. Geregelt sind die Grundzüge des Screeningverfahrens durch den Gemeinsamen Bundesausschuss (G-BA) in der Kinder-Richtlinie. Das Screening unterliegt darüber hinaus dem Gendiagnostikgesetz (GenDG). In elf Screeninglaboren, die zumeist bundeslandübergreifend arbeiten, wurden die Testkarten bis 2016 auf zehn metabolische und zwei endokrinologische Zielkrankheiten analysiert. Seither wurde das ENS um fünf Zielkrankheiten erweitert. Erste Auswertungen des 2016 neu eingeführten Neugeborenen-Screenings auf Mukoviszidose (CF) und die Erfahrungen bei der Erweiterung des Screenings auf schwere kombinierte Immundefekte (SCID), Sichelzellkrankheit und Spinale Muskelatrophie (SMA) geben wichtige Hinweise zu den Herausforderungen, die bei der Einführung weiterer Zielkrankheiten zu erwarten sind. Vor diesem Hintergrund wurde die Bewerbergemeinschaft beauftragt, Vorschläge für die Weiterentwicklung der Strukturen und Prozesse des ENS zu entwickeln, damit auch bei der Einführung neuer Zielkrankheiten die Qualität des gesamten Screening-Prozesses gewährleistet und nachhaltig gesichert werden kann.

Für die Entwicklung des Konzeptes wurde zunächst der Status quo des ENS in Deutschland erhoben (Arbeitspaket 1). Dieses Arbeitspaket enthält eine vertiefte Analyse der Screeningdaten (DGNS-Daten) von 2006 bis 2019, eine Online-Befragung der Geburtskliniken, Einzelinterviews mit Vertreter*innen aller Screeninglabore, der Geburtskliniken, pädiatrischen Praxen und Hebammen sowie Gruppendiskussionen mit Vertreter*innen der Screeninglabore und Expert*innen der spezialisierten Zentren für die Abklärung der pathologischen Screeningbefunde (Konfirmations-diagnostik). Gleichzeitig wurde eine systematische Literaturrecherche zum Neugeborenen-Screening aus Trockenblut (NBS) sowie zu Best-Practice-Modellen in anderen Ländern durchgeführt (Arbeitspaket 2). Die Strukturen und Prozesse des ENS wurden auf wissenschaftliche Aktualität, Effektivität, praktische Umsetzbarkeit und Weiterentwicklungsbedarf geprüft. Dabei zeigte sich, dass

im ENS in Deutschland nicht alle Anforderungen an ein „Screening-Programm“ im Sinne der aktuellen Screening-Kriterien erfüllt sind. So fehlen eine zentrale Prozess-Koordination, eine kontinuierliche Qualitätssicherung mit Mechanismen zur Optimierung des Programms und zur Minimierung potenzieller Risiken des Screenings sowie eine obligate Evaluation inklusive der Vorgaben für die hierfür notwendige Dokumentation.

Auf Basis der Ergebnisse aus Arbeitspaket 1 und 2 wurde das hier vorgestellte Konzept entwickelt. Die Konzeptvorschläge gehen grundsätzlich von geltendem Recht aus, machen jedoch hinsichtlich der organisatorischen Abläufe Vorschläge zu einer strukturellen Verbesserung, die teilweise eine Anpassung der einschlägigen Normen erfordern. Die wichtigsten Punkte des Konzeptes betreffen die Organisation des Screenings als ein Programm mit zentraler Koordination und einem kontinuierlichen Qualitätsmanagement, Digitalisierung, Tracking, Aufklärung und Befundmitteilung sowie Qualitätssicherung im Screeninglabor.

Vorschläge für die Weiterentwicklung der Strukturen und Prozesse des ENS

Zentrale Koordinierungsstelle und Qualitätsmanagement

Optimale Screeningergebnisse können nur bei effizienter Organisation und Steuerung des gesamten Screening-Prozesses erzielt werden. Dafür muss das Neugeborenen-Screening für alle Zielkrankheiten als integriertes Public-Health-Programm geregelt werden, das alle Prozessschritte von der Aufklärung bis zur Konfirmationsdiagnostik sowie die Qualitätssicherung und Evaluation umfasst. Diese Aufgabe könnte optimalerweise von einer zentralen Koordinierungsstelle für ganz Deutschland übernommen werden. Denkbar sind auch regionale Koordinierungsstellen, die eng miteinander kooperieren. Entscheidend ist, dass Organisation und Steuerung sowie Qualitätsmanagement für ganz Deutschland einheitlich geregelt und umgesetzt werden und Brüche im Screening-Prozess, z.B. bei Einbindung mehrerer Screeninglabore oder Screeningpfade über Bundeslandgrenzen hinweg, vermieden werden. Vor diesem Hintergrund sind die Hauptaufgaben der Koordinierungsstelle in der Programmkoordination und Qualitätssicherung des ENS zu sehen. Darunter fallen die regelmäßige Analyse der Qualitätsberichte (DGNS-Report) sowie die Identifikation von Qualitätsmängeln, Anpassungs- und Optimierungsbedarf. In Abhängigkeit vom Thema könnten Vertreter*innen aus den Screeninglaboren, behandelnde Ärzte oder Ärztin (Expert*innen) und ggf. weitere Stakeholder partizipativ in den Diskurs zur Qualitätsverbesserung im Sinne eines „Public Health Action Cycle“ eingebunden werden. Die Koordinationsstelle könnte eine zentrale Datenplattform betreuen und eine einheitliche und eineindeutige Screening-ID verwalten sowie die Öffentlichkeitsarbeit übernehmen.

Digitalisierung

Digitale Übermittlungs- oder Abfragewege können den Screening-Prozess vereinfachen, beschleunigen und absichern. Dies gilt z.B. für die traditionell auf der Testkarte manuell eingetragenen Angaben zum Kind, die Nachverfolgung des Versands der Testkarten bis zum Eingang im Labor, die Übermittlung der Screeningbefunde und der Ergebnisse der Konfirmationsdiagnostik sowie die Langzeitbeobachtung bzw. Patientenregister. Eine zentrale Datenplattform (Datenbank) ermöglicht ein kontinuierliches Monitoring des NBS. Sie kann die Qualitätssicherung und Evaluation des NBS effektiver gestalten und helfen, das Tracking flächendeckend umzusetzen.

Eine zentrale bundesweite Datenplattform sollte auf einem sicheren Server mit rollenbasierter Zugriffskontrolle und einem datenschutzkonformen Schlüsselkonzept etabliert werden und pseudo-

nyme Daten aus dem gesamten Screening-Prozess enthalten. Eine deutschlandweit einheitliche eineindeutige Screening-ID würde für jedes Neugeborene kurz nach der Geburt zusammen mit einer Kennung der Geburtseinrichtung bzw. der Hebamme in diese zentrale Plattform eingegeben werden. Die Blutentnahme, der Eingang der Testkarte im Labor und die Befunde aus Screening und Konfirmationsdiagnostik könnten der Screening-ID digital zugeordnet werden. So könnte das Tracking labor- und bundeslandübergreifend effektiv umgesetzt und das Qualitätsmanagement vereinfacht werden.

Tracking

Eine systematische Erfassung der Zielpopulation und die Abklärung aller kontroll- und wiederholungsbedürftigen Befunde ist die Grundbedingung für eine hohe Effektivität des Neugeborenen-Screenings. Um dies zu erreichen, ist ein Tracking notwendig, d.h. der Eingang einer Testkarte im Labor wird überprüft, Eltern werden ggf. auf das fehlende Screening aufmerksam gemacht und an nicht durchgeführte Folgeuntersuchungen erinnert.

Das Tracking fehlender Screeninguntersuchungen könnte über eine zentrale Datenplattform automatisiert werden, indem der eineindeutigen Screening-ID von geborenen Kindern die in den Laboren eingegangenen Testkarten zugeordnet und standardisierte Anschreiben an Eltern nicht gescreenter Kinder erzeugt werden. Eine Verknüpfung mit Personendaten würde z.B. über eine Vertrauensstelle abgewickelt und wäre nur dann vorgesehen, wenn ein Anschreiben nötig ist.
Das Tracking der notwendigen Folgeuntersuchungen sollte von den Screeninglaboren oder kooperierenden Screeningzentren übernommen werden.

Struktur und Qualitätssicherung im Screeninglabor

Der Erfolg von NBS-Programmen ist maßgeblich von der Qualität der Screeninglabore und ihrer Verfahren geprägt. Die Zielwerte und Benchmarks, die die Qualität und Wirksamkeit der Laborverfahren gewährleisten, sollten auf der Ebene der Programmkoordination festgelegt werden. Eine Abstimmung zwischen den Screeninglaboren in Deutschland ist auf Programmebene erforderlich. Entscheidend sind nicht nur valide Analyseverfahren, sondern auch die Einordnung der Testergebnisse. Dabei soll durch multiple Marker, biochemische oder molekulargenetische mehrstufige Testverfahren und regelmäßige Überprüfung und ggf. Korrektur der Grenzwerte (Cut-off) der Anteil falsch-positiver Befunde möglichst geringgehalten werden. Diesem Ziel können auch post-analytische digitale Interpretationswerkzeuge dienen, die auf Machine-Learning-Modellierungen bzw. Big-Data-Analysen basieren.

Aufklärung und Befundmitteilung

Die Aufklärung der Eltern gilt als wesentlicher Bestandteil des NBS-Programms. Die Eltern sollen ausreichende Informationen erhalten, um die Notwendigkeit des Screenings zu verstehen, gleichzeitig aber nicht beunruhigt werden. Zunehmend werden gedruckte Materialien als einziges Aufklärungs-Medium kritisch gesehen und innovative digitale Wege der Informationsvermittlung wie Videos oder Online-Informationsseiten als Ersatz oder Ergänzung dazu in Kombination mit einem Aufklärungsgespräch (auch digital oder in der Gruppe) und individueller Rückfragemöglichkeiten empfohlen. Eine pränatale Aufklärung im letzten Trimenon der Schwangerschaft wird als optimal betrachtet.

Ein pathologischer (auffälliger) Screeningbefund bedeutet noch keine Diagnose, sondern zunächst nur einen Krankheitsverdacht, der durch geeignete Methoden zeitnah bestätigt oder ausgeschlossen

werden muss (z.B. zweite Testkarte oder Konfirmationsdiagnostik). Die Mitteilung kontrollbedürftiger Befunde an die Eltern sollte am besten von einer geschulten Person aus dem Screeninglabor übernommen werden. Bei hochpathologischen Befunden, die einer Konfirmationsdiagnostik bedürfen, wäre die Befundmitteilung an die Eltern durch Expert*innen der jeweiligen Fachrichtung aus den spezialisierten Zentren (Behandlungszentren) optimal. Für die Eltern wichtig sind konkrete Angaben zum weiteren Vorgehen sowie das Angebot schriftlicher Materialien über das jeweilige Krankheitsbild oder entsprechende Hinweise auf zuverlässige Internetseiten.

Konfirmationsdiagnostik

Eine deutschlandweite Vereinheitlichung des Prozessablaufs nach einem auffälligen Screeningbefund mit der Zuweisung zu einem qualifizierten Zentrum und der Rückmeldung der Befunde aus der Konfirmationsdiagnostik wird dringend empfohlen. Diese Zentren sollten nach objektiv festgelegten und transparenten Qualitätskriterien ausgewählt und regelmäßig reevaluiert werden.

Dokumentation und Evaluation

Für die Qualitätssicherung des ENS sind eine standardisierte Dokumentation, regelmäßige Qualitätsberichte und unter definierten Bedingungen eine Evaluation des gesamten Screening-Prozesses notwendig. Dabei aufgedeckte Schwächen und Qualitätsdefizite müssen im Sinne eines „Public Health Action Cycle“ innerhalb festgesetzter Fristen bearbeitet werden und Konsequenzen für die Qualitätsverbesserung in den betreffenden Programmschritten zur Folge haben.

Die Vorschläge in diesem Konzept zur Weiterentwicklung des ENS dienen dazu, auch bei der Einführung neuer Zielkrankheiten die Qualität des gesamten Screenings zu gewährleisten, nachhaltig zu sichern und dem aktuellen wissenschaftlichen Stand gerecht zu werden. Essentiell ist hier die Regelung aller Screening-Komponenten und -Prozesse im Sinne eines qualitätsgesicherten Public-Health-Programmes, um die Wirksamkeit zu erhöhen, den Nutzen zu maximieren und den Schaden zu minimieren. Die wesentlichen Elemente sind in der folgenden Tabelle nochmals zusammengefasst.

Zusammenfassung der Konzeptvorschläge

**Einrichtung einer zentralen Koordinierungsstelle** zur Programmkoordination und Qualitätssicherung des ENS mit folgenden Aufgaben:<br>• Öffentlichkeitsarbeit, Informationsmaterialien, Webseite, Leitlinien/SOPs, Schulungen, Schulungsmaterialien<br>• Organisation und zentrales Qualitätsmanagement des gesamten ENS-Prozesses als Public-Health-Aufgabe<br>• Digitalisierung und Verwaltung einer bundesweiten zentralen Datenplattform<br>• Kooperation mit Stakeholdern, Beratungs- und Fachgremien
**Digitalisierung** mit dem Ziel einer Optimierung des Screening-Prozesses, insbesondere des Trackings und der Evaluation<br>• Etablierung einer zentralen bundesweiten digitalen Plattform auf einem sicheren Server mit rollenbasierter Zugriffskontrolle und einem datenschutzkonformen Schlüsselkonzept<br>• Zuordnung einer eineindeutigen Screening-ID als Pseudonym und Schlüsselvariable für jedes Neugeborene bei Geburt mit Barcode-Etiketten im Kinderuntersuchungsheft<br>• Datenschutzkonforme Dokumentation jeder Untersuchung im Screening-Prozess unter dieser Screening-ID
**Tracking** zur Sicherstellung einer hohen Teilnahmerate und der Durchführung aller notwendigen Folgeuntersuchungen im Screening-Prozess<br>• Sicherstellung einer hohen Teilnahme über eine eineindeutige Screening-ID, mit der jedes Kind bei Geburt in einer zentralen digitalen Plattform erfasst wird<br>• Tracking der Wiederholungs- und Kontrolluntersuchungen sowie der Konfirmationsdiagnostik durch lokale Screeningzentren oder Befundkoordinator*innen der Labore ggf. in Zusammenarbeit mit einer zentralen Koordinierungsstelle
**Struktur und Qualitätssicherung im Screeninglabor**<br>• Regelmäßige Anpassung der vorgesehenen Analysemethoden<br>• Einsatz mehrstufiger Testverfahren (Second-Tier-Verfahren)<br>• Laborübergreifende Qualitätssicherung der Analytik (z.B. Ringversuche, Qualitätszirkel) mit Konsequenzen bei Nicht-Erfüllung der festgelegten Qualitätsziele<br>• Qualitätssicherung bei der Befundung durch Anwendung von postanalytischen multivariaten digitalen Interpretationswerkzeugen mit dem Ziel einer hohen Sensitivität und Spezifität (möglichst niedrige Recall-Rate)<br>• Regelmäßige Qualitätsberichte und Evaluation des Prozesses (Analyse und Befundung) mit ggf. Maßnahmen zur Verbesserung der Qualität

<u>Aufklärung</u> mit dem Ziel einer informierten Einwilligung (Informed Consent) der Eltern

- Pränatale mündliche Aufklärung unterstützt durch schriftliche oder digitale Materialien
- Aufklärung durch Ärztinnen und Ärzte, ggf. geschultes Fachpersonal mit ärztlicher Rückfragemöglichkeit
- Zentrale Bereitstellung von Leitfäden und Schulungen für aufklärendes Fachpersonal
- Aufklärungsmaterialien mit gut verständlicher Information, ergänzt durch weiterführendes Material z.B. auf Webseite/ durch Video
- Basisinhalte der Aufklärung: Ziele und Nutzen des ENS, Screening-Prozess, mögliche Ergebnisse und ihre Bedeutung

<u>Mitteilung auffälliger Screeningbefunde</u> mit möglichst geringer Beunruhigung der Eltern

- Mitteilung hochpathologischer Befunde an die Eltern durch Expert*innen aus den Fachzentren, möglichst zeitnah zum Termin der Konfirmationsdiagnostik
- Mitteilung grenzwertig auffälliger Befunde oder notwendiger Wiederholungskarten durch geschultes Fachpersonal der Screeninglabore
- Bereitstellung von Informationen auf einer zentralen Screening-Website
    - Für Eltern: Was bedeutet ein auffälliges Screening?
    - Schulungs- und Informationsmaterial für Fachpersonal

<u>Konfirmationsdiagnostik</u>

- Leitliniengerechte Konfirmationsdiagnostik nur in qualifizierten Zentren, die nach definierten und transparenten Kriterien von den Fachgesellschaften benannt und reevaluiert werden
- Rückmeldung der Ergebnisse der Konfirmationsdiagnostik an eine zentrale Stelle oder an die Labore
- Aufnahme aller diagnostizierten Fälle in ein Register

<u>Dokumentation und Evaluation</u> mit dem Ziel einer kontinuierlichen Qualitätssicherung des ENS im Sinne eines „Public Health Action Cycle“

- Standardisierte Dokumentation und regelmäßige Evaluation (mit Erstellung von Qualitätsberichten, z.B. DGNS-Report) des gesamten Screening-Prozesses anhand in der Kinder-Richtlinie festgelegter Qualitätsparameter
- Bei Nichterreichen der Zielparameter zeitnahe Konsequenzen zur Qualitätsverbesserung
- Supervision durch Koordinierungsstelle

Inhaltsverzeichnis

1 Hintergrund ... 1

2 Methoden und Datengrundlage ... 4

2.1 Arbeitspaket 1: Analyse der aktuellen Strukturen und Prozesse des ENS ... 4

2.2 Arbeitspaket 2: Systematische Literaturrecherche und Suche nach Best-Practice-Modellen ... 7

2.3 Arbeitspaket 3: Konzeptentwicklung ... 10

3 Ergebnisse ... 11

3.1 Perspektiven für eine Weiterentwicklung des Neugeborenen-Screenings ... 12

3.2 Elternaufklärung und Einwilligung ... 13

3.3 Struktur und Qualitätssicherung im Screeninglabor ... 18

3.4 Mitteilung des (auffälligen) Screeningbefundes ... 26

3.5 Konfirmationsdiagnostik ... 30

3.6 Tracking ... 32

3.7 Dokumentation und Evaluation ... 36

3.8 Digitalisierung im Screening-Prozess ... 38

3.9 Zentrale Koordination ... 42

4 Literaturverzeichnis ... 49

Abbildungsverzeichnis

Abbildung 1 Regionale Verteilung der Screeningproben auf die Screeninglabore 2019 2

Abbildung 2 Neugeborenen-Screening in Deutschland 3

Abbildung 3 Daten zum Screening 2006-2019 5

Abbildung 4 Grafische Darstellung der systematischen Literaturrecherche 9

Abbildung 5 Konzeptentwicklung... 11

Abbildung 6 Mehrstufige Testverfahren (Second-Tier-Strategie) [42] 19

Abbildung 7 Verlauf der Recall-Raten über die Zeit und nach Krankheiten 21

Abbildung 8 Recall-Raten AGS aus DGNS 2019; Einfluss Second-Tier-Verfahren 21

Abbildung 9 Spannweite der Recall-Raten zwischen den Laboren 22

Abbildung 10 Anteil der innerhalb von 24 Stunden mitgeteilten Befunde, Spannweite der Labore ... 22

Abbildung 11 Anteil eingegangener Wiederholungs- und Kontrolluntersuchungen........................... 34

Abbildung 12 Zeitraum von der Blutabnahme bis zum Eingang im Labor 44

Abbildung 13 Spannweite der Versandzeiten zwischen den Laboren 44

Abbildung 14 „Public Health Action Cycle“ 47

Abkürzungsverzeichnis

AGS	Adrenogenitales Syndrom
APHL	Association of Public Health Laboratories, Verband der Laboratorien des öffentlichen Gesundheitswesens (USA)
AWMF-Leitlinie	Leitlinie der Arbeitsgemeinschaft der Wissenschaftlichen Medizinischen Fachgesellschaften
CDC	Centers for Disease Control and Prevention, Institut für Krankheitskontrolle und Prävention (USA)
CF	Cystische Fibrose (Mukoviszidose)
CLIR	Collaborative Laboratory Integrated Reports, digitales Instrument zur Bewertung der Analyseergebnisse
DAkkS	Deutsche Akkreditierungsstelle GmbH
DGKJ	Deutsche Gesellschaft für Kinder- und Jugendmedizin e.V.
DGNS	Deutschen Gesellschaft für Neugeborenenscreening e.V.
DSGVO	Europäische Datenschutzgrundverordnung
DRG	Diagnosis Related Groups, diagnosebezogene Fallgruppierung
ENS	Erweitertes Neugeborenen-Screening
G-BA	Gemeinsamer Bundesausschuss
GenDG	Gendiagnostikgesetz
GKV	Gesetzliche Krankenversicherung
ID	Identifikationsnummer
IQR	Interquartilsabstand
IQWiG	Institut für Qualität und Wirtschaftlichkeit im Gesundheitswesen
ISNS	International Society for Neonatal Screening, Internationale Gesellschaft für Neugeborenen-Screening
IVA	Isovalerianacidämie
K(B)V	Kassenärztliche (Bundes-) Vereinigung
LGL	Bayerisches Landesamt für Gesundheit und Lebensmittelsicherheit
MCAD - Mangel	Medium-Chain-Acyl-CoA-Dehydrogenase-Mangel
(LC) MS/MS	(Liquid-Chromatographie) Tandem-Massenspektrometrie
MSUD	Maple Syrup Urine Disease, Ahornsiruperkrankung
NBS	Newborn Screening, Neugeborenen-Screening
NSQAP	Newborn Screening Quality Assurance Program, Qualitätssicherungsprogramm für das Neugeborenenscreening (USA)
OECD	Organization for Economic Co-operation and Development, Organisation für wirtschaftliche Zusammenarbeit und Entwicklung
RiliBÄK	Richtlinie der Bundesärztekammer zur Qualitätssicherung laboratoriumsmedizinischer Untersuchungen
RIVM	Rijksinstituut voor Volksgezondheid en Milieu, Staatliches Institut für Gesundheit und Umwelt (Niederlande)
SCID	Severe Combined Immunodeficiency, schwerer kombinierter Immundefekt
SGB	Sozialgesetzbuch
SOP	Standard Operating Procedure, Standardvorgehensweise
SSW	Schwangerschaftswochen
WHO	World Health Organization, Weltgesundheitsorganisation

1 Hintergrund

Das Neugeborenen-Screening (newborn screening; NBS) aus Trockenblut (Testkarten) wurde Ende der 1960er Jahre in Deutschland eingeführt und gilt als die erfolgreichste Maßnahme der Sekundärprävention im Kindesalter [1–4]. Durch die Früherkennung behandelbarer angeborener Krankheiten können Gesundheitsfolgen wie schwere lebenslange Behinderung vermieden und Todesfälle verhindert werden. Aus individualmedizinischer Sicht ist dies für die Betroffenen und deren Familien ein großartiger medizinischer Fortschritt. Er setzt allerdings Diagnose und Therapiebeginn vor dem Auftreten von Symptomen, d.h. in den ersten Lebenstagen bis Wochen, voraus. Dies ist nur durch eine Reihenuntersuchung (Screening) der gesamten Neugeborenen-Population in den ersten Lebenstagen möglich. Die Zielkrankheiten des NBS sind selten und treffen in ihrer Gesamtheit eines von etwa 750 Neugeborenen (0,1% der Neugeborenen-Population)[1] [5]. Dies bedeutet wiederum, dass 99,9% der nicht betroffenen „gesunden" Neugeborenen-Population mit untersucht werden müssen, um die wenigen Betroffenen zu entdecken. Das NBS ist deshalb zunächst in erster Linie eine bevölkerungsmedizinische Maßnahme, die nach definierten Kriterien in einem von der Elternaufklärung bis zur Nachsorge gut strukturierten und organisierten Programm durchzuführen ist [6–10].

In vielen europäischen und angloamerikanischen Ländern wird das NBS allen Neugeborenen im Sinne einer Public-Health-Maßnahme angeboten. Wichtigster Nutzen ist der direkte gesundheitliche Benefit für Kinder mit einer im NBS diagnostizierten Zielkrankheit. Hier gilt es, den „Nutzen zu maximieren" durch optimierte standardisierte Abläufe mit Vermeidung von Zeitverzögerungen, denn bei einigen Zielkrankheiten droht bereits in den ersten Lebenswochen eine lebensbedrohliche Stoffwechsel- oder Elektrolytkrise, bei anderen hängt der Erfolg der Behandlung von deren frühzeitigem Beginn ab [11]. Langfristig wird durch die präsymptomatische Behandlung auch die Belastung der Eltern reduziert und die soziale Teilhabe der Kinder in späteren Lebensphasen ermöglicht. Jedes Screening-Programm kann jedoch neben dem großen Nutzen für die betroffene Person auch Schaden verursachen [12]. Z.B. führen falsch positive (auffällige) Befunde zu einer Belastung des Gesundheitssystems und können eine kurz- oder langfristige psychosoziale Belastung der Familien (Beunruhigung, vulnerable child syndrome) auslösen [13–15]. Als zentrales ethisches Leitprinzip für Screening-Programme gilt „Der Gesamtnutzen des Screenings soll den Schaden überwiegen" [16].

Mit der Aufnahme jeder neuen Krankheit wachsen die Anforderungen an die Infrastruktur und Prozesse des Neugeborenen-Screenings. Dies betrifft insbesondere Krankheiten, die neue Analysemethoden in den Laboren und die Einbindung von weiteren spezialisierten Behandlungs- und Nachsorge-Zentren zur Abklärungsdiagnostik und Therapie erfordern. Die einfache Erweiterung des bereits bestehenden Screeningpanels auf Stoffwechselstörungen um eine weitere Störung des Aminosäure-Stoffwechsels, wie z.B. Tyrosinämie, erscheint hingegen eher unproblematisch. Aus diesen Gründen müssen neue Zielkrankheiten für ein Screening sehr sorgfältig und unter Berücksichtigung der durch Andermann [17] aktualisierten Screening-Kriterien von Wilson und Jungner [18] ausgewählt werden. So soll eine mögliche Zielkrankheit ein wichtiges Gesundheitsproblem darstellen, bei der in der Regel zum Zeitpunkt des Screenings noch keine Symptome vorliegen und für die eine anerkannte Therapie für nachweislich erkrankte Patienten verfügbar ist (Screening-Kriterien im Anhang 6, S. 38). Mit einer höheren Zahl an Zielkrankheiten können mehr Kinder vom Nutzen des

[1] Berechnung unter Berücksichtigung der Zielkrankheiten des ENS: Stand 2022

Screenings profitieren, jedoch steigt auch die Zahl von potentiellen Schäden z.B. durch falsch positive und negative oder unklare Befunde. Die Qualität des gesamten Screening-Programms zu berücksichtigen wird als Anforderung besonders auch für alle Programm-Erweiterungen genannt [19–21]. Der Titel des WHO-Leitfadens zu Screening-Programmen von 2020 benennt prägnant das zu beachtende Kernprinzip: „Wirksamkeit erhöhen, Nutzen maximieren und Schaden minimieren“ [22].

Vor diesem Hintergrund wurde die Bewerbergemeinschaft beauftragt, Vorschläge für die Weiterentwicklung der Strukturen und Prozesse des in Deutschland etablierten „Erweiterten Neugeborenen-Screenings“ (ENS) zu entwickeln, um auch bei der Einführung neuer Zielkrankheiten ein hohes Qualitätsniveau des gesamten Screening-Prozesses zu gewährleisten und dieses nachhaltig zu sichern. Die Bewertung möglicher neuer Zielkrankheiten hinsichtlich der Evidenz zur Einführung eines Neugeborenen-Screenings erfolgt in Deutschland durch das Institut für Qualität und Wirtschaftlichkeit im Gesundheitswesen (IQWiG) und ist daher nicht Inhalt dieses Konzeptes.

In Deutschland wurde das ENS auf angeborene Stoffwechselstörungen und Endokrinopathien im Juli 2005 in den Leistungskatalog der Gesetzlichen Krankenkassen aufgenommen und seither um vier zusätzliche Krankheitsentitäten inklusive Mukoviszidose (Cystische Fibrose, CF) erweitert. Das bedeutet, dass Behandlungszentren aus vier weiteren pädiatrischen Fachrichtungen in die Betreuung der Kinder eingebunden werden müssen. Geregelt sind die Grundzüge des Screeningverfahrens in den §§13 bis 28 für das ENS und in den §§29 bis 42 für das CF-Screening der vom Gemeinsamen Bundesausschuss (G-BA) gemäß §26 SGB V beschlossenen Kinder-Richtlinie über die Früherkennung von Krankheiten („Kinder-Richtlinie“) [23]. Darüber hinaus unterliegen das ENS und das CF-Screening den Regelungen des am 01.02.2010 in Kraft getretenen Gendiagnostikgesetzes (GenDG [24]).

Im Rahmen des ENS sollen die Eltern zunächst durch eine Ärztin oder einen Arzt über das Screening aufgeklärt werden und schriftlich in die Durchführung einwilligen. Die Blutprobe wird zwischen 36 und 72 Lebensstunden oder vor einer früheren Entlassung aus der Geburtsklinik abgenommen. In diesem Fall oder bei vor 32 Schwangerschaftswochen (SSW) geborenen Kindern soll das Screening wiederholt werden. Die Testkarten werden dann in eines von elf Screeninglaboren zur Analytik geschickt. Diese Labore arbeiten größtenteils bundeslandübergreifend, auch wenn in einigen Bundesländern die Proben in der Regel nur in Screeninglabore im eigenen bzw. kooperierenden Bundesland geschickt werden (Abbildung 1) [5]. Die Verträge zur Durchführung der Analysen im Rahmen des ENS und die Kosten für die Laborleistung werden jeweils zwischen Klinikverwaltung und Screeninglabor ausgehandelt. Die Finanzierung des ENS und CF-Screenings inklusive der Laboranalytik erfolgt im stationären Bereich im Rahmen der DRG-Geburtenpauschale.

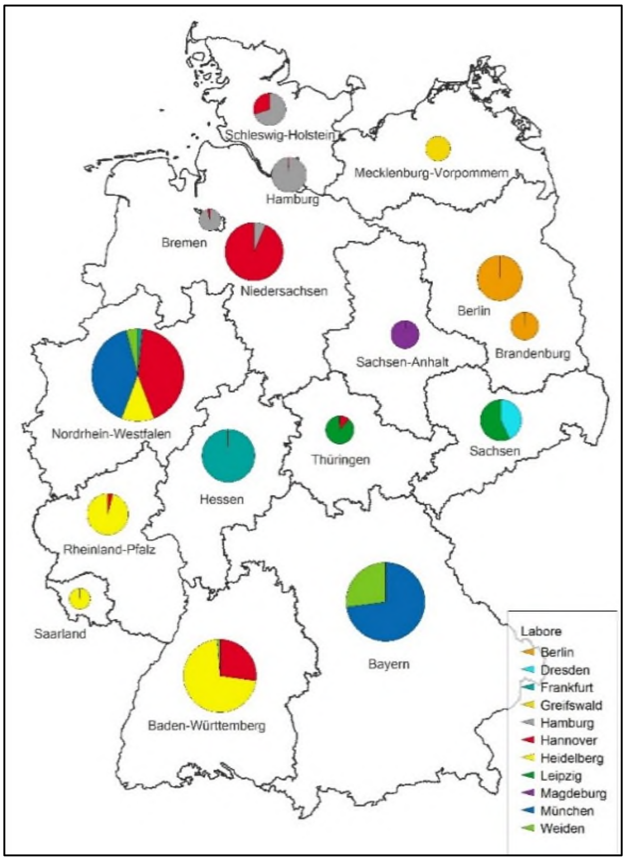

Abbildung 1 Regionale Verteilung der Screeningproben auf die Screeninglabore 2019

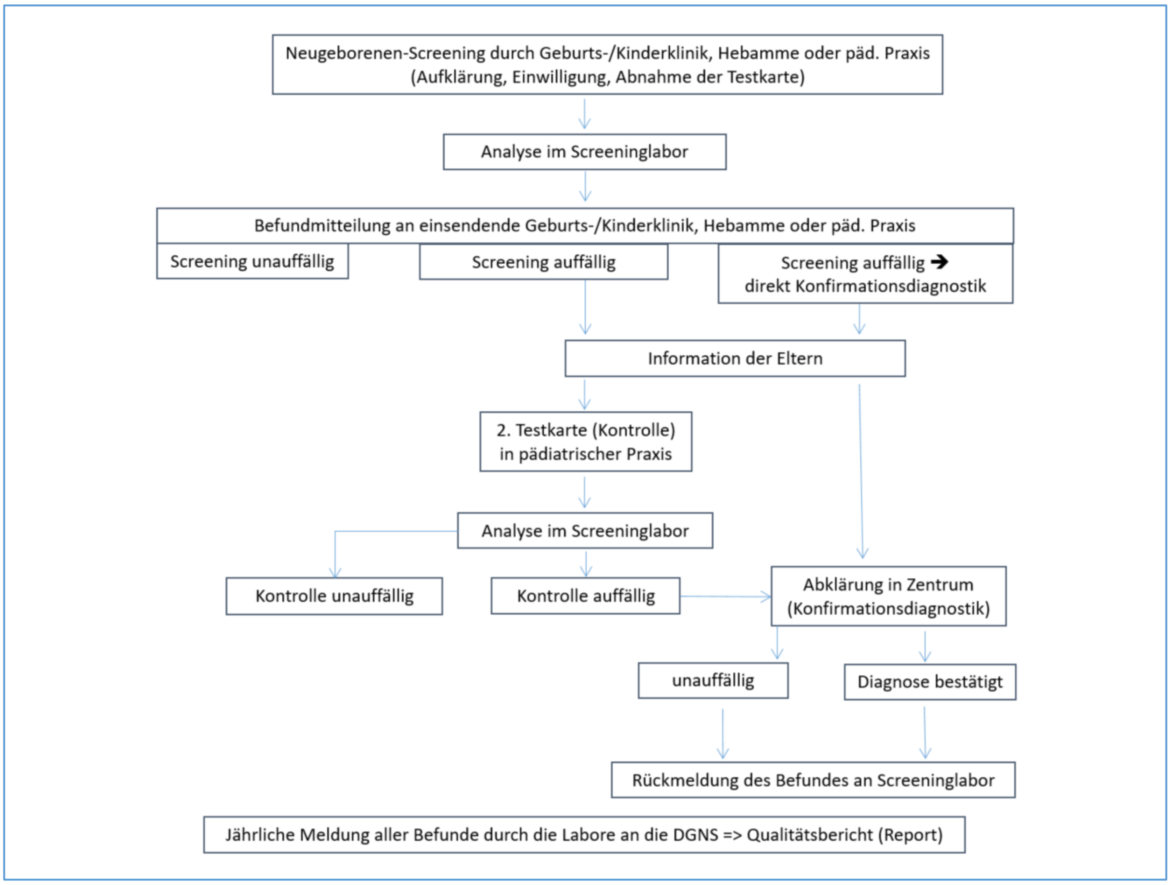

Abbildung 2 Neugeborenen-Screening in Deutschland

Screeningbefunde sollen nach der Kinder-Richtlinie [23] generell nur dem Einsender d.h. meist dem Arzt oder der Ärztin der Geburtsklinik mitgeteilt werden. Ist der Befund auffällig (positiv), so hat der Einsender die Eltern „unverzüglich“ zu informieren und ihnen Kontaktmöglichkeiten (insbesondere Telefonnummern) zu den nächsterreichbaren spezialisierten Zentren mitzuteilen, damit die Eltern dort einen Termin für die weitere Abklärung (Konfirmationsdiagnostik) vereinbaren können. Auch bei grenzwertig positivem Befund oder einer anderweitig notwendigen Wiederholung des Screenings hat der Einsender die Eltern hierüber zu benachrichtigen.

Während die Durchführung des ENS in der Kinder-Richtlinie detailliert geregelt ist, fehlen derzeit wichtige Aspekte der Qualitätssicherung im Screening-Prozess. So finden sich keine Regelungen zum Tracking, d.h. zur Sicherstellung, dass das ENS allen Neugeborenen angeboten wird und auffällige Befunde abgeklärt werden. Dies erfolgt derzeit zuverlässig nur in Bundesländern, in denen ein unabhängiges Trackingzentrum eingebunden ist (Bayern) oder alle Testkarten in ein einziges Screeninglabor eingesandt und mit den Geburtenmeldungen abgeglichen werden.

Insgesamt wird das ENS in Deutschland seit 2005 erfolgreich umgesetzt. Dies zeigen die Qualitätsberichte der Deutschen Gesellschaft für Neugeborenen-Screening (DGNS-Reporte [25]) sowie eine Gesamtübersicht über das Screening der Jahre 2006-2018 [26]. Grundsätzlich müssen Prozesse und Verfahren wie das ENS in einem ständigen Verfahren fortlaufend überprüft und im Sinne eines „Public Health Action Cycle“ angepasst werden [27, 28]. Kernelemente dieses Zyklus sind:
1) die fortlaufende Analyse von Problemlagen, 2) die Entwicklung von Strategien (Maßnahmen) zur Bewältigung der erkannten Probleme 3) die Umsetzung der Bewältigungs-Strategien 4) die Überprüfung (Evaluation) der Wirksamkeit der eingeführten Maßnahmen (s. auch Abbildung 14).

Dies ist jedoch bislang weder für das ENS noch für das CF-Screening implementiert. Aus den in den Publikationen [25, 26] beschriebenen Schwachstellen und Verbesserungspotentialen wurden bislang so gut wie keine Konsequenzen gezogen. Dies wäre insbesondere nach der Einführung neuer Zielkrankheiten notwendig. Zudem wachsen die Anforderungen an die Infrastruktur und Prozesse des ENS mit der Aufnahme jeder neuen Krankheit.

Ziel dieser Arbeit ist eine Erarbeitung von Vorschlägen für die Weiterentwicklung der Strukturen und Prozesse des ENS, um auch bei der zukünftigen Einführung neuer Zielkrankheiten ein hohes Qualitätsniveau des gesamten Screening-Prozesses zu gewährleisten und dieses nachhaltig zu sichern. Die Strukturen und Prozesse des ENS sollen daher auf wissenschaftliche Aktualität, Effektivität, praktische Umsetzbarkeit und Weiterentwicklungsbedarf geprüft werden.

2 Methoden und Datengrundlage

Zunächst sollte als Grundlage für die Konzeptentwicklung der Status quo bei der Umsetzung des ENS in Deutschland ermittelt, Best-Practice-Modelle in anderen Ländern gesucht und der aktuelle wissenschaftliche Stand von Neugeborenen-Screening-Programmen recherchiert werden. Hierzu wurden in Arbeitspaket 1 die im Rahmen der Qualitätsberichterstellung der DGNS erhobenen Daten von 2006 bis 2019 vertieft analysiert, alle Geburtskliniken in Deutschland mittels eines Online-Tools und Vertreter*innen der Screeninglabore, Geburtskliniken, Behandlungszentren, pädiatrischen Praxen sowie Hebammen in strukturierten Interviews bzw. im Rahmen von Fokusgruppen-diskussionen befragt. Arbeitspaket 2 bestand in einer systematischen Literaturrecherche und der Ermittlung von Best-Practice-Modellen. Anhand der strukturierten Ergebnisse wurden die Schwerpunkte für die in Arbeitspaket 3 ausgearbeiteten Konzeptvorschläge identifiziert.

2.1 Arbeitspaket 1: Analyse der aktuellen Strukturen und Prozesse des ENS

Ziel von Arbeitspaket 1 war die Erfassung und Exploration von Stärken und Schwächen des ENS in Deutschland, insbesondere im Hinblick auf die zusätzlichen Anforderungen an die Infrastruktur und Screening-Prozesse durch die bereits neu etablierten Screeningverfahren und die erwarteten Herausforderungen bei der Einführung weiterer Zielkrankheiten. Mögliche Verbesserungspotentiale in Prozessen und Strukturen sollten exploriert und Auswirkungen und Effekte von strukturellen Unterschieden erfasst werden.

2.1.1 Vertiefte Analyse der für die Qualitätsberichte der DGNS erhobenen Daten von 2006 bis 2019

In dem seit 2004 jährlich erstellten Nationalen Screeningreport Deutschland (DGNS-Report) werden die von den Screeninglaboren übermittelten kumulativen Daten für jeweils ein Jahr ausgewertet und deskriptiv nach Screeninglabor und Zielkrankheiten dargestellt [25]. Für die Konzeptentwicklung wurden Qualitätsparameter des Screening-Prozesses wie z.B. Recall-Rate (Rate auffälliger Befunde), Loss-to-follow-up-Rate (Rate nicht abgeklärter Befunde), Prävalenzen und Prozesszeiten in Längs- und Querschnittsanalysen über den gesamten Zeitraum von 2006-2019 und für die einzelnen Labore analysiert (SPSS® IBM Version 25.0).

Für den Zeitraum von 2006 bis 2019 (Abbildung 3) liegen für Deutschland Screeningdaten für 10.022.333 Neugeborene vor (Geburten N= 9.995.717). Bei 7.540 Kindern wurde die Verdachts-diagnose in der Konfirmationsdiagnostik bestätigt, bei 807 Kindern ist unklar, ob die Konfirmations-diagnostik durchgeführt wurde. Hinzu kommen 455 Kinder mit einer bestätigten CF, die nicht in Abbildung 3 berücksichtigt sind. Eine oder mehrere Folgeuntersuchungen waren bei 307.588 Neugeborenen erforderlich und wurden bei 246.253 Kindern (80,06 %) durchgeführt. Eine Zusammenfassung der vertieften Datenanalysen findet sich im Anhang 1.

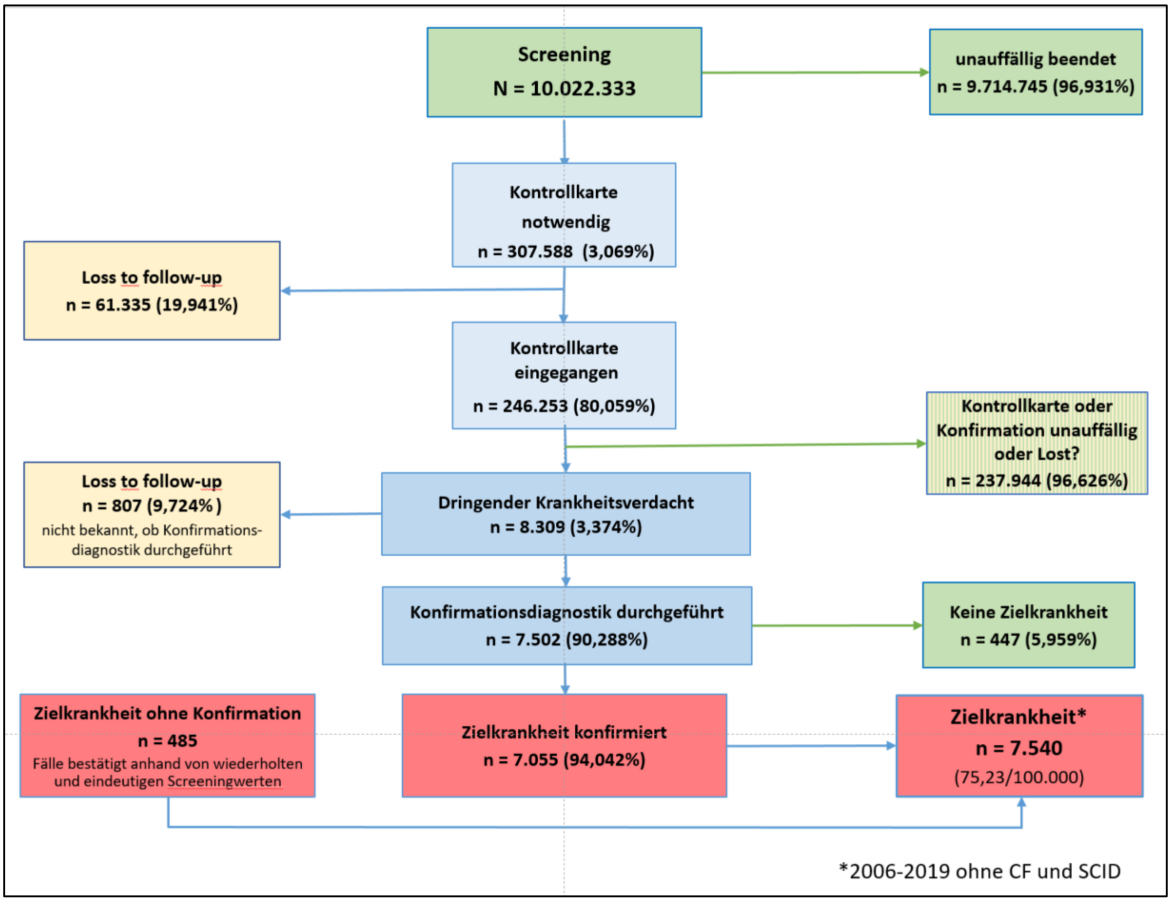

Abbildung 3 Daten zum Screening 2006-2019

2.1.2 Online-Befragungen von Vertreter*innen der Geburtsabteilungen

Anfang April 2021 wurden Vertreter*innen der insgesamt 650 Geburtskliniken in Deutschland mit einem Online-Tool LimeSurve anonym befragt. Dazu wurden die Geburtskliniken per E-Mail um Teilnahme gebeten und erhielten einen Zugangslink zur Befragung. Zuvor wurden die Teilnehmer*innen zum Datenschutz aufgeklärt und eine Einwilligung wurde eingeholt. Mit automatisierter E-Mail wurde an die Befragung erinnert. Im Vorfeld hatte die Ethikkommission der Medizinischen Fakultät der LMU München ein ethisch-rechtliches Unbedenklichkeitsvotum zur Durchführung der Befragung erteilt (Projekt-Nr.: 21-0240 KB).

Fragen zu folgenden Themen wurden gestellt: (1) Basisangaben zu Kliniken und Teilnehmer*innen, (2) Aufklärung der Eltern über das Neugeborenen-Screening (präanalytischer Prozess), (3) Durchführung des Screenings (Koordination) und (4) Befundmitteilung (postanalytischer Prozess). Zwei offene Fragen am Ende des Fragebogens gaben den Teilnehmer*innen die Möglichkeit, weitere wichtige Aspekte im Zusammenhang mit dem ENS zu nennen sowie Wünsche und Ideen bei einer

möglichen Anpassung des Screening-Prozesses aufzuführen. Hier wurde auch die Bereitschaft zur Teilnahme an strukturierten Einzelinterviews (s. 2.1.3) erfragt.

Für die Analysen konnten 194 Fragebögen berücksichtigt werden. Dies entspricht einer Teilnahmerate von 29,9%. Die Verteilung der teilnehmenden Geburtskliniken unterscheidet sich in Bezug auf Bundesland, Geburtenzahl und Versorgungslevel nicht von der der nicht-teilnehmen Geburtskliniken [59,60]. Die Auswertungen erfolgten vornehmlich deskriptiv in SPSS®IBM (Version 25.0). Eine Zusammenfassung der Ergebnisse der Befragung findet sich im Anhang 2.

2.1.3 Strukturierte Einzelinterviews und Gruppendiskussionen

Im Zeitraum von Juli bis Dezember 2021 wurden semistrukturierte Einzelinterviews und Gruppendiskussionen an Hand von Interviewleitfäden geführt. Diese waren auf Basis der Datenanalyse, der vorherigen Befragungen und erster Ergebnisse der Literaturrecherche erarbeitet worden. Die Interviewpartner*innen aus den Geburtskliniken wurden nach Region, Versorgungslevel und Profession unter den zur Teilnahme bereiten Personen aus der Online-Befragung ausgewählt, um regionale und strukturelle Unterschiede im Screening-Prozess zu berücksichtigen. Weitere Hebammen und Pädiater*innen waren als Vertreter*innen der Berufsverbände als Interviewpartner*innen benannt worden.

In den strukturierten Einzelinterviews wurden vier Pädiater*innen, z.T. Neonatolog*innen, und eine Pflegefachkraft aus Geburtskliniken der Versorgungsstufen I bis IV sowie fünf Hebammen und fünf niedergelassene Pädiater*innen befragt. Die Interviewpartner*innen kamen aus Baden-Württemberg, Bayern, Bremen, Niedersachsen, Nordrhein-Westfalen, Rheinland-Pfalz, dem Saarland, Sachsen, Schleswig-Holstein und Thüringen. Niedergelassene Pädiater*innen führen allgemein wenige Neugeborenen-Screening-Untersuchungen im Jahr durch, da diese in der Regel -in der Geburtsklinik erfolgen. Die Interviewteilnehmer*innen schätzten die Anzahl der Screening-Untersuchungen in ihren Praxen auf fünf bis zwanzig pro Jahr.

In weiteren strukturierten Einzelinterviews wurden auch Mitarbeiter*innen aller elf deutschen Screeninglabore befragt. Hier lag der Schwerpunkt der Interviews auf der Analytik und Postanalytik, aber auch auf neuen Herausforderungen bei der Koordination und Dokumentation des Screenings durch zusätzliche Zielkrankheiten. Eine Zusammenfassung aller Einzelinterviews findet sich im Anhang 3. Anschließend wurden im September 2021 die Ergebnisse der Einzelinterviews in einer moderierten Gruppendiskussion mit 16 Mitarbeiter*innen aller Labore zunächst präsentiert und anschließend diskutiert (s. Anhang 4). Des Weiteren war ursprünglich geplant landesweit tätige zentrale Screening-Koordinierungs- und Steuerungs-Zentren zu interviewen, um die Herausforderungen und Möglichkeiten des gesamten Screening-Prozesses zu erfassen. Da derzeit nur zwei Screeningzentren in Deutschland etabliert sind (Bayern und Berlin) und beide Mitglieder der Bewerbergemeinschaft sind, waren diese Interviews hinfällig.

In den Interviews und der Datenanalyse waren Herausforderungen im postanalytischen Prozess sowohl bei der Bereitstellung der notwendigen Infrastruktur für eine zeitnahe Konfirmationsdiagnostik, der Mitteilung des auffälligen Screeningbefundes an die Eltern, der Konfirmationsdiagnostik selbst als auch bei der Rückmeldung von Befunden an das Screeninglabor bzw. -zentrum sichtbar geworden. Um dies und mögliche weitere Schwierigkeiten zu untersuchen, waren zunächst zehn Einzelinterviews mit Expert*innen aus unterschiedlichen Behandlungszentren geplant. Im Laufe des Projektes erschien den Projektpartner*innen eine Fokusgruppendiskussion mit den Expert*innen

geeigneter, um möglichst konstruktive Ideen für die Weiterentwicklung der postanalytischen Prozesse und Strukturen des ENS zu generieren. Für die Fokusgruppe wurden insgesamt 14 potentielle Teilnehmer*innen entsprechend der Samplingstrategie der maximum variation nach regionaler Verortung des Zentrums und Fachrichtung kontaktiert (Anzahl der angefragten Expert*innen aus dem Bereich: Stoffwechsel: 4, Endokrinologie: 3, CF: 2, neue Zielkrankheiten: 5). An der Fokusgruppendiskussion im digitalen Format nahmen zehn Expert*innen aus sieben Fachrichtungen und sechs Bundesländern sowie ein Experte aus Österreich teil (Zusammenfassung s. Anhang 5).

Alle Einzelinterviews und Gruppendiskussionen wurden, nach vorheriger Zustimmung der Interviewpartner*innen, unter Anwendung von Interviewleitfäden virtuell über ein gesichertes Videokonferenz-Tool (Zoom) durchgeführt und digital aufgezeichnet. Das digitale Format bot sich aufgrund des begrenzten Zeitrahmens und der Corona-Pandemie an. Die Interviewdauer betrug im Mittel 30 Minuten, bei den Gruppendiskussionen ca. 2 Stunden. Alle Interviews und die Gruppendiskussionen wurden wörtlich transkribiert und die Transkripte inhaltsanalytisch sowohl induktiv als auch deduktiv nach der Methode von Philipp Mayring ausgewertet [29].

2.2 Arbeitspaket 2: Systematische Literaturrecherche und Suche nach Best-Practice-Modellen

2.2.1 Systematische Literaturrecherche

Die systematische Literaturrecherche zum Neugeborenen-Screening (NBS) aus Trockenblut umfasste Publikationen zum aktuellen wissenschaftlichen Stand grundlegender Screening -Aspekte, -Prozesse und -Strukturen sowie zu Best-Practice-Modellen in anderen Ländern, die seit 2011 veröffentlicht wurden. Besonders berücksichtigt wurden dabei die Aspekte Erweiterung von NBS-Programmen, Screening-Ethik und Programm-Organisation, Qualitätsmanagement, Kommunikation, Tracking bzw. Follow-up sowie digitale Entwicklungsmöglichkeiten. Zusätzlich zur Recherche in den Literatur-Datenbanken PubMed und Scopus wurden, wenn nötig unter Verwendung der frei verfügbaren Übersetzungsprogramme google translate und DeepL, Internet-Darstellungen und -Dokumente zu den NBS-Programmen anderer Länder ausgewertet. Im Anschluss fand mit Expert*innen im Bereich NBS ausgewählter Länder ein Fachaustausch per Telefon, E-Mail oder Videokonferenzen statt.

Die Online-Datenbank-Recherche in der Datenbank PubMed wurde ausgehend von dem MeSH-Begriff „Neonatal Screening“ und den Unterkategorien “trends”, “methods”, “ethics”, “organization & administration” und “legislation & jurisprudence” begonnen und mit adaptierten Suchbegriffen weitergeführt. Nachdem diese Suche nur geringe Trefferzahlen erzielt hatte, wurde die Recherche schließlich mit einer allgemeiner gehaltenen Suchstrategie zu „neonatal screening“ oder „newborn screening“ in Titel oder Abstract durchgeführt. Da für eine präzise Suche zu „Neugeborenen-Screenings mit nativem Venen- oder Fersenblut auf Filterpapierkarten“ keine geeigneten einheitlich verwendeten Begriffe identifiziert werden konnten, wurden zur Verringerung des Anteils nicht relevanter Artikel zu anderen Screening-Bereichen (z.B. Hör-, Hüft- oder Pulsoxymetrie-Screening) Treffer mit „hearing“, „hip“ oder „heart“ im Titel ausgeschlossen.

Für die Jahre 2011 – 2021 wurden die folgenden Suchketten verwendet:
PubMed: ((neonatal screening[MeSH Major Topic]) OR (neonatal screening[Title/Abstract]) OR (newborn screening[Title/Abstract])) NOT ((hearing[Title]) OR (heart[Title]) OR (hip[Title]) OR (retinopathy[Title]) OR (biliary[Title])). Scopus: (TITLE-ABS-KEY ("neonatal screening") OR TITLE-ABS-KEY ("newborn screening")) AND NOT TITLE (hearing) AND NOT TITLE (heart) AND NOT TITLE (hip) AND NOT TITLE (retinopathy) AND NOT TITLE (biliary)

Publikationen, die nach Titel und Abstract relevante Inhalte erwarten ließen, wurden in ein Projekt der Literaturverwaltungs-Software „Citavi" eingelesen und Duplikate identifiziert. Keine Berücksichtigung fanden Publikationen zu anderen Screenings als NBS aus Trockenblut und zu rein medizinischen Aspekten einzelner NBS-Zielkrankheiten, da sie für die Konzeptentwicklung nicht relevant schienen. Zusätzlich wurden die im LGL bereits vorhandene NBS-Literatursammlung mit teilweise älteren Publikationen sowie einzelne aus Literaturverzeichnissen zusätzlich identifizierte Titel in das Citavi-Projekt eingepflegt. Auch Webseiten und Internet-Dokumente, insbesondere zu Screening-Prinzipen und -Grundlagen (z.B. WHO, International Society for Neonatal Screening (ISNS)) und zu den Screening-Programmen anderer Länder, wurden aufgenommen. Es folgte eine inhaltliche Kategorisierung und Überprüfung der Relevanz der im Projekt enthaltenen Dokumente. Aus den Volltexten der als relevant bewerteten Dokumente wurden zu den einzelnen Themenbereichen der Analyse wichtige Zitate extrahiert und Zusammenfassungen als Grundlage für die Konzeptentwicklung erstellt.

Der Ablauf der systematischen Literaturrecherche ist in Abbildung 4 grafisch dargestellt. Wichtige Ergebnisse der systematischen Literaturrecherche werden in den folgenden Abschnitten zusammengefasst und sind im Anhang 6 umfassend dargestellt.

Im Rahmen der Literaturrecherche wurden zwei Dokumente identifiziert, auf die aufgrund ihres Leitliniencharakters immer wieder zurückgegriffen wird. Dies ist zum einen ein Dokument, das 2011 – nach Aufforderung durch den Rat der Europäischen Union zu seltenen Krankheiten von einem Expertengremium – als Konsensgutachten „Newborn screening in Europe – Expert Opinion Document" mit auf den Screening-Kriterien basierenden Statements und Empfehlungen zu „best practices" erarbeitet wurde [8]. Dieses Expertengutachten wurde von den Vorständen der Internationalen Gesellschaft für Neugeborenen-Screening (International Society for Neonatal Screening, ISNS) und der Europäischen Gesellschaft für Humangenetik (European Society of Human Genetics, ESHG) gebilligt und steht unter der Rubrik „Guidelines" auf den Internetseiten der ISNS. Eine Zusammenfassung wurde 2013 von Cornel et al. unter dem Titel „A framework to start the debate on neonatal screening policies in the EU: an Expert Opinion Document" veröffentlicht [30]. Die umfassende NBS-Literatur-Recherche bis 2021 ergab, dass die Statements aus diesem Dokument als weiterhin gültig betrachtet werden können. Wegen ihres Leitliniencharakters werden sie im Folgenden jeweils mit dem Zusatz „EU Expert Document" mehrfach zitiert.

Das zweite Dokument ist ein Leitfaden zu Vorsorgeuntersuchungen und Screening mit dem Untertitel „Wirksamkeit erhöhen, Nutzen maximieren und Schaden minimieren", den die Weltgesundheitsorganisation (WHO) im Jahr 2020 veröffentlichte [22]. Dieser beginnt mit den Screening-Kriterien nach Wilson und Jungner [18] und beschreibt die Bedeutung von Programmkoordination, Evaluation und Qualitätssicherungssystemen für alle Komponenten von Screening-Programmen auf Basis der weiterentwickelten Kriterien [16]. Diese verschieben den Schwerpunkt vom „was" (Anforderungen an geeignete Zielkrankheiten) auf das „wie" des Screenings (Anforderungen an Screening-Programme) [17, 31, 32].

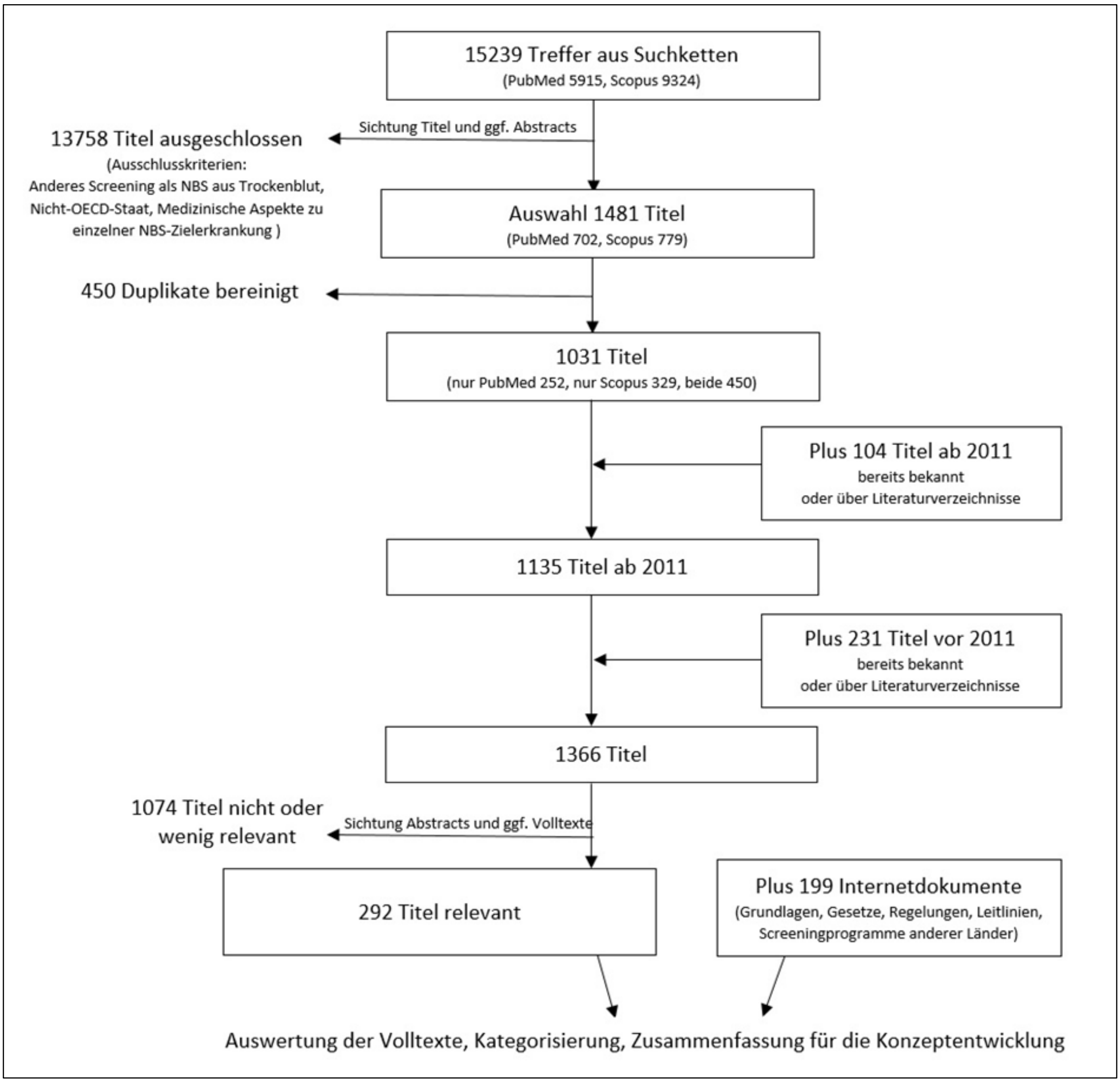

Abbildung 4 Grafische Darstellung der systematischen Literaturrecherche

2.2.2 Länder mit Best-Practice-Modellen

Für die Identifikation von Best-Practice-Modellen des NBS insbesondere unter dem Aspekt, wie in anderen Ländern bei steigender Zahl an Zielkrankheiten ein hohes Qualitätsniveau und eine kontinuierliche Weiterentwicklung des NBS sichergestellt werden, wurde vorrangig nach Ländern mit NBS-Programmen hoher Qualität gesucht, in denen die Screening-Kriterien ähnlich ausgelegt werden wie in Deutschland und in denen das NBS in den letzten Jahren erweitert wurde. Nachdem sich zeigte, dass in Entwicklungs- und Schwellenländern kaum NBS-Strukturen hoher Qualität vorhanden sind [33–35], wurde die detaillierte Länder-Analyse auf die Mitgliedsstaaten der Organisation für wirtschaftliche Zusammenarbeit und Entwicklung (OECD) beschränkt. Sie schienen aufgrund ihrer besseren wirtschaftlichen Situation und Gesundheitsversorgung für Vergleiche mit Deutschland und die Suche nach Best-Practice-Modellen in erweiterten NBS-Programmen am besten geeignet. Grundlegende Informationen wurden für alle OECD-Staaten aus Publikationen und Internet-dokumenten gesammelt sowie für ausgewählte Staaten durch persönliche Kontaktaufnahme ergänzt (Ontario, Niederlande, Norwegen, USA Mayo-Klinik). Da die Fragestellung des Projekts auf die Erweiterung von NBS-Programmen abzielt und sich einige Probleme des deutschen ENS-Systems aus

Bevölkerungs- bzw. Geburtenzahl und Regionalisierung des ENS ergeben, wurde die Recherche zu Ländern mit weniger als 10 Zielkrankheiten des NBS oder weniger als 10 Millionen Einwohnern nur bei Hinweisen auf Beispiele guter Praxis des gesamten Programms oder einzelner Komponenten intensiviert. Zu den NBS-Programmen der Länder ergab die Internet-Recherche über NBS-Webseiten, teilweise ergänzt durch persönliche Kommunikation, insgesamt mehr für das Projekt relevante Informationen als die Recherche in Literaturdatenbanken.

In vielen Ländern fanden sich Best-Practice-Beispiele zu einzelnen Komponenten des Prozesses, jedoch ohne dass sich das gesamte NBS-Programm des jeweiligen Landes als „Best-Practice-Programm“ darstellte. Hohe Standards der Koordination und Qualitätssicherung der NBS-Programme und vieler Einzelkomponenten fanden sich z.B. in skandinavischen Ländern und Neuseeland. Wegen der großen Unterschiede bei der Anzahl der im NBS erfassten Zielkrankheiten zum deutschen Panel, die prinzipielle Unterschiede bei der Auslegung der Screening-Kriterien aufzeigen, wurden trotz teilweise hoher Qualität weder das NBS der USA (30-61 Zielkrankheiten) noch das NBS des UK (9 Zielkrankheiten) insgesamt als „Best-Practice-Programme“ für Deutschland in Betracht gezogen. Als besonders hoch entwickelte Programme mit beeindruckenden Strukturen und Erfolgen bei Koordination und Qualitätsmanagement für nahezu alle Komponenten fielen das NBS in den Niederlanden und in der kanadischen Provinz Ontario auf. Viele der identifizierten Best-Practice-Beispiele aus anderen Ländern bieten interessante Anregungen, sind aber aufgrund von Unterschieden der rechtlichen Rahmenbedingungen und der Strukturen der Gesundheitsversorgung nicht direkt auf Deutschland übertragbar. Ausgewählte Beispiele werden in den folgenden Abschnitten dargestellt und sind im Anhang 7 in der Übersichtstabelle zum NBS in den OECD-Staaten zusammengefasst.

2.3 Arbeitspaket 3: Konzeptentwicklung

Auf Basis der Ergebnisse der Arbeitspakete 1 und 2 wurden durch die Mitglieder der Bewerbergemeinschaft in Zusammenarbeit mit Dr. Ratzel (Recht) und PD Dr. Vieth (Ethik) Optionen für die organisatorische Weiterentwicklung des ENS in einem diskursiv-iterativen Prozess der partizipativen Modellbildung und -anpassung erarbeitet. Bereits während der Phase der Datenerhebung in den Arbeitspaketen 1 und 2 entstand ein erstes Konzept zu Prozessen und Strukturen im ENS, das nach Vorliegen weiterer Ergebnisse immer wieder angepasst wurde (Abbildung 5). Dies erfolgte in regelmäßigen themenorientierten Videokonferenzen mit allen Beteiligten der Bewerbergemeinschaft.

Die Vorschläge in diesem Konzept wurden im Rahmen des rechtlichen (SGB V, GenDG, DSGVO) und ethischen Kontextes auf ihre Umsetzbarkeit in Deutschland geprüft. In einem letzten Schritt wurden die Konzeptideen mit Mitgliedern der Screeningkommission der Deutschen Gesellschaft für Kinder- und Jugendmedizin (DGKJ) diskutiert. Im vorgelegten endgültigen Konzept werden Vorschläge zur Verbesserung der Qualität des ENS in Deutschland detailliert dargelegt und ggf. notwendige rechtliche Anpassungen erläutert.

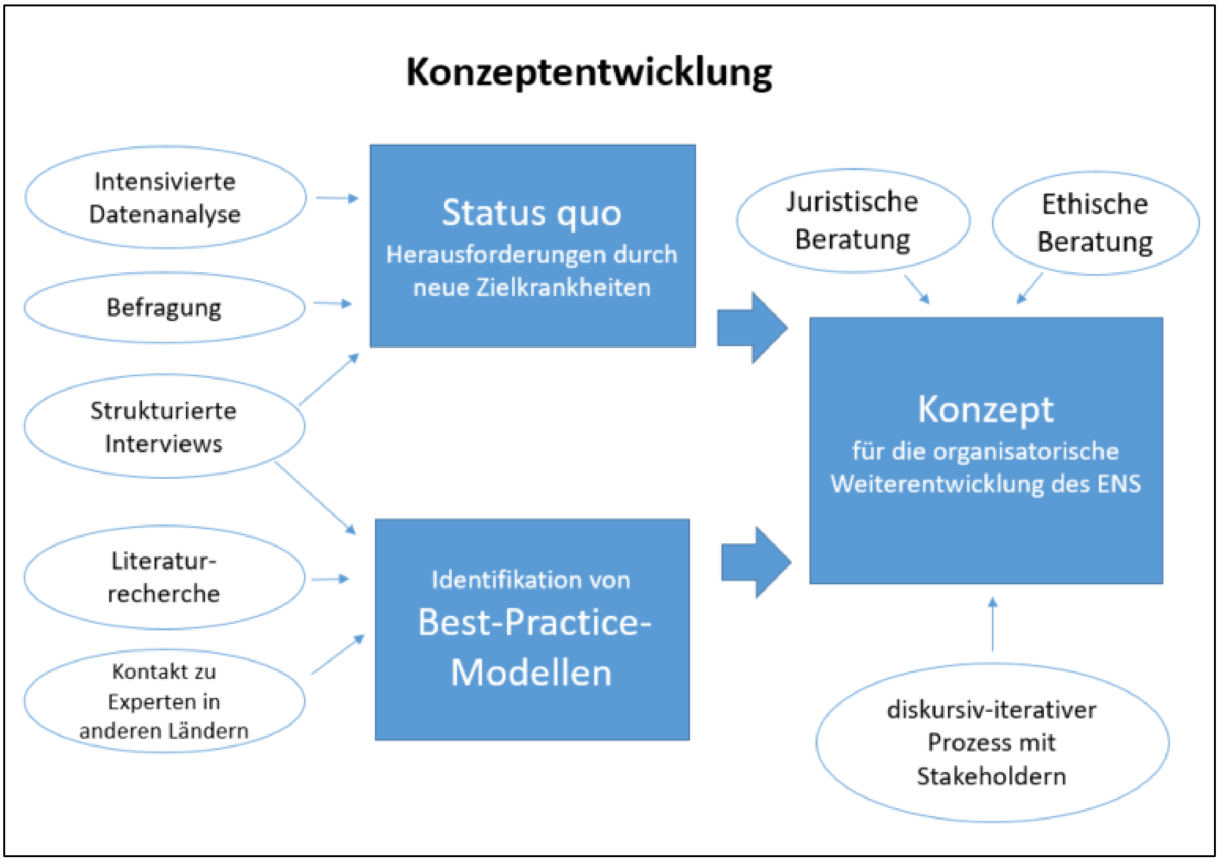

Abbildung 5 Konzeptentwicklung

3 Ergebnisse

In Deutschland kann das „Erweiterte Neugeborenen-Screening“ (ENS) als sehr effektiv betrachtet werden [26]. So enthält das Screeningpanel z.B. nur behandelbare Zielkrankheiten, deren Auswahl sich eng an den Screening-Kriterien von Wilson und Jungner [18] orientiert, die Teilnahmerate ist hoch. Allerdings werden im ENS in Deutschland nicht alle Anforderungen an ein „Screening-Programm“ im Sinne der aktualisierten Screening-Kriterien von Andermann [16] erfüllt. Hier fehlen eine kontinuierliche Qualitätssicherung, die Mechanismen zur Optimierung des Programms und zur Minimierung potenzieller Risiken des Screenings enthält, sowie eine obligate Evaluation inklusive der Vorgaben für die hierfür notwendige Dokumentation. Auch fehlt die Sicherstellung der Finanzierung eines solchen Screening-Programmes über den reinen Screening-Prozess hinaus.

Im Hinblick auf die Sicherstellung einer hohen Qualität des ENS, auch bei einer Erweiterung des Screeningpanels, sowie der Weiterentwicklung des ENS wird in Zukunft auch das genetische Screening eine wichtige Rolle spielen. Unter anderem vor diesem Hintergrund wurden in den Arbeitspaketen 1 und 2 folgende Schwerpunkte identifiziert, für die Konzeptvorschläge auf Basis der Ergebnisse der Arbeitspakete diskutiert werden:

- Eltern-Information, Aufklärung und Einwilligung (Präanalytik)
- Struktur und Qualitätssicherung im Screeninglabor (Analytik)
- Mitteilung auffälliger Befunde, Konfirmationsdiagnostik, Tracking (Postanalytik)
- Digitalisierung (inklusive Screening-ID)
- Zentrale Koordination

3.1 Perspektiven für eine Weiterentwicklung des Neugeborenen-Screenings

3.1.1 Erweiterung der Zielkrankheiten

Seit der bundesweiten Einführung des ENS 2005 sind im Laufe der Jahre mehrere neue Zielkrankheiten in das Screeningpanel aufgenommen worden. Da die Zielkrankheiten des NBS (sehr) seltene Erkrankungen sind, ist die Forderung nach hochgradiger Evidenz als Entscheidungsgrundlage für die Aufnahme neuer Zielkrankheiten in ein Screening-Programm häufig kaum erfüllbar. Um eine möglichst hohe Evidenz zu erreichen, sind Pilotstudien und Register notwendig und wichtig [10, 20, 22, 32]. In Deutschland ist die Beantragung eines Methodenbewertungsverfahrens für mögliche neue Zielerkrankungen beim G-BA in dessen Verfahrensordnung (§4, Kapitel 2 [36]) geregelt. Diese ist sinnvoll, wenn neue Therapiemöglichkeiten zugelassen oder neue für die Analyse eines eindeutigen Markers aus der Trockenblut geeignete Testverfahren entwickelt wurden. Die Bewertung wird dann auf Basis einer Evidenzprüfung durch das Institut für Qualität und Wirtschaftlichkeit im Gesundheitswesen (IQWiG) vorgenommen. Im Anschluss prüft die Gendiagnostik-Kommission nach §16 Gendiagnostikgesetz (GenDG [24]), ob die Screening-Kriterien erfüllt sind, das Anwendungskonzept für die Durchführung der Untersuchung dem allgemein anerkannten Stand der Wissenschaft und Technik entspricht und die Untersuchung in diesem Sinne ethisch vertretbar ist [37]. Insbesondere bei der Beschränkung auf behandelbare Erkrankungen unterscheidet sich die Auslegung der Screening-Kriterien von der in anderen Ländern wie z.B. den USA, wo bis zu 60 teilweise auch nicht therapierbare Krankheiten im Screeningpanel enthalten sind [38]. Länder wie Norwegen oder die Niederlande, in denen ähnliche ethische Überlegungen der Entscheidung zur Einführung neuer Zielkrankheiten wie in Deutschland zugrunde liegen, screenen auf einige wenige zusätzliche sehr seltene Stoffwechselstörungen wie z.B. Methylmalonazidämie, Propionazidämie, HMG-CoA-Lyase-Mangel oder multiplen Acyl-CoA-Dehydrogenase-Mangel [39, 40]. Einige deutsche Screeninglabore führen Pilotprojekte zum Screening auf weitere Zielkrankheiten durch, die die genannten Krankheiten enthalten [9, 41]. Zukünftig könnte auch überlegt werden neue Zielkrankheiten, die über Second-Tier-Verfahren erfasst werden können, methodenbasiert als Gruppe und nicht als Einzelkrankheit zu bewerten. Die Anwendung von mehrstufigen Testverfahren (Second-Tier-Verfahren) mit Bestimmung von Methylmalonsäure, Methylcitrat, 3-OH-Propionsäure und Homocystein würde beispielsweise die Früherkennung von insgesamt 15 Zielkrankheiten erlauben. Hierbei würde u.a. auch ein Vitamin B12 Mangel erfasst werden [42]. Andere diskutierte Erkrankungen wie z.B. Lysosomale Speicherkrankheiten [43, 44] oder die X-Adrenoleukodystrophie [45] sind wegen unklarer Genotyp-Phänotyp-Zuordnungen oder (bisher) fehlender therapeutischer Möglichkeiten nach den Screening-Kriterien (noch) als Zielkrankheiten umstritten.

3.1.1.1 Konzeptvorschläge: Erweiterung der Zielkrankheiten

Es wäre zu diskutieren, ob ein zentral koordiniertes Gremium aus Expert*innen verschiedener Professionen und Fachgesellschaften regelmäßig prüfen sollte, welche Krankheiten für ein Screening geeignet sein könnten, um dem G-BA entsprechende Vorschläge für die Aufnahme eines Methodenbewertungsverfahrens zu machen.

3.1.2 Genetisches Screening

Eine Genomsequenzierung[2] könnte theoretisch bei allen Neugeborenen das Vorhandensein einer Vielzahl weiterer, bereits bekannter pathogenetischer Veränderungen und potentiell behandelbarer Erkrankungen ausschließen bzw. entdecken und damit mehr Kindern die Chance auf Heilung, Prävention, eine adäquate medizinische Therapie oder eine bessere Betreuung und Förderung geben. Als Argumente für die Einführung von Genomsequenzierungen bei allen Neugeborenen werden angeführt, dass sie die Früherkennung eines erweiterten Spektrums genetisch verursachter Erkrankungen und gesundheitlicher Störungen des frühen Kindesalters ermöglichen könnten [46]. Dies gelte insbesondere, wenn eine Gentherapie bei monogenetischen Erkrankungen verfügbar sei, die für eine optimale Wirksamkeit einen frühen Therapiebeginn erfordert [46].

Abgesehen vom potentiellen Nutzen stellen sich jedoch eine Vielzahl technischer, ethischer, sozialer und rechtlicher Fragen in Hinblick auf die Bewertung der Genomsequenzierung als Screeningverfahren bei Neugeborenen [47]. Remec et al. beschreiben in einem Review zum aktuellen Stand des Next Generation Sequencing (NGS) im Neugeborenen-Screening (NBS) folgende Herausforderungen [48]: Der Zeitaufwand für Probenaufbereitung und Auswertung ist für das NBS derzeit zu groß, die Interpretation der riesigen Datenmengen aufwändig und schwierig. Bei Whole Genome Sequencing (WGS) und auch bei Panel-Sequenzierung (möglicherweise werden falsche Zielregionen auf dem Gen ausgewählt) besteht das Risiko falsch negativer Befunde ebenso wie durch unbekannte Pseudogene mit hoher Ähnlichkeit und unbekannte Bereiche im menschlichen Genom. Die Rolle von Epigenetik, nicht codierender Ribonukleinsäure (RNA) und Genexpression als Krankheitsursache ist bei vielen Krankheiten noch nicht wirklich verstanden und einige Krankheiten haben eine sehr variable Penetranz. Auch haben einige Krankheiten keine eindeutige Genotyp-Phänotyp Korrelation (z.B. Fettsäurestörungen, Lysosomale Speicherkrankheiten) und die Detektion von Varianten mit unklarer klinischer Relevanz, unterschiedlicher Klassifikation in verschiedenen Databases und prädiktivem Algorithmus machen eine zuverlässige Interpretation schwierig (z.B. CF). Eine Evaluation einer validen genetischen Prädiktion bei vielen derzeitigen Zielkrankheiten fehlt noch [49]. Aus diesen Gründen kann derzeit ein ENS auf Basis eines primär genetischen Screenings bis auf wenige Ausnahmen (z.B. SMA) noch nicht empfohlen werden [3, 46, 50, 51].

Für metabolische Krankheiten, die mit biochemischen Methoden der Tandem-Massenspektrometrie (MS/MS) gescreent werden, kann Genetik ggf. als Second-Tier Verfahren eingesetzt werden, um die Recall-Rate zu senken [51–54]. Auch für monogenetisch bedingte Erkrankungen, die derzeit nicht mit biochemischen Markern detektiert werden können, wie z.B. SMA, ist ein zusätzliches genetisches Screening sinnvoll [50], sofern die oben genannten Herausforderungen bewältigt werden können.

3.2 Elternaufklärung und Einwilligung

Die sachgerechte Information der Eltern ist ein wichtiger und integraler Bestandteil eines NBS-Programms. Bei den meisten Zielkrankheiten handelt es sich um angeborene, genetisch bedingte Erkrankungen. Die Diagnose einer der Zielkrankheiten ist für die Familien trotzdem meist völlig unerwartet. In den meisten dieser Familien gab es vorher noch nie derartige Erkrankungen und die

[2] Der Begriff „Genomsequenzierung“ steht hier für die verschiedenen Anwendungsmöglichkeiten der Next-Generation Sequenzierungstechnologie, d.h. sowohl für die Analyse des gesamten Exoms oder Genoms als auch den, aus den Rohdatenausgewählten „virtual gene panels“ oder „exome slices“

betroffenen Kinder erscheinen bei der Geburt noch völlig gesund. Ursache ist der autosomal rezessive Erbgang bei den meisten der Zielkrankheiten. Beide Eltern von betroffenen Neugeborenen sind dann sog. heterozygote Anlageträger, die selbst aber nicht krank sind.
Das Risiko, dass das krankmachende Gen bei einem der Kinder solcher Paare zusammenkommt und die Erkrankung auslöst, liegt dann bei 25%. Den Eltern muss folglich vermittelt werden, dass das NBS wichtig ist, auch wenn bei ihren Vorfahren keine der Erkrankungen bekannt sind. Auch müssen die Eltern darüber aufgeklärt werden, dass im NBS Suchtests eingesetzt werden, die keine Diagnose liefern, sondern bei Auffälligkeiten weiter abgeklärt werden müssen. Eine gewisse Rate an falsch positiven Screening-Ergebnissen, die die Eltern beunruhigen können, ist unvermeidbar, wenn möglichst keine betroffenen Kinder übersehen werden sollen.

3.2.1 Ergebnisse der Literaturrecherche

Die Aufklärung der Eltern gilt als wesentlicher Bestandteil des NBS-Prozesses [55], unabhängig davon, ob das Screening verpflichtend ist (wie z.B. in den meisten US-Bundesstaaten, auch Italien und Dänemark) oder, wie in den meisten europäischen Staaten, eine Einwilligung der Eltern erfordert. Die Eltern sollen ausreichende Informationen erhalten, um die Notwendigkeit des Screenings zu verstehen, gleichzeitig aber nicht beunruhigt werden [56]. Befragungen in verschiedenen Ländern ergaben, dass Eltern auf Informationen zum Screening mehr Wert legen als auf Entscheidungsmöglichkeiten bzw. Einwilligung [57, 58]. Einige Autor*innen halten es für vertretbar, Screening auf Zielkrankheiten mit eindeutigem Benefit für das Kind (wie Phenylketonurie (PKU), angeborene Hypothyreose, MCAD-Mangel) verpflichtend bzw. ohne Einwilligung durchzuführen [59–62].

Zunehmend werden gedruckte Materialien als einzige Aufklärungs-Medien kritisch gesehen [57]. Innovative digitale Wege der Informationsvermittlung wie Videos oder Online-Informationsseiten werden als Ersatz oder Ergänzung der klassischen Broschüren empfohlen [59, 63, 64].

- **Optimaler Zeitpunkt der Aufklärung:**
  Als optimal wird die pränatale Aufklärung im letzten Trimenon der Schwangerschaft betrachtet [30, 57, 65–68]. Die postnatale Aufklärung wird als uneffektiv und ungünstig angesehen, denn viele Eltern sind kurz nach der Geburt für diese doch komplexen Informationen nur wenig aufnahmefähig [55, 65].

- **Form der Aufklärung:**
  Das persönliche Gespräch mit einer ärztlichen Person oder einer Hebamme wird von den Eltern bevorzugt, wobei die Hebamme mehrfach als am besten geeignete Person genannt wird [55, 57]. Allerdings unterscheiden sich im internationalen Kontext Ausbildung und Rollenverständnis von Hebammen. Auch die Aufklärung durch weiteres geschultes Fachpersonal, unterstützt durch geeignete Materialien, wird als Möglichkeit gesehen. Eine Gruppenaufklärung mit anschließender individueller Rückfragemöglichkeit wird ebenfalls diskutiert (4. Tätigkeitsbericht der GEKO in Vorbereitung). Insgesamt gilt eine „differenzierte" Aufklärung als beste Variante, bei der die Eltern Basis-Informationen im ersten pränatalen Gespräch und in übersichtlichen Materialien (Broschüre, Video) erhalten und dann je nach Interesse zusätzlich vertiefende Informationen z.B. in einem weiteren Gespräch oder durch Materialien bekommen können [30, 57, 58, 69]. Empfohlen werden Richtlinien zur Eltern-Aufklärung, die evidenzbasierte Best Practice berücksichtigen [55].

- **Informationsmaterialien:**
  Grundlegende und unbedingt erforderliche Inhalte von Informationsmaterialien sind Ziel und Nutzen des NBS, die Beschreibung des Screening-Prozesses, mögliche Ergebnisse und ihre Bedeutung (Wahrscheinlichkeit für das Auftreten eines auffälligen Befundes sowie die daraus folgende Erkrankungswahrscheinlichkeit, Möglichkeit falsch positiver und falsch negativer Befunde) sowie Hinweise auf Zugangswege zu weiterführenden Informationen [8, 30, 58, 70]. Dabei soll die Bedeutung des NBS für das Wohl des Kindes deutlich gemacht werden. Aus ethischer Sicht ist entscheidend, dass der Screening-Prozess sowie das gesamte Programm, bei Bedarf inklusive der hierfür zugrundeliegenden Entscheidungen, für die Eltern transparent ist [68]. Evidenzbasierte Patienteninformationen über das Neugeborenen-Screening müssen der gesamten untersuchten Bevölkerungsgruppe in mehreren Sprachen, kultursensibel und barrierefrei zur Verfügung stehen und auf den Webseiten der für das Screening zuständigen Einrichtungen veröffentlicht werden [8, 22, 30].

3.2.2 Situation in Deutschland

3.2.2.1 Rechtliche Situation

Prinzipiell müssen die Eltern (Sorgeberechtigten) vor der Blutabnahme für das ENS ärztlich aufgeklärt werden und ihr schriftliches Einverständnis erteilen. Aufgrund der Regelungen in der Kinder-Richtlinie (Abs.1 §6 [23]) dürfen in Ausnahmefällen auch Hebammen, die die Geburt verantwortlich geleitet haben, unter der Voraussetzung, dass die Rückfragemöglichkeit an einen Arzt oder eine Ärztin gewährleistet ist, die Aufklärung über das ENS, nicht aber über das Neugeborenen-Screening auf Mukoviszidose (CF-Screening), übernehmen. Daher müssen zwei Einwilligungen eingeholt werden (ENS plus CF-Screening). Regelungen zum Zeitpunkt der Aufklärung oder eine Erklärung zur Wahrscheinlichkeit auffälliger Befunde sind in der Richtlinie nicht enthalten.
In Deutschland unterliegen das ENS und das CF-Screening dem GenDG [24]. Entscheidend ist danach die ärztliche mündliche Aufklärung, die selbstverständlich durch schriftliches oder digitales Informationsmaterial ergänzt werden kann.

3.2.2.2 Online-Befragung von Vertreter*innen der Geburtskliniken

Die Befragung der Geburtskliniken und persönlich mitgeteilte Erfahrungsberichte von Eltern deuten darauf hin, dass das nach der Kinder-Richtlinie und dem GenDG erforderliche ärztliche Aufklärungsgespräch in der Praxis nicht die Regel ist. So gaben in der Online-Befragung (n=194) nur 32,0% der Befragten (62/194) an, regelmäßig ein ärztliches Aufklärungsgespräch zu führen, weitere 11,9 % (23/194) führen ein Aufklärungsgespräch entweder ärztlich <u>oder</u> durch eine Pflegefachkraft bzw. Hebamme, 50% (97/194) geben schriftliches Material mit Möglichkeit zur Rücksprache aus (davon 70% Rücksprachemöglichkeit mit Arzt oder Ärztin, 30% mit einer Pflegefachkraft) und bei 2,1% erfolgt die Aufklärung nur durch Informationsmaterialien. Bei den offenen Fragen wurde wiederholt eine Ergänzung schriftlicher Materialien durch digitale Angebote angeregt. Knapp 70% der Befragten würden Videos oder Internetseiten verwenden, wenn diese angeboten würden, 73,7% der Teilnehmer*innen hielten eine Aufklärung zum ENS bereits in der Schwangerschaft für sinnvoll. 13% der Befragten merkten an, dass der Zeitpunkt für die Aufklärung nach der Geburt und im Krankenhaus unpassend sei. Eine Aufklärung zum Screening vor der Geburt durch Gynäkolog*innen oder Hebammen, beispielsweise in Geburtsvorbereitungskursen, wurden als mögliche Verbesserungen genannt.

3.2.2.3 Einzelinterviews

Auch die interviewten Hebammen und Pädiater*innen hielten die Aufklärung zum Screening vor der Geburt für sinnvoll. Die Hälfte der Interviewteilnehmer*innen aus Geburtskliniken und interviewte Hebammen finden die Elterninformation zu schwierig formuliert und mit zu vielen Fachbegriffen versehen. Niedergelassene Pädiater*innen und Hebammen waren der Auffassung, dass die Eltern das Aufklärungsmaterial zum Screening nicht lesen, sondern auf die mündliche Aufklärung und Einschätzung der aufklärenden Person vertrauen. „Wenn man die Aufklärung zum Screening so machen würde, wie sich der G-BA das vorstellt, dann wäre das ein Riesenaufwand. Aber in der Praxis ist das kein großer Aufwand, weil die Leute wollen wissen ist das sinnvoll oder nicht und nicht über 17… oder 30 Krankheiten … informiert werden“ sagte ein Pädiater. Detaillierte Informationen zu den Krankheiten würden in der Regel erst bei auffälligen Befunden nachgefragt. Die interviewten freiberuflichen Hebammen und ein Pädiater hielten die Aufklärung durch Hebammen mit Rückfrage-möglichkeiten bei einem Arzt oder einer Ärztin für sinnvoll. Diese Forderungen wurden auch in anderen Interviews gestellt.

3.2.3 Ethische Überlegungen

Es ist keine Frage, dass Eltern der Blutentnahme und Verwendung des Blutes ihres Neugeborenen zustimmen müssen. Die Zustimmung zu medizinischen Maßnahmen wird heute zumeist als "informed consent" konzipiert [71–74]. Eltern sind in der Regel keine Mediziner*innen und müssen, um entscheiden zu können, informiert werden. Der Gegenstand der Entscheidung bestimmt dabei die benötigten Informationen. Bisher herrscht die Auffassung vor, dass im Rahmen der aufklärenden Information zum NBS über jede einzelne Zielkrankheit informiert werden muss. Dies hat sich als ungünstig erwiesen, da der Gegenstand der Beratung den Schwerpunkt auf potentielle Krankheiten des neugeborenen Kindes legt. Die Aufklärung kann dabei durchaus beunruhigende Wirkung auf die Eltern haben und die Haltung zu ihrem Kind verändern: Das Kind ist potentiell krank und die Eltern sind möglicherweise veranlasst, nach Symptomen der Krankheit zu suchen, die Gegenstand der Aufklärung waren. Beides ist unnötig. Unter ethischen Gesichtspunkten ist der Anlass des Screenings nicht im neugeborenen Individuum zu sehen (es gibt keine Indikation), sondern wie bei Public-Health-Maßnahmen üblich in der Population. Der Gegenstand der Zustimmung ist also primär die methodische Notwendigkeit der Blutentnahme am Individuum für das Screening der Population. Hierauf sollten sich die Informationen der Aufklärung beziehen. Es sollte insgesamt über das Screening-Programm als solches (Ziele, Methoden, Prozesse) informiert werden. Durch die damit geschaffene Transparenz wäre dann der Bedingung eines informed consent Genüge getan, ohne dass die Eltern sich zu diesem Zeitpunkt mit Informationen zu den Krankheitsbildern der seltenen und komplexen Zielkrankheiten auseinandersetzen müssen. Der Sinn der Aufklärung besteht aus ethischer Perspektive darin, Vertrauen durch Verfahrenstransparenz zu bewirken. Auch aus rechtlicher Sicht ist die Auflistung aller Zielerkrankung in der Elternbroschüre zum NBS nicht erforderlich. Der Bereich der spezifischen elterlichen Verantwortung für die Entscheidungen über die Fragen der Gesundheit ihres Neugeborenen beginnt erst, wenn sich aus dem auf die Population bezogenen Screening durch ein auffälliges Screeningergebnis Rückschlüsse auf das Neugeborene ergeben. Dann erst werden die krankheitsspezifischen Informationen individuell relevant: für die Gesundheit des Kindes und die Entscheidung der Eltern. Erst jetzt sind Belastungen der jungen Familien moralisch vertretbar, die aus Informationen zu spezifischen Krankheitsbildern resultieren. Diese Überlegungen müssen bei der Aufklärung zum NBS und bei der Gestaltung der Informationsmaterialien berücksichtigt und auch im Kontext der einschlägigen rechtlichen Normen in Deutschland betrachtet werden.

3.2.4 Best-Practice-Modelle

Ein von medizinischem Fachpersonal geführtes pränatales Informationsgespräch mit vorgegebenen Inhalten im letzten Schwangerschaftsdrittel ist z.B. in Neuseeland [75], den Niederlanden [76], Norwegen [77] und England [78] Teil des NBS-Prozesses. Zusätzlich erhalten die Eltern schriftliche Informationsmaterialien und werden auf Webseiten und Videos hingewiesen. Die elterliche Einwilligungserklärung erfolgt in England ebenfalls pränatal, in den anderen genannten Ländern dann postnatal vor Durchführung des NBS, nach erneutem schriftlichem und mündlichem Informationsangebot und der Beantwortung ggf. offener Fragen. In vielen Ländern gibt es Webseiten mit Informationen zum NBS für die Öffentlichkeit und für (werdende) Eltern. Als besonders gutes Beispiel, mit gut verständlichen aufs Wesentliche fokussierten anschaulichen Informationen und Vertiefungsmöglichkeiten, fielen die Webseiten des NBS-Programms in der kanadischen Provinz Ontario auf [79]. Die Elternbroschüren zum NBS sind in den meisten Ländern auf den Webseiten zum Download verfügbar, teilweise auch in verschiedenen Sprachen (z.B. Niederlande 5 Sprachen [80], England 13 Sprachen [81], Victoria/Australien [82] und Finnland je 14 Sprachen [83], kanadische Provinzen 9-30 Sprachen [84, 85]). Schweden stellt die wichtigsten Inhalte in einem gut übersetzbaren Informationstext zur Verfügung, der durch unkompliziertes Anklicken direkt in alle über „google translate“ verfügbaren Sprachen (> 100) übersetzt werden kann [86]. In den Elternbroschüren werden überwiegend die Zielkrankheiten des NBS nur zusammenfassend oder exemplarisch genannt, mit Betonung des Nutzens der Früherkennung und früher Behandlung; für weitere Informationen zu den einzelnen Zielkrankheiten wird auf die Webseiten verwiesen. Auch in Leichter Sprache gibt es in England die Elternbroschüre [87], auf den niederländischen Webseiten eine das NBS erklärende Bildergeschichte in 7 Sprachen [88]. Häufig finden sich auf den Webseiten auch Eltern-Videos zum NBS, so z.B. in Australien [82, 89, 90], Belgien [91], Costa Rica [92], Neuseeland [93], Niederlande [94], Norwegen [95], Schweden (mit Untertiteln in 10 Sprachen) [96], Ontario (Video in 30 Sprachen) [97], England [98] und den USA [99].

3.2.5 Zusammenfassung

- Der optimale Zeitpunkt der Aufklärung zum NBS liegt im letzten Trimenon der Schwangerschaft.
- Ein persönliches Gespräch mit Hebamme, Arzt oder Ärztin oder anderem geschultem Fachpersonal, unterstützt durch geeignete Materialien, nicht nur in deutscher Sprache, wird als beste Aufklärung angesehen.
- Optimal ist eine differenzierte Aufklärung mit einer Basis-Information in einem ersten Gespräch und mit gut verständlichen und barrierefreien Materialien (Broschüre, Video), die je nach Interesse der Eltern durch weitere Informationen z.B. in einem erneuten Gespräch oder durch zusätzliche Materialien vertieft werden kann.
- Zentrale Inhalte der Aufklärung sind Ziel, Nutzen und Abläufe des NBS.
- Informationen zu den einzelnen Krankheitsbildern im Rahmen der Aufklärung sind ethisch problematisch und rechtlich nicht erforderlich. Die einzelnen Zielkrankheiten sollten daher nicht Bestandteil der Basis-Elterninformation sein.
- Allgemein verständliche und vertiefende Informationen zum NBS-Programm sollten auf Webseiten verfügbar sein.
- Wünschenswert sind Richtlinien zur Eltern-Aufklärung, die evidenzbasierte Best Practice berücksichtigen.

3.2.6 Konzeptvorschläge: Aufklärung

Die werdenden Eltern sollten pränatal eine Aufklärung über das Neugeborenen-Screening erhalten. Dies könnte z.B. im Rahmen der Schwangerenvorsorge oder auch als Gruppenaufklärung in Geburtsvorbereitungskursen stattfinden. Ebenso wäre eine vorgeburtliche Beratung in einer pädiatrischen Praxis denkbar, wie sie im Rahmen eines Projekts als „U0“ in Bayern durchgeführt wird [100]. Die Aufklärung und Information könnte ggf. durch (geschulte) Hebammen erfolgen, wenn die Rückfragemöglichkeit an eine ärztliche Person gegeben ist. Aus praktischen Gründen könnte die schriftliche Einwilligung, die zusammen für das ENS und das CF-Screening erteilt werden sollte, weiterhin postnatal dokumentiert werden, wobei auch dann noch offene Fragen geklärt werden könnten.

Neben der mündlichen Aufklärung sollten den werdenden Eltern auch eine schriftliche oder digitale Elterninformation angeboten werden. Wichtige Inhalte der Elterninformation sind Ziele und Nutzen des Screenings für das Kind sowie die Beschreibung des Screening-Prozesses (-Programms). Auch mögliche Ergebnisse und ihre Bedeutung, die Wahrscheinlichkeit für das Auftreten eines auffälligen Befundes und die daraus folgende Erkrankungswahrscheinlichkeit sowie die Möglichkeit falsch negativer und falsch positiver Befunde sollten enthalten sein, nicht aber Informationen zu jeder einzelnen Zielkrankheit. Denkbar wäre eine kurze Elterninformation, die auch in den gängigen Fremdsprachen sowie Leichter Sprache zur Verfügung steht und zusätzlich Hinweise auf Zugangswege zu weiterführenden Informationen wie e-Material oder Videos enthält. Auch eine Beschreibung der einzelnen Zielkrankheiten kann über einen Link online einsehbar sein.

Die zentrale Bereitstellung von Leitfäden und Schulungen für aufklärendes Fachpersonal sowie eine Vergütung der Informations- bzw. Aufklärungsgespräche auch pränatal könnten helfen die Qualität der Aufklärung weiter zu verbessern.

3.3 Struktur und Qualitätssicherung im Screeninglabor

3.3.1 Ergebnisse der Literaturrecherche

Der Erfolg von NBS-Programmen wird maßgeblich von der Qualität der Screeninglabore und ihrer Verfahren geprägt [101]. Es herrscht Konsens, dass Screeninglabore qualifiziert und zertifiziert sein sollen und sowohl interne als auch externe Qualitätssicherung in entsprechenden Strukturen erforderlich sind [30, 102, 103]. Regelmäßige Schulungen stellen die Kompetenz des Personals und damit eine möglichst hohe analytische Qualität sicher [22, 102–104].

In den meisten Ländern werden keine gesetzlichen oder staatlichen Vorgaben für die anzuwendenden Analysemethoden oder die zu verwendenden Test-Kits gemacht. Allerdings besteht in Ländern mit nur einem Screeninglabor (z.B. die skandinavischen Staaten, Neuseeland, Portugal, Österreich, Schweiz) keine Veranlassung für einheitliche Vorgaben der Screeningmethoden. Für Länder mit mehreren Screeninglaboren fanden sich meist allenfalls Vorgaben für die zu screenenden Parameter ohne explizite Vorgaben zu den anzuwendenden Analysemethoden (z.B. Italien, Spanien). Hinweise auf Beschränkungen bei der Methodenauswahl ergaben sich nur für die Niederlande und England: im niederländischen NBS-Programm arbeiten die 5 Screeninglabore mit einheitlichen Geräten und Methoden [persönliche Mitteilung]; in England sind die möglichen Methoden in „Laboratory Guides“ beschränkt [105, 106].

Wichtige Qualitätsparameter in einem Screeninglabor sind valide und reliable Analysen der festgelegten Screening-Parameter. Entscheidend für den Screening-Prozess sind aber nicht nur die Qualität der Analysen, sondern auch eine valide Befundung der Testergebnisse als unauffällig, grenzwertig oder auffällig. Grenzwerte (Cut-offs) müssen für jeden Parameter so festgelegt werden, dass möglichst alle Betroffenen erkannt werden können (hohe Sensitivität). Trotzdem soll gleichzeitig der Anteil falsch-positiver Befunde möglichst geringgehalten werden. Dies kann im NBS bei einigen Krankheiten durch multiple Marker, biochemische oder molekulargenetische zwei- oder auch dreistufige Testverfahren [10, 32, 42, 54, 107, 108] (Abbildung 6) und regelmäßige Überprüfung und ggf. Anpassung der Cut-offs [109] erreicht werden.

In einer Videokonferenz mit Kolleg*innen aus dem norwegischen Screeningzentrum wurden uns die Möglichkeiten aber auch großen Herausforderungen durch molekulargenetische Second-Tier-Teststrategien berichtet.

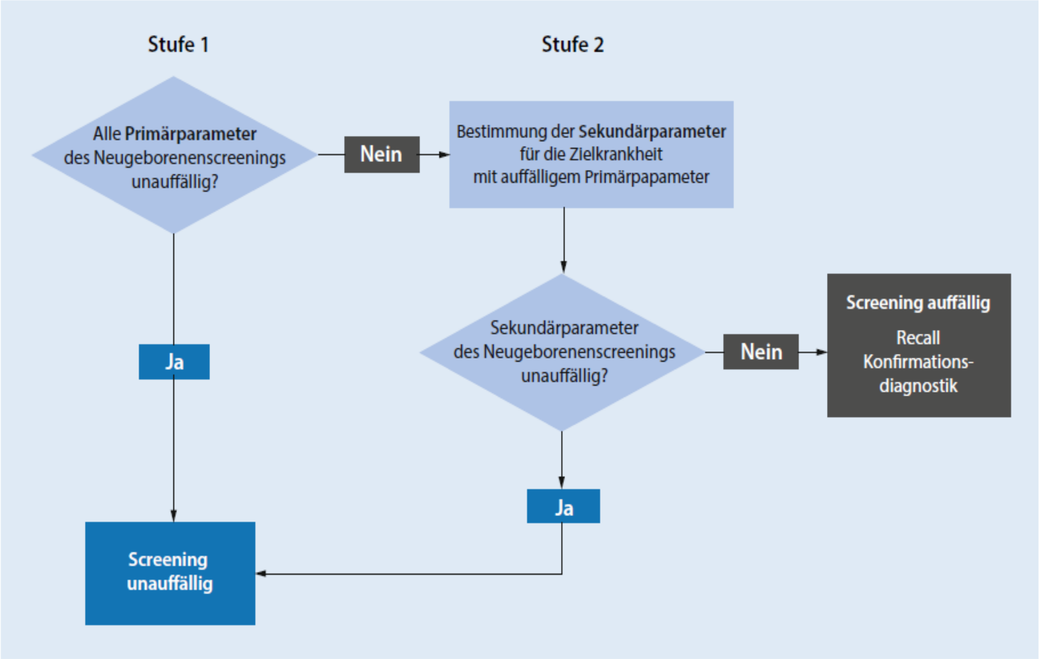

Abbildung 6 Mehrstufige Testverfahren (Second-Tier-Strategie) [42]

In den letzten Jahren werden weltweit zunehmend auch post-analytische multivariate digitale Interpretationswerkzeuge genutzt wie z.B. „CLIR“ (vormals R4S) [52, 110–118] oder ähnliche Verfahren [114, 119]. Collaborative Laboratory Integrated Reports (CLIR 2.24; [120]) ist eine Webanwendung, die eine interaktive Datenbank mit Laborergebnissen verschiedenster Standorte weltweit verwaltet. Die multivariate Mustererkennungssoftware dient dazu, die postanalytische Interpretation von Screeningergebnissen zu verbessern, indem die Differenzialdiagnose zwischen richtig- und falsch-positiven Befunden optimiert wird. Basierend auf retrospektiven Analysen von „Big Data“ aus weltweiten Meldungen ermöglicht die CLIR-Software die multivariate Anpassung der Patientenergebnisse mit den Kovariaten Gestationsalter, Geburtsgewicht, Alter bei Blutentnahme und Analytik im eigenen Labor. Die traditionellen Cut-off-Werte werden durch kontinuierliche (gleitende) kovariaten-adjustierte Perzentilen ersetzt. Für alle auffälligen Ergebnisse wird ein Wahrscheinlichkeits-Score für die Zielkrankheit kalkuliert. Dies ermöglicht es den Nutzern, die Ergebnisse für jeden Patienten und jede Patientin individuell zu interpretieren und echte pathologische Befunde besser zu erkennen. Ziel ist ein „Präzisions-Screening“ mit möglichst geringen Falsch-Positiv-Raten. Dies scheint insbesondere bei den sehr seltenen Krankheiten oder der

Befundung bei Frühgeborenen sinnvoll. CLIR wird von der Mayo Klinik in den USA auf einem sicheren Server zur Verfügung gestellt und ist weltweit frei zugänglich. Bevor jedoch der Zugang freigeschaltet wird, müssen zunächst anonymisierte Daten und Profile von mindestens 10.000 Referenzfällen in einem definierten Format übermittelt werden. Im Jahr 2019 wurden die CLIR-Tools weltweit von 456 Nutzer*innen aus 34 Ländern 238 Millionen Mal genutzt, mehr als 650.000 Mal pro Tag [121]. Auch in einem persönlichen Interview wurden die Vorzüge und Möglichkeiten des CLIR-Verfahrens vorgestellt und berichtet, dass CLIR demnächst von der FDA zertifiziert werden soll. Die Anwendung einer solchen spezifischen Software unterliegt in der EU den europäischen Regularien für Medizinprodukte. Die neue europäische Verordnung über Medizinprodukte, (EU) 2017/745 (MDR) ersetzt dabei die bisherige Richtlinien über Medizinprodukte (93/42/EWG, MDD) mit einer Übergangsfrist bis Mai 2025. Derzeit entsprechen die amerikanischen Datenschutzregelungen nicht der europäischen Datenschutzgrundverordnung (DSGVO), was die Nutzungsmöglichkeit von CLIR in Deutschland einschränkt.

3.3.2 Situation in Deutschland

3.3.2.1 Rechtliche Situation

Die Kinder-Richtlinie legt die notwendigen laborärztlichen Qualifikationen für die Durchführung des ENS sowie die Rahmenbedingungen für das Screeninglabor fest (§§23-26) [23]. Nach §25 (2) Kinder-Richtlinie muss das Labor für die durchzuführenden Untersuchungen mit den entsprechenden technischen Einrichtungen ausgestattet sein und über qualifiziertes Personal verfügen. Diese organisatorisch-apparativen Voraussetzungen gelten mit einer Akkreditierung für medizinische Laborleistungen durch die Deutsche Akkreditierungsstelle GmbH (DAkkS GmbH) als belegt. Derzeit sind die Analysemethoden für alle Zielkrankheiten in der Richtlinie festgelegt und wurden seit 2005 für die metabolischen und endokrinologischen Erkrankungen nicht aktualisiert. Mittlerweile publizierte Verfahren, durch die eine Reduktion der falsch positiven Befunde möglich wird (z.B. Second-Tier-Analysen, s. 3.3.1), sind nicht vorgesehen. Auch fehlen Tools für die postanalytische Beurteilung eines Wertes als auffällig oder unauffällig ebenso wie Qualitätsparameter oder vorgegebene Referenzbereiche. Zwischen den elf Screeninglaboren in Deutschland ist kein geregelter Austausch (weder der Daten noch der Analytik) vorgesehen. Auf freiwilliger Basis diskutieren die Labore die Analytik regelmäßig im Rahmen von Laborworkshops der DGNS.

3.3.2.2 Auswertung der DGNS-Daten 2006 bis 2019

Recall-Raten
Die Höhe der Recall-Rate, d.h. der Anteil der auffälligen Screeningbefunde, ist ein entscheidender Qualitätsparameter, der insgesamt im deutschen ENS niedrig ist und von 2006 bis 2015 von 0,90 % auf 0,45 % halbiert werden konnte. Mit der Einführung jeder neuen Zielkrankheit (CF 2016, Tyrosinämie 2018, Schwere Immundefekte SCID 2019) steigt die Rate allerdings wieder an, da auffällige Befunde für jede dieser Krankheiten hinzukommen (Abbildung 7).

Möglich war die Senkung der Recall-Rate insbesondere durch Einführung von mehrstufigen („Second-Tier“) Test-Verfahren in einigen Laboren (s. Pfeile in Abbildung 8) für das Screening auf Adrenogenitales Syndrom (AGS) mit LC-MS/MS [122]. In diesen Laboren zeigt sich insgesamt eine deutlich niedrigere Recall-Rate.

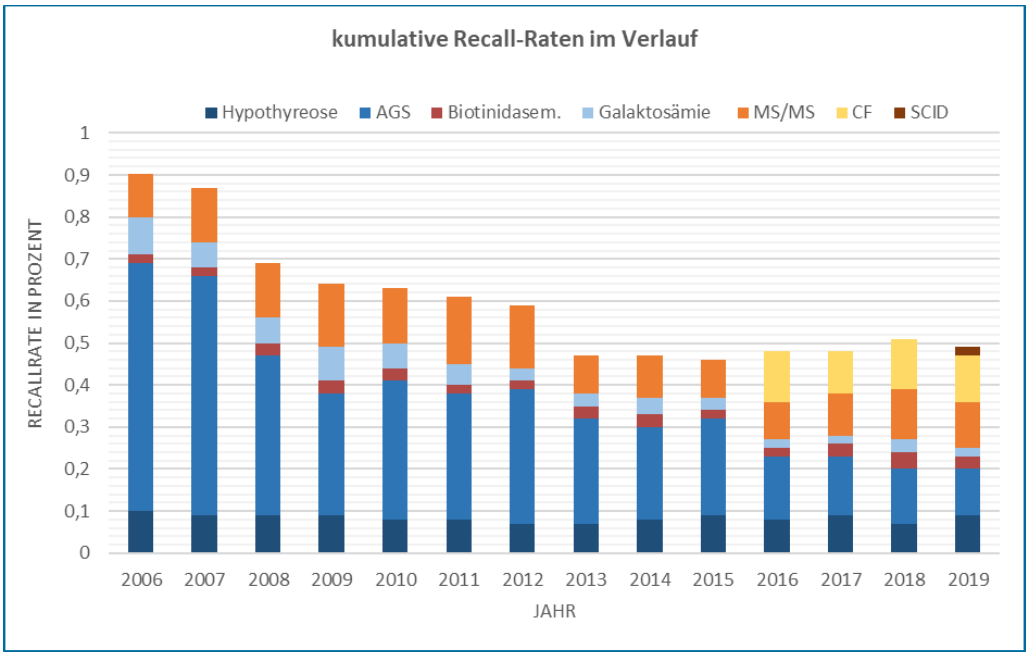

Abbildung 7 Verlauf der Recall-Raten über die Zeit und nach Krankheiten

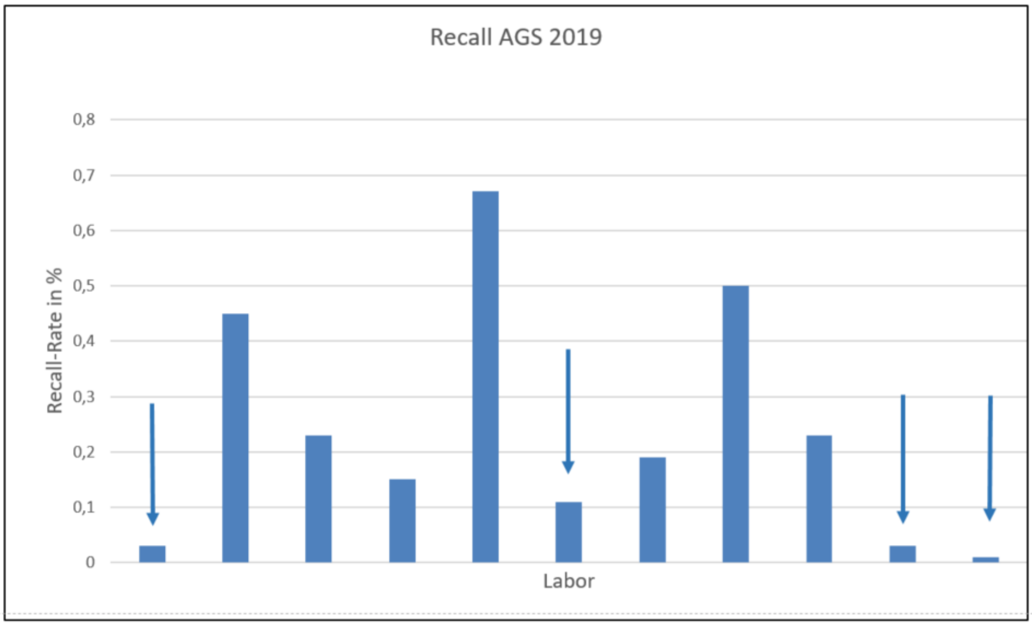

Abbildung 8 Recall-Raten AGS aus DGNS 2019; Einfluss Second-Tier-Verfahren

Betrachtet man die Recall-Rate der einzelnen Labore, so zeigen sich zwischen den Laboren deutliche Unterschiede (Spanne der Mittelwerte 2006-2019 nach Labor zwischen 0,03 % und 0,12 %). Dies kann, neben dem Einsatz von Second-Tier-Verfahren, durch unterschiedliche Methoden, bewertete Parameter und Cut-off-Werte (insbesondere bei Fettstoffwechselstörungen) oder aber die unterschiedliche Definition eines Recalls bedingt sein. So werden positive (auffällige) Befunde bei Blutentnahme unter 36 Stunden oder vor 32 SSW in einigen Laboren als Recall definiert, in anderen dagegen als wiederholungsbedürftiges Screening aufgrund des Abnahmezeitpunktes. In Abbildung 9 wird die Spannweite zwischen den Laboren durch Boxplots dargestellt.

Prozesszeiten

Als Zielgrößen für die Prozesszeiten sind in der Richtlinie die Blutentnahme von 36 bis 72 Lebensstunden, der Probenversand am Tag der Blutentnahme sowie die Mitteilung eines auffälligen Befundes spätestens 72 Stunden nach Probenahme vorgesehen. Detailliert beschrieben sind die

einzelnen Schritte des Screening-Prozesses in einer AWMF-Leitlinie [123]. Nach der Richtlinie sollen pathologische Befunde am Tag des Probeneingangs im Labor dem Einsender mitgeteilt werden. Alle Labore informieren bei hochpathologischem Befund mit dringendem Handlungsbedarf den Einsender sofort telefonisch, bei leicht auffälligen Befunden wird die Dringlichkeit der Mitteilung von den Laboren unterschiedlich gesehen. Eine Befundübermittlung im vorgesehenen Zeitraum ist nicht für alle Untersuchungen machbar, da z.B. der dreistufige Screening-Algorithmus beim CF-Screening länger als einen Tag dauert. Bisher wird für den DGNS-Report die Dauer der Befundmitteilung unabhängig vom Ergebnis erfasst und unterscheidet sich zwischen den Laboren deutlich. Die Unterschiede zwischen den Laboren und der geringere Anteil von Befundungen innerhalb von 24 Stunden seit Einführung des CF-Screenings (September 2016) sind in Abbildung 10 deutlich zu sehen.

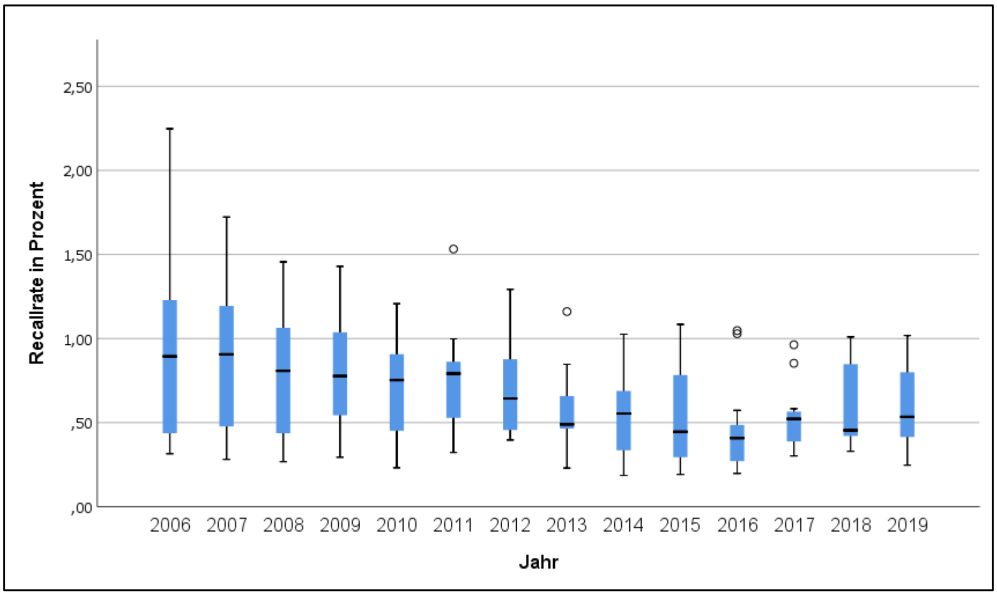

Abbildung 9 Spannweite der Recall-Raten zwischen den Laboren

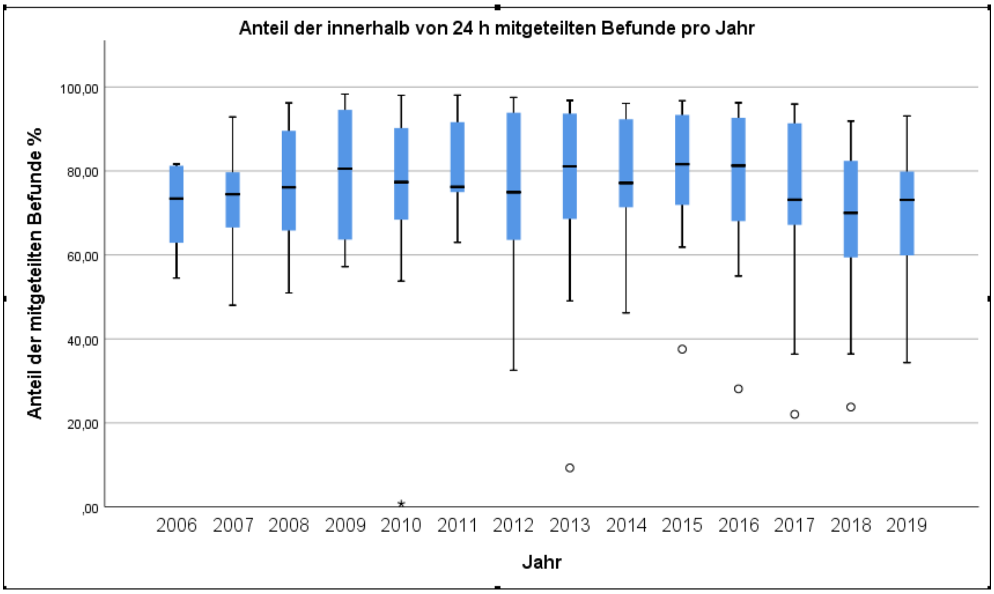

Abbildung 10 Anteil der innerhalb von 24 Stunden mitgeteilten Befunde, Spannweite der Labore

Die blauen Kästchen stellen den Median und Interquartilsbereich (IQR) dar. Die Linien außerhalb der Kästchen liegen zwischen den Minimal- und Maximalwerten, mit Ausnahme von Ausreißern >1,5 IQR von der Box, die durch Kreise und Sterne dargestellt sind.

3.3.2.3 Einzelinterviews und Gruppendiskussion mit Vertreter*innen der Labore

Die Analytik erfolgt in den Laboren weitgehend mit den in der Kinder-Richtlinie vorgeschriebenen Methoden. Dies bereite zwar keine Probleme, erlaube aber auch keine Optimierung der Analytik, da eine regelmäßige Aktualisierung der Methodenvorgaben für bereits etablierte Zielkrankheiten nicht erfolgt. Die interviewten Vertreter*innen der Labore forderten kontinuierliche Anpassungen und Aktualisierungen der Methodik inklusive der Aufnahme von Second-Tier-Strategien. Dabei bestand Konsens, dass der Einsatz von mehrstufigen (Second-Tier-) Methoden sinnvoll, aber aufwändig im Hinblick auf evtl. zusätzlich benötigte Geräte, höhere Kosten und den Zeitfaktor ist (geringe Probenzahl für Second-Tier-Analyse). Eine Second-Tier-Pflicht („da, wo Second-Tier-Methoden den Screening-Prozess signifikant verbessern können") mit einer Regelung zur Finanzierung und Ermöglichen einer Probenweitergabe an andere Labor für die Second-Tier Analyse wurde angeregt, dabei sind zeitkritische Befunde besonders zu berücksichtigen. Generell wurde der Wunsch nach mehr Mitsprache und Austausch mit den Laboren bei den Regelungen geäußert. Einige Vertreter*innen der Labore hielten eine Freigabe der Analysemethoden bei Vorgabe von Qualitätsstandards für effektiver. Sie möchten auf Basis von aktuellen Erkenntnissen aus Studien die Methoden selbständig und angepasst an die Voraussetzungen des eigenen Labors auswählen. Dabei zeigten sie eine hohe Bereitschaft zur Teilnahme an erweiterten internen und externen Qualitätskontrollen. Als zusätzliches Argument für eine Freigabe wurde die damit geringere Abhängigkeit von einzelnen Anbietern von Analysegeräten und Test-Kits und damit mehr Wettbewerb genannt.

Wichtig sei die Unterscheidung in Methodenqualitätskriterien einerseits und Effektivität des Screenings andererseits. Diese zeige sich im Anteil der bestätigten Diagnosen unter den auffälligen (richtig positiv) und unauffälligen (falsch negativ) Screeningbefunden. So sei laut Meinung einiger Teilnehmer*innen wichtig, dass die Interpretation der Ergebnisse bzw. die Definition von Cut-off-Werten laborübergreifend einheitlicher gestaltet werde und die Labore kontinuierlich voneinander lernen. Des Weiteren wurde angeregt, Qualitätsanpassungen auf Basis des jährlichen DGNS-Reports vorzunehmen.

Die Einführung weiterer Zielkrankheiten sahen die Vertreter*innen der Labore prinzipiell als unproblematisch für die zeitlichen Abläufe des Screenings an, wenn auch das CF-Screening durch den dreistufigen Algorithmus und einer evtl. späteren Befundmitteilung von einigen Laboren als Herausforderung betrachtet wird. Bei einer zunehmenden Anzahl von Zielkrankheiten wurde eine zeitlich weniger strikte Befundmitteilung, gestaffelt nach Dringlichkeit, angeregt. Auch die aktuelle Blutmenge auf den Testkarten sei ausreichend für weitere Untersuchungen, sofern die Filterkarten gut betropft wären.

Sowohl in den Einzelinterviews als auch in der Gruppendiskussion mit den Vertreter*innen der Labore wurden zu Qualitätssicherung in Analytik und Befundung folgende Gedanken geäußert: Einerseits wurde die Auffassung vertreten, erweiterte Qualitätsvorgaben seien nicht wichtig, weil die Labore aus eigenem Interesse auf hohe Qualitätsstandards bei den Analysen achten und diese einhalten würden. Die derzeit für die Qualitätssicherung vorgesehene Akkreditierung wurde kontrovers bewertet, dabei reichte die Spannweite von notwendig und sogar hilfreich bis überflüssig und zu teuer. Die Kritik lautete, dass die Akkreditierung nicht die wesentlichen Qualitätskriterien erfasse und daher keine Gewährleistung guter Screening-Qualität biete. Ein Ausbau von Ringversuchen (evtl. auch Ringversuch-Anbieter aus dem Ausland), die Aufnahme zusätzlicher Parameter in die RiLiBÄK sowie Qualitätszirkel der Labore mit dem Ziel kontinuierlicher Qualitätsverbesserung wurden vorge-

schlagen. Andererseits wurde mit Hinweis auf die in den DGNS-Reports [25] erkennbaren Qualitätsunterschiede zwischen den Laboren (Abbildung 8 bis 10) ein besseres Qualitätsmanagement angeregt. Qualitätsprobleme und Verbesserungspotentiale werden weniger im Bereich der Methoden- und Analysequalität als vielmehr im Bereich der Ergebnisqualität gesehen. Kaum Interesse wurde jedoch an CLIR geäußert. Dieses wurde wegen des hohen Teilnahme-Aufwands und der Datenhaltung in den USA eher kritisch bewertet, nationale Qualitätszirkel wurden favorisiert.

3.3.3 Best-Practice-Modelle

Weltweit genutzte Verfahren der Qualitätssicherung in Screeninglaboren mit besonders hohen Standards stammen aus den USA. Am „Newborn Screening Quality Assurance Program" (NSQAP) des Centers for Disease Control and Prevention (CDC) beteiligen sich alle US-Screeninglabore und Labore aus über 80 Ländern weltweit [124] (in Europa z.B. Norwegen, Österreich, Portugal, Slowenien, England). NSQAP ist ein Mehrkomponentenprogramm, das umfassende Qualitätssicherungsdienste für NBS-Trockenblut-Analysen bietet, insbesondere die regelmäßige Bereitstellung von Referenzmaterialien [124, 125]. Das digitale postanalytische Tool CLIR [120], dessen Verwendung die Befundung der gemessenen Ergebnisse deutlich verbessern kann [121, 126], wird in zahlreichen US-Bundesstaaten und weltweit genutzt (in Europa z.B. Norwegen [52] und Schweden [127]).

In England gibt es umfassende Regelungen zur Qualitätssicherung der Arbeit der 16 Screeninglabore (allerdings nur 9 Zielkrankheiten). „Laboratory Guides" enthalten detaillierte Verfahrensanweisungen zu Analysen und Befundungen, darunter auch Beschränkungen der zugelassenen Analysemethoden [105, 106]. Die Labore sind im gesamten UK in ein externes Qualitätskontrollsystem eingebunden (monatlich drei Ringproben für jeden Parameter, Meldung bei anhaltend schlechten Ergebnissen) [128]. In den Niederlanden wird eine einheitliche Durchführung und Qualitätssicherung der NBS-Analytik und -Befundung dadurch erreicht, dass das staatliche Screeninglabor, eines von insgesamt fünf Screeninglaboren, gleichzeitig als nationales NBS-Referenzlabor [129] fungiert. Das Referenzlabor legt in regelmäßiger Absprache mit den anderen Screeninglaboren Methoden, Verfahrensanweisungen und einheitliche Cut-off-Grenzen fest [130], die regelmäßig evaluiert und überarbeitet werden. Es erstellt außerdem vierteljährlich und jährlich Qualitätsberichte und leitet daraus ggf. Maßnahmen ab [129].

Biochemische Second-Tier-Verfahren, insbesondere für AGS und einzelne Stoffwechselerkrankungen (z.B. MSUD, IVA, Tyrosinämie) werden verbreitet eingesetzt, zunehmend auch molekulargenetische Second-Tier-Verfahren. In Europa erreichen z.B. Dänemark [54] und Norwegen [131] besonders niedrige Falsch-Positiven-Raten durch molekulargenetische Second-Tier-Analysen nach auffälligen biochemischen Screeningbefunden.

3.3.4 Zusammenfassung

- Screeninglabore sollten über entsprechende Fachkenntnisse verfügen, akkreditiert bzw. zertifiziert sein und an externen Qualitätssicherungs-/Kontrollprogrammen teilnehmen.
- Qualitätsmanagement:
    - Die Zielwerte und Benchmarks, die die Qualität und Wirksamkeit der Laborverfahren gewährleisten, sollten auf der Ebene der Programmverwaltung verbindlich festgelegt werden. In Ländern, in denen mehrere Laboratorien NBS-Blutspots analysieren, ist eine Abstimmung zwischen diesen Laboratorien auf Programmebene erforderlich.

- Second-Tier-Verfahren sollten, wenn immer nötig und möglich, zur Senkung der Recall-Rate verwendet und finanziert werden.
- Die Qualitätssicherung muss nicht nur die Analytik, sondern auch die Befundung umfassen. Der Einsatz von post-analytischen multivariaten digitalen Interpretations-werkzeugen auf Basis von Machine-Learning-Modellierungen bzw. Big-Data-Analysen (KI) kann zur Senkung der Rate falsch positiver und falsch negativer Befunde beitragen.
- Die Festlegung von Qualitätszielen, eine regelmäßige Evaluation, ggf. Anpassung und Optimierung von Methoden und Analysen wird allgemein empfohlen.

3.3.5 Konzeptvorschläge: Struktur und Qualitätssicherung im Screeninglabor

Eine regelmäßige Überprüfung der in der Richtlinie vorgesehenen Analysemethoden für die einzelnen Zielkrankheiten ist aus wissenschaftlicher Sicht unerlässlich. Diese Aufgabe könnte auf Basis der jährlichen DGNS-Datenerhebung und einer kontinuierlichen Überprüfung des aktuellen wissenschaftlichen Standes durch eine zentrale Koordinierungsstelle (s. Kapitel 3.9) in Kooperation mit den Screeninglaboren übernommen werden. Bei Bedarf könnten auch Pilotprojekte zu neuen Methoden oder Algorithmen durchgeführt werden. Der Auftrag hierfür könnte nach entsprechender Bewerbungen und Eignungsprüfung an eines der elf Screeninglabore mit entsprechender Finanzierung vergeben werden. In der Richtlinie würde dann, unter Hinweis auf die Ergebnisberichte der Koordinierungsstelle und ggf. unter Berücksichtigung von Pilotprojekten, auf die Vorgabe von Methoden verzichtet werden. Von einer völligen Freigabe der Methoden ist eher abzuraten, da hier auch Methoden mit (noch) nicht nachgewiesener Evidenz eingesetzt werden könnten, die Vergleichbarkeit unter den Laboren erschwert würde und intensive externe Qualitätskontrollen erforderlich wären, um eine hohe Effektivität des Screenings sicherzustellen. Bei der Festlegung der Analysemethoden sollten zur Senkung der Rate falsch positiver Befunde auch Second-Tier-Analysen vorgesehen und finanziert werden.

Für die Analytik müssen Qualitätsziele laborübergreifend festgelegt und regelmäßig evaluiert werden. Um das Erreichen der Qualitätsziele zu prüfen wird eine Ausweitung von Ringversuchen und die Aufnahme weiterer Parameter aus Trockenblut in die RiLiBÄK empfohlen. Konsequenzen bei Nicht-Erfüllung der Vorgaben bzw. Anreize für qualitätsverbessernde Maßnahmen sind zu etablieren. Alternativ zur allgemeinen Akkreditierung könnten Peer Review Verfahren diskutiert werden. Ein enger Austausch (auch von anonymen Proben) zwischen den Screeninglaboren („Qualitätszirkel der Screeninglabore“) ist erforderlich, um eine Vergleichbarkeit der Analyseergebnisse zu erreichen und auch auftretende Schwierigkeiten, z.B. durch geänderte Test-KITs oder Gerätemängel, schnell zu erkennen.

Neben der Analyse ist auch die Beurteilung und Einordnung der Analyseergebnisse für die Screening-qualität wichtig. Postanalytische multivariate digitale Interpretationswerkzeuge wie z.B. CLIR werden dringend empfohlen, um die Herausforderung der Befundung bei etlichen der sehr seltenen Ziel-krankheiten des ENS, bei Frühgeborenen und Neugeborenen auf Intensivstation sowie bei der Einordnung hinsichtlich der Therapiebedürftigkeit einiger Krankheitsbilder (Phänotyp) zu bewältigen. Durch die Teilnahme aller Screeninglabore an einem solchen oder ähnlichen Verfahren, möglicher-weise auch auf nationaler oder europäischer Ebene, könnte die Effektivität des ENS in Deutschland sicherlich weiter verbessert werden. Innerhalb der Europäischen Union wäre ein solches Verfahren rechtlich unproblematisch, da überall die Europäische Datenschutzgrundverordnung (DSGVO) gilt.

Eine regelmäßige Evaluation des Screening-Prozesses (Analyse und Befundung) im Labor ist für die Qualitätssicherung notwendig. Statt der bisher vorgesehenen Qualitätsberichte der Screeninglabore an die zuständige KV könnte die Erstellung des DGNS-Reports in die Richtlinie aufgenommen werden. Diese Berichte sind als Grundlage eines Benchmarkings geeignet und sollten ggf. eine Verpflichtung zur Verbesserung der Qualität auslösen.

3.4 Mitteilung des (auffälligen) Screeningbefundes

3.4.1 Ergebnisse der Literaturrecherche

Ein pathologischer (auffälliger) Screeningbefund bedeutet noch keine Diagnose, sondern (bei Symptomfreiheit) zunächst nur einen Krankheitsverdacht, der durch geeignete Methoden zeitnah bestätigt oder ausgeschlossen werden muss (z.B. zweite Testkarte oder Konfirmationsdiagnostik) und deutlich häufiger ist als in der Folge tatsächlich bestätigte Diagnosen [9]. Die Mitteilung eines pathologischen Befundes an die Eltern stellt insofern eine Herausforderung dar, als einerseits die Dringlichkeit der weiteren Untersuchungen vermittelt und andererseits dabei unnötige Verunsicherung möglichst vermieden werden soll [9, 30, 132]. Dabei spielen sowohl die Inhalte als auch die Art der Kommunikation und die emotionale Unterstützung der Eltern eine wichtige Rolle [133, 134]. Die Bedeutung der kompetenten Mitteilung durch eine sowohl über die Prozesse als auch über die Krankheit gut informierte Person wird in der Literatur betont [30, 132, 135]. Dies ist unabhängig davon, ob sich der Krankheitsverdacht bestätigt oder nicht [133]. Als optimal gilt die Befundmitteilung an die Eltern durch Expert*innen, die dann auch die Abklärung durchführen [136]. Studien ergaben, dass Eltern die Mitteilung durch eine bekannte Person (z.B. Kinder- oder Familienarzt/-ärztin) bevorzugen. Dies ist jedoch mit dem Risiko zeitlicher Verzögerungen verbunden [137] und gilt nur dann als sinnvoll, wenn die informierenden Personen entsprechend instruiert und geschult sind [30, 132, 134, 138]. Um die belastende Phase der Ungewissheit für die Eltern möglichst kurz zu halten, sollte die Zeitspanne zwischen Befundmitteilung und Termin zur Abklärung möglichst kurz gehalten werden [30, 139, 140].

Das Europäische Expertengremium fordert im European Expert Document [30], dass Informationsinhalte und Kommunikationsrichtlinien für die Mitteilung pathologischer Befunde an die Eltern zentral auf Ebene der Programmleitung festgelegt und veröffentlicht werden sollen. Auch wird dort die Mitteilung aller Ergebnisse, einschließlich unauffälliger Ergebnisse, empfohlen, da dies zur Qualitätskontrolle und zum Wohlbefinden der Eltern beitragen kann. Chudleigh et al. [141] üben deutliche Kritik an dem für Deutschland vorgegebenen Ablauf. Das Prinzip der Autonomie (freie Arztwahl) wirke hier den Prinzipien des Nutzens und Nicht-Schadens entgegen.

3.4.2 Situation in Deutschland

3.4.2.1 Rechtliche Situation

Die Mitteilung des Screeningbefundes ist in §22 Kinder-Richtlinie [23] entsprechend der gesetzlichen Vorgaben des GenDG geregelt. Unauffällige Befunde werden vom Labor schriftlich an den Einsender übermittelt und den Eltern nicht regelhaft mitgeteilt. Auffällige Befunde sollen vom Labor an den Einsender, als verantwortliche ärztliche Person im Sinne des GenDG, übermittelt werden und dürfen den Eltern nur von diesem mitgeteilt werden. Eine Ausnahmeregelung gilt laut Richtlinie nur, wenn der Einsender nicht erreichbar und eine weitere Befundabklärung dringlich ist. In diesen Fällen darf

der Laborarzt oder die Laborärztin die Eltern direkt informieren. Der Einsender (ggf. Laborarzt oder -ärztin) weist die Eltern auf die „Notwendigkeit einer schnellen, fachkompetenten Abklärung und Weiterbetreuung“ hin und nennt ihnen Kontaktmöglichkeiten zu den nächsterreichbaren spezialisierten Zentren (Behandlungszentren). Die Eltern sollen dann ein Zentrum auswählen und einen Termin vereinbaren.

Das GenDG [24] geht von einer verantwortlichen ärztlichen Person aus, die Aufklärung, Einwilligung (ggf. Blutentnahme) und Befundmitteilung für eine genetische Untersuchung übernimmt. Dieses Konstrukt passt für das ENS insofern nicht, als dass in der Regel die (ärztliche) Person, die die Geburt verantwortlich geleitet hat, für die Durchführung des Screenings verantwortlich, aber weder Vertrauensperson der werdenden Mutter noch besonders kompetent im Hinblick auf die 17 seltenen Zielkrankheiten ist [142]. Diese ärztliche Person ist den meisten Frauen, die zur Entbindung nur für kurze Zeit stationär aufgenommen werden, bis dahin nicht vertraut. Auch sind die Frauen in der Regel bei Eingang des Screeningbefundes schon entlassen und werden, aufgrund der Dienstzeiten in der Klinik, in der Regel durch eine andere ärztliche Person aus der Geburtsklinik über den pathologischen Screeningbefund telefonisch informiert. Eine Befundmitteilung durch den Einsender entspricht weder den oben beschriebenen Forderungen in der aktuellen Literatur noch der Intention des GenDG, das §7 GenDG in der Gesetzesbegründung dahingehend kommentiert, dass der Arztvorbehalt für eine „angemessene und kompetente Befundmitteilung“ sorgt [24].

3.4.2.2 Online-Befragung der Geburtskliniken

Bei der Mitteilung auffälliger Befunde muss zwischen grenzwertigen bzw. unklaren und hochpathologischen Befunden differenziert werden. Über erstere werden die Eltern in der Regel telefonisch durch eine ärztliche Person (69,1 %), teilweise auch durch Pflegepersonal oder Hebamme (37,1 %) und bei 1,6 % durch ein Sekretariat informiert. Hochpathologische Befunde, die einer raschen Abklärung bedürfen, werden den Eltern in 56,2 % der Fälle ausschließlich ärztlich, zu über einem Fünftel jedoch auch (21,7 %) oder nur (9,8 %) durch Pflegefachkräfte und in einzelnen Fällen durch ein koordinierendes Screeningzentrum oder ein spezialisiertes Behandlungszentrum mitgeteilt. Eine Mehrheit der Befragten hielt eine Befundmitteilung durch Expert*innen aus den Zentren für die Konfirmationsdiagnostik grundsätzlich (56,2 %) oder zumindest bei bestimmten Zielkrankheiten (31,4 %) für sinnvoll (weitere Ergebnisse zur Befundmitteilung s. Anhang 2).

3.4.2.3 Einzelinterviews und Gruppendiskussionen

Auch die meisten Interviewpartner*innen aus den Laboren, den pädiatrischen Praxen sowie die Hebammen hielten die Beteiligung von ärztlichen Expert*innen bei der Mitteilung eines auffälligen Befundes an die Eltern für sinnvoll, ein kleinerer Teil betont die Vorteile einer Befundmitteilung durch eine bekannte und vertraute Person. Ähnlich war das Ergebnis der Gruppendiskussion mit Vertreter*innen der Screeninglabore, in der betont wurde, dass den Einsendern häufig die notwendige Kompetenz bei der Befundmitteilung fehle und es sich bei ihnen auch nicht um Personen handele, zu denen von Seiten der Eltern ein Vertrauensverhältnis bestehe. Die auf dem GenDG basierenden Regelungen der Kinder-Richtlinie verhinderten Befundmitteilung nach dem Leitgedanken „Was ist das Beste für das Kind?“. Als entscheidender Faktor für eine gelungene Kommunikation mit den Eltern wurde das Netzwerk Labor – Einsender – Expert*innen betrachtet. Die bestehenden Regelungen verhindern jedoch den hier notwendigen Informationsaustausch zwischen den Akteuren. Ideal wäre die direkte Weitergabe auffälliger Befunde von den Screeninglaboren an spezialisierte Zentren, so dass eine Information der Eltern direkt durch Expert*innen

möglich wäre. Auch in der Fokusgruppendiskussion mit den Teilnehmer*innen aus spezialisierten Behandlungszentren bestand Konsens, dass die Mitteilung auffälliger Screeningbefunde an die Eltern durch Expert*innen und nicht durch den Einsender erfolgen sollte. Die größte Hilfe wäre es, wenn Behandlungszentren offiziell die Kontaktaufnahme zu den Eltern übernehmen dürften. Als Vorteil dieses Vorgehens wurde die Kompetenz der Expert*innen bei Fragen der Eltern angesehen. Auch könne direkt der Terminplan des Zentrums bereitgehalten werden, so dass die Eltern je nach Dringlichkeit zeitnah einbestellt und eine schnelle Abklärung gewährleistet werden könnte. Diskussionsteilnehmer*innen berichteten, dass die überwiegende Mehrheit der Eltern dankbar sei, wenn ihnen die anstehenden Schritte erläutert würden, bei denen sie begleitet und nicht allein gelassen würden. Ein weiterer Benefit wurde darin gesehen, dass die Konfirmationsdiagnostik in einem Zentrum mit nachgewiesener Kompetenz durchgeführt wird.

In den Gruppen wurde vorgeschlagen, dass die Zuweisung an das Zentrum vom Labor über die Postleitzahl der Eltern erfolgen könnte. Eine Vergütung der Zentren für die Kontaktaufnahme wurde angeregt. Dringender Regelungsbedarf wurde hinsichtlich eines einheitlichen Organisationsablaufs gesehen.

3.4.3 Best-Practice-Modelle

Die Information der Eltern über hoch auffällige Befunde erfolgt z.B. in Dänemark [143], Portugal [144], Schweden [145], England [146] und Österreich [persönliche Mitteilung] durch Expert*innen bzw. ein Behandlungszentrum. In Ontario werden Eltern über alle auffälligen Befunde, auch leicht auffällige, durch gut geschultes Fachpersonal eines Behandlungszentrums informiert; ein Arzt oder eine Ärztin wird hierbei nicht zwingend eingebunden [persönliche Mitteilung]. In der Schweiz erfolgt die Mitteilung eines positiven CF-Screeningbefundes nur durch eines der acht CF-Zentren und erst kurz bevor ein Termin zur Abklärung angeboten werden kann [147]. Die direkte Weitergabe von Daten und Befunden an das zuständige spezialisierte Zentrum ist in diesen Ländern fester Bestandteil des Prozesses.

In Norwegen werden Eltern zunächst durch geschultes, erfahrenes Fachpersonal des Screeninglabors über auffällige Befunde informiert. Danach wird baldmöglichst eine weitere Besprechung in einem spezialisierten Behandlungszentrum organisiert, ggf. ersatzweise eine Besprechung in einer örtlichen Kinderklinik mit gleichzeitiger Video-Zuschaltung des Zentrums. Mit Videokonferenzen zwischen Eltern, Vertreter*innen eines Zentrums und Vertreter*innen der örtlichen Klinik möglichst bald nach Befundmitteilung wurden gute Erfahrungen gemacht [136, persönl. Mitt.].

Eltern-Informationen für die Situation „auffälliges Screening“ sind z.B. verfügbar in Neuseeland [148], England [149] und Ontario [150]. Diese Materialien beginnen mit Formulierungen wie „A ‘screen positive’ result does NOT mean that a baby has the disease. It means that the baby has a higher chance to have the disease and that more testing is needed to find out for sure.” In Ontario steht zusätzlich gut sichtbar auf der Eltern-Website des NBS-Programms ein direkter Link zu „Has your baby screened positive? Learn more about what this could mean“ [151]. Für unauffällige Befunde gibt es in einigen Ländern digitale Mitteilungs- oder Abfragemöglichkeiten für Eltern (z.B. Finnland [152], Israel [153], Portugal [154]). In den Niederlanden und in England werden unauffällige Screeningergebnisse den Eltern schriftlich mitgeteilt [155, 156].

3.4.4 Zusammenfassung

- Ablauf, Inhalt und Art der Mitteilung auffälliger Befunde an die Eltern sollen so gestaltet werden, dass die Eltern möglichst wenig verunsichert und beunruhigt werden. Wichtig ist dafür auch ein möglichst kurzes Zeitintervall zwischen Mitteilung und Abklärung des auffälligen Befundes.
- Die Mitteilung pathologischer Screeningbefunde an die Eltern bzw. behandelnden Ärzte oder Ärztinnen muss zeitgerecht erfolgen, da die Therapie bei den meisten Krankheiten rasch eingeleitet werden muss.
- Hochauffällige Befunde sollten den Eltern durch Expert*innen aus den Behandlungszentren, die auch die Konfirmationsdiagnostik durchführen können, mitgeteilt werden. Dies bietet folgende Vorteile:
  - Fragen der Eltern zu Krankheit, deren Auftretenswahrscheinlichkeit, nächste Schritte, Diagnostik und ggf. Therapie können direkt und kompetent beantwortet werden.
  - Der Terminplan des Zentrums ist bekannt, so dass Eltern nach der Befundmitteilung je nach Dringlichkeit zeitnah einbestellt werden können.
  - Die überwiegende Mehrheit der Eltern ist dankbar, wenn ihnen die nächsten Schritte vorgegeben werden, bei denen sie begleitet und nicht allein gelassen werden.
  - Kompetente und leitliniengerechte Konfirmationsdiagnostik werden durch direkte Einbindung von Expert*innen gewährleistet.
- Wünschenswert wäre die Mitteilung auch unauffälliger Befunde an die Eltern (digital).

3.4.5 Konzeptvorschläge: Mitteilung des (auffälligen) Screeningbefundes

Im ENS wird derzeit auf 17 seltene Zielkrankheiten aus sechs unterschiedlichen pädiatrischen Subspezialitäten gescreent. Nach der Richtlinie soll den Eltern bisher ein auffälliger Befund durch die für die Durchführung des Screenings verantwortliche (ärztliche) Person (in der Regel aus der Geburtshilfe) mitgeteilt werden. Allerdings können Kenntnisse zu den einzelnen (sehr) seltenen Erkrankungen und den notwendigen diagnostischen Abläufen bei dieser Person nicht vorausgesetzt werden. Deshalb sollen hochpathologische Befunde in der Regel über Expert*innen aus den Zentren für die Konfirmationsdiagnostik und Behandlung mitgeteilt werden, um eine kompetente Information der Eltern über den abzuklärenden Krankheitsverdacht sicherzustellen. Die Behandlungszentren könnten sich im Vorfeld über die Zuweisungsmodalitäten der Labore einigen (z.B. nach Postleitzahl, über Bereitschaftsdienstzeiten etc.). Pathologische und wiederholungsbedürftige Ergebnisse, bei denen keine Abklärung in einem Zentrum erforderlich ist, könnten den Eltern direkt aus dem Labor von geschultem Personal (evtl. auch nicht-ärztlich) mitgeteilt werden. Sowohl für die Befundmitteilung durch Expert*innen aus den Behandlungszentren als auch von Ärzten oder Ärztinnen aus den Screeninglaboren sollte eine Anpassung des GenDG geprüft werden. Die Gendiagnostikkommission hält eine Befundmitteilung in Notsituationen, in denen Gefahr für das Leben und die körperliche Unversehrtheit der betroffenen Person besteht, durch andere in gleicher Weise kompetente Personen für zulässig. In ihrer Richtlinie zur Aufklärung [157] sieht sie eine solche Notsituation bei auffälligen Befunden im derzeitigen ENS und Mukoviszidose-Screening als gegeben an. Vor Auftragserteilung könnte hierzu eine schriftliche Einwilligung der Personensorgeberechtigten eingeholt werden. Der Befund muss möglichst zeitnah zum Termin für die Konfirmationsdiagnostik mitgeteilt werden. Ein solches Konzept kann medizinische Nachteile für die Kinder vermeiden und das Kindeswohl besser schützen als ein Gesetzesverständnis, welches sich streng am Wortlaut des §11 Abs.1 GenDG orientiert.

Standardisierte Schulungs- und Informationsmaterialien für Ärzte und Ärztinnen und gut zugängliche Informationen für Eltern bei der Situation „auffälliges Screeningergebnis“ sollten auf einer zentralen Screening-Website zur Verfügung gestellt werden. Zusätzlich könnten den Eltern bei einem auffälligen Screeningbefund auch schriftliche Informationen per Mail (verschlüsselt) oder Post zugesandt werden. Um das Risiko zu minimieren, dass Screeninguntersuchungen unbeabsichtigt unterbleiben, z.B. weil Testkarten auf dem Weg ins Labor verloren gehen, könnten unauffällige Befunde sowohl für Eltern als auch für pädiatrische Praxen in einem digitalen Portal mit einer eineindeutigen Screening-ID (s. unten) abrufbar sein.

3.5 Konfirmationsdiagnostik

3.5.1 Ergebnisse der Literaturrecherche

Wesentliche Elemente für den Erfolg eines Screening-Programms sind sowohl eine zeit- und leitliniengerechte Diagnostik zur Abklärung eines auffälligen Screeningbefundes (Konfirmationsdiagnostik) und ggf. ein frühzeitiger Therapiebeginn als auch eine von Anfang an fachlich und kommunikativ kompetente Begleitung der Eltern. Da alle Zielkrankheiten des ENS zu den (sehr) seltenen Erkrankungen gehören, bei denen die Patient*innen auf eine hochkompetente, spezialisierte Diagnostik und medizinische Versorgung angewiesen sind, wird diese am besten in spezialisierten Fachzentren (Behandlungszentren) gewährleistet [158–160]. Solch eine Zentrenbildung ist eine zentrale Forderung der europäischen Ratsempfehlung für den Bereich seltener Erkrankungen [160, 161]. Die Auswahl der Zentren sollte über die Fachgesellschaften nach objektiv festgelegten Qualitätskriterien erfolgen und auch kontrolliert werden. Empfehlungen zu Qualitätskriterien für Fachzentren für seltene Erkrankungen wurden vom EU-Sachverständigenausschuss herausgegeben [161].

In der Literatur wird die Festlegung von eindeutigen Falldefinitionen [30, 63, 66, 108, 162–164] sowie von "Diagnoseprotokollen" gefordert, die sich direkt auf die Falldefinition beziehen und beschreiben, wer als Patient*in zu behandeln ist. Sie sollten auf Programmebene verfügbar sein und auf Leitlinien für die Konfirmationsdiagnostik beruhen [8, 30]. Insbesondere bei neuen Zielkrankheiten mit unterschiedlichen Phänotypen wird dies als extrem wichtig angesehen [165].

3.5.2 Situation in Deutschland

In Deutschland fehlen Falldefinitionen und einheitliche zentrale Vorgaben zur Konfirmationsdiagnostik. Allerdings sind für nahezu alle Zielkrankheiten AWMF-Leitlinien der entsprechenden Fachgesellschaften verfügbar [159, 166–170]. Die Screeninglabore müssen laut Kinder-Richtlinie (§5 Abs.3) aktuelle Verzeichnisse der „nächsterreichbaren Zentren mit pädiatrischen Stoffwechselexpert*innen oder Endokrinolog*innen oder Hämatolog*innen sowie spezialisierten immunologischen oder neuromuskulären Einrichtungen mit 24-stündiger Erreichbarkeit“ zur Verfügung stellen. Qualitätsanforderungen für diese Zentren sind nicht definiert. Für die neu ins Screeningpanel aufgenommenen Zielkrankheiten (SCID, SMA und Sichelzellkrankheit) haben die jeweiligen Fachgesellschaften qualifizierte Zentren nach einem transparenten Verfahren mit definierten Qualitätskriterien benannt. Regelungen für eine Zulassung von Zentren oder Kliniken zur qualifizierten Konfirmationsdiagnostik existieren jedoch nicht. Das bedeutet, dass jede Kinderklinik theoretisch die Abklärung bei Verdacht auf eine der sehr seltenen Krankheiten vornehmen kann.

3.5.2.1 Gruppendiskussionen mit Vertreter*innen der Labore und Zentren

In den Diskussionen mit Vertreter*innen der Labore und Zentren bestand, wie auch in der Literatur, Einigkeit, dass die Konfirmationsdiagnostik ausschließlich in kompetenten Zentren erfolgen sollte. Diese könnten über die Fachgesellschaften nach objektiv festgelegten Qualitätskriterien ausgewählt und auch kontrolliert werden. Ideal wäre eine konkrete Zuweisung der Kinder mit auffälligem Befund vom Screeninglabor an das nächstgelegene Zentrum, das dann die Kontaktaufnahme zu den Eltern und die Koordination der zeitnahen Konfirmationsdiagnostik und ggf. des Therapiebeginns übernimmt. Denkbar wäre auch, dass dieses Zentrum mit den Eltern bespricht, ob diese ggf. in ein anderes anerkanntes Zentrum zur Abklärung des Screeningbefundes gehen wollen. Eine Rückmeldung der Ergebnisse der Konfirmationsdiagnostik an das Screeninglabor sollte zu Zwecken der Qualitätssicherung von den Zentren zugesichert werden.

3.5.3 Best-Practice-Modelle

In vielen Screening-Programmen ist vorgegeben, dass die Konfirmationsdiagnostik nach auffälligem Screeningbefund (häufig mit Ausnahme der Hypothyreose) nur in spezialisierten Behandlungszentren durchgeführt wird, z.B. in Dänemark [143], Norwegen [136] , Niederlande [171], Österreich [persönliche Mitteilung], Ontario [79], Portugal [144], Schweden [145] oder England [146]. Die Zuteilung eines Behandlungszentrums und die direkte Übermittlung der Screeningbefunde an dieses Zentrum sind dabei in der Regel fester Bestandteil des Prozesses.

Beispiele für geeignete Diagnoseprotokolle und Falldefinitionen sind die regelmäßig aktualisierten Flowcharts zur Konfirmationsdiagnostik in der kanadischen Provinz Ontario [persönliche Mitteilung], die Vorgaben und Schemata zur Konfirmationsdiagnostik in den „laboratory guides" des NBS-Programms in England [105], sowie aus den USA die Diagnose-Algorithmen des American College of Medical Genetics and Genomics (ACMG) [172] und die Falldefinitionen der Association of Public Health Laboratories (APHL) [173]. Diese wurden im Rahmen des Programms „New Steps" in iterativen Prozessen von Experten staatlicher NBS-Programme entwickelt, um einheitliche Klassifizierungen für Diagnosen in verschiedenen Programmen zu erleichtern.

Die Rückmeldung der Ergebnisse der Konfirmationsdiagnostik an das Screeninglabor bzw. -zentrum ist in mehreren Ländern vorgesehen; in Kalifornien und den Niederlanden erfolgt sie über direkte Eingaben der Einrichtung, die die Diagnostik durchführte, in eine vom Screening-Programm bereitgestellte Datenbank [174–176].

3.5.4 Zusammenfassung

- Die zeit- und leitliniengerechte Diagnostik eines auffälligen Screeningbefundes sowie die kompetente Begleitung der Eltern sind für den Screeningerfolg von hoher Bedeutung.
- Falldefinitionen und eine leitliniengerechte Konfirmationsdiagnostik sind notwendig.
- Die Konfirmationsdiagnostik sollte nur in Fachzentren (Behandlungszentren) erfolgen. Die Auswahl der Zentren kann über die Fachgesellschaften nach objektiv festgelegten und transparenten Qualitätskriterien erfolgen und auch kontrolliert werden. Empfehlungen zu Qualitätskriterien für Fachzentren für seltene Erkrankungen wurden vom EU-Sachverständigenausschuss herausgegeben.
- Eine Rückmeldung der Konfirmationsdiagnostik an die Screeninglabore dient der Qualitätssicherung der Analytik und unterstützt ein effektives Tracking (s. Kapitel 3.6.)

3.5.5 Konzeptvorschläge: Konfirmationsdiagnostik

Eine deutschlandweite Vereinheitlichung des Prozessablaufs nach einem auffälligen Screeningbefund mit der Zuweisung zum Behandlungszentrum und der Rückmeldung der Befunde aus der Abklärung der Zielkrankheiten (Konfirmationsdiagnostik) wird dringend empfohlen.

Die Konfirmationsdiagnostik muss in einem qualifizierten Fachzentrum leitliniengerecht erfolgen. Hierfür sind die Festlegung von Falldefinitionen und Qualitätskriterien notwendig. Qualifizierte Zentren für die Konfirmationsdiagnostik könnten nach definierten und transparenten Kriterien von den Fachgesellschaften benannt und die Einhaltung der Kriterien kontrolliert werden. Da den meisten Zielkrankheiten genetische Ursachen zu Grunde liegen, muss im Rahmen der Konfirmationsdiagnostik eine qualifizierte genetische Beratung angeboten werden. Damit darf die Konfirmationsdiagnostik nur in Zentren erfolgen, die eine entsprechende Kompetenz vorhalten.

Für die Evaluation des Screening-Prozesses müssen die Ergebnisse der Konfirmationsdiagnostik den Screeninglaboren oder einer zentralen Stelle mitgeteilt werden. Auch sollten alle diagnostizierten Fälle in ein Register aufgenommen werden (s. auch Kapitel 3.7).

3.6 Tracking

Die Sicherstellung des Screening-Angebots an alle Neugeborenen (unabhängig von ihrem sozialen, ethnischen oder religiösen Hintergrund) und die Sicherstellung einer vollständigen Abklärung aller wiederholungsbedürftigen oder auffälligen Screening-Untersuchungen bis zum Ausschluss oder der Bestätigung einer Zielkrankheit sind entscheidend für den Erfolg und zugleich herausfordernd für ein NBS-Programm. Dies liegt u.a. daran, dass die Zielkrankheiten selten sind und die Einsender von Screeningproben (Geburtskliniken, pädiatrische Praxen, Hebammen) in ihrem gesamten Berufsleben in aller Regel mit sehr wenigen betroffenen Kindern konfrontiert werden und deshalb mit diesen Krankheiten nicht vertraut sind. Die Häufigkeiten variieren je nach Zielkrankheit zwischen 1:3.000 bis weniger als 1:100.000 Neugeborenen; insgesamt ist etwa eines von 750 Neugeborenen von einer der Zielkrankheiten des Neugeborenen-Screenings (Stand 2022) betroffen (0,1% der Neugeborenen-Population). Bei 99,9% der Kinder führt das NBS also letztendlich zu einem unauffälligen (negativen) Befund. Die Einsender von Blutproben erleben in ihrem Berufsalltag, dass die allermeisten NBS-Screening-Untersuchungen ohnehin ein negatives Ergebnis liefern und selbst wenn das Ergebnis zunächst auffällig ist, die angeforderte Kontrolluntersuchung häufig zu einem unauffälligen Befund führt. Diese Erfahrungen erhöhen das Risiko die wenigen Betroffenen trotz NBS zu spät zu entdecken oder ganz zu übersehen und sind wichtige Argumente für eine übergeordnete zentrale Erfassung und Koordination (s. Kapitel 3.7 bis 3.9). Übersehene Fälle durch vermeidbare Fehler oder Nachlässigkeiten enden nicht selten in einer Tragödie für die Betroffenen und deren Familie. Hinzu kommt, dass beispielsweise jede vermeidbare lebenslange Behinderung mit hohen Kosten für Gesundheits- und Sozialkassen verbunden sein kann und auch aus gesundheitsökonomischer Sicht zu vermeiden ist.

3.6.1 Ergebnisse der Literaturrecherche

Eine systematische Erfassung der Zielpopulation ist die Grundbedingung für eine hohe Effektivität des Neugeborenen-Screenings. Im Rahmen von Trackingprogrammen wird überprüft, ob jedes Neugeborene vom NBS-Programm erfasst (Vollständigkeit) [9, 30, 66, 177], ob jeder notwendige Bestätigungs- oder Diagnosetest durchgeführt und bei Bedarf eine Behandlung eingeleitet wurde [9,

66, 178]. Eltern nicht gescreenter Kinder werden nochmals über das Screening informiert, an ausstehende Kontroll- oder Wiederholungsuntersuchungen werden sie erinnert (Tracking). Einheitliche zentrale digitale Informationssysteme mit Datenaustausch zwischen medizinischen Einrichtungen unterstützen und beschleunigen das Tracking [30]. Für den Erfolg eines Screening-Programms haben Trackingsysteme mit Qualitätssicherungsmerkmalen hohe Bedeutung [22], insbesondere für Kinder aus Familien mit geringem Bildungsstand oder schlechterem Zugang zur medizinischen Versorgung [179, 180].

3.6.2 Situation in Deutschland

Die Kinder-Richtlinie enthält keine Vorgaben zur Sicherstellung der Vollständigkeit, außer dass bei der Vorsorge-Untersuchung U2 (3.-10. Lebenstag) überprüft werden soll, ob das Screening im Kinder-untersuchungsheft dokumentiert ist; wenn nicht, soll es angeboten werden (§19 Abs.2) [23]. Damit ist jedoch nicht sichergestellt, dass die Blutprobe im Labor eingegangen ist. Weder sind Befund-rücklaufkontrollen beim Einsender verbindlich vorgeschrieben noch ist ein flächendeckender personenbezogener Abgleich von geborenen mit im Screening untersuchten Kindern vorgesehen. Dieser wird nur in Bayern und Berlin durchgeführt; in einigen Laboren wird an Hand der Geburten-buchnummern oder fortlaufender Labornummern die Vollständigkeit überprüft. Beide Systeme sind nicht optimal. Auch zur Sicherstellung, dass bei Neugeborenen mit dringend abklärungsbedürftigen Befunden im ENS die Konfirmationsdiagnostik und ggf. Behandlung durchgeführt wird (Tracking), fehlen Vorgaben in Deutschland.

3.6.2.1 Auswertung der DGNS-Daten 2006 bis 2019

Die DGNS-Datenanalyse lässt auf eine hohe Vollständigkeit des ENS schließen. So lag 2019 beispielsweise die von den Laboren kumulativ gemeldete Anzahl der Kinder mit Screening-Untersuchungen bei 99,98 % der Neugeborenen. Dieser Wert ist möglicherweise überschätzt, weil nicht als solche identifizierte Zweit- und Folgescreening-Untersuchungen zu Doppelerfassungen führen könnten.

Nach der Kinder-Richtlinie muss das Screening bei einer Blutabnahme vor 36 Lebensstunden oder vor 32 Schwangerschaftswochen ebenso wie bei auffälligen Befunden mit einer weiteren Testkarte wiederholt werden. Die Rate der eingegangenen Folgekarten lag für Deutschland insgesamt nur bei ca. 80 % der angeforderten Untersuchungen (Abbildung 3) [26] und differiert zwischen den einzelnen Laboren stark (74 % bis 100 %). Dies ist abhängig davon, ob und in welchem Umfang das Labor eine Nachverfolgung der ausstehenden Testkarte vornimmt (Tracking). Die Spannweite wird mit Boxplots in Abbildung 11 gezeigt (s. auch Anhang 1).

Zwischen 2006 und 2019 ist für ca. 10% der Kinder mit entsprechender Indikation unklar, ob eine endgültige Abklärung des auffälligen Screeningbefundes erfolgt ist (Abbildung 3) [12]. Während bei einigen Laboren die Ergebnisse der Konfirmationsdiagnostik fast vollständig vorliegen, liegt bei anderen die Loss-to-follow-up-Rate bei über 30 % (s. Anhang 1).

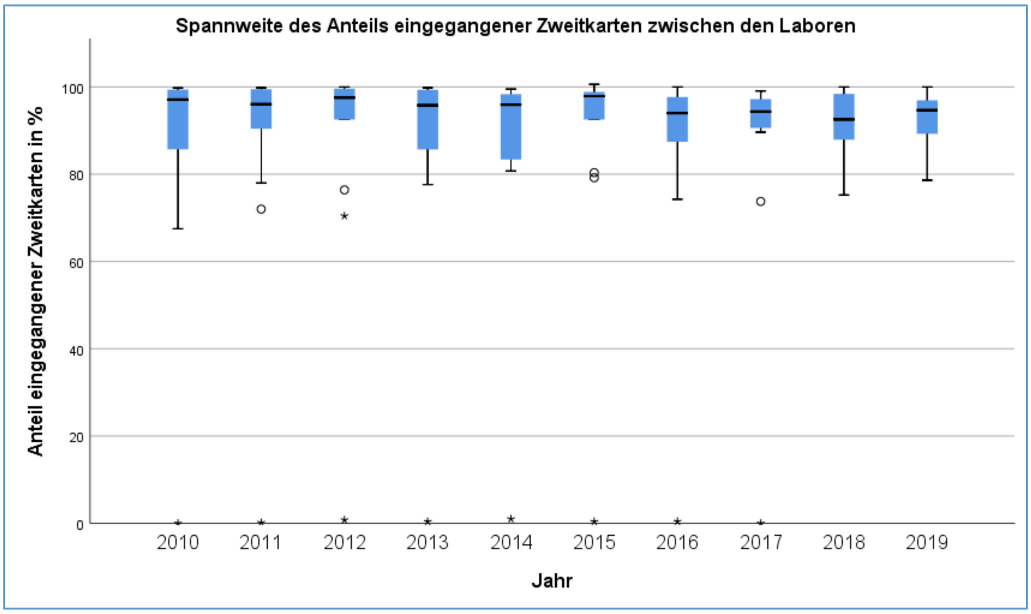

Abbildung 11 Anteil eingegangener Wiederholungs- und Kontrolluntersuchungen

3.6.2.2 Einzelinterviews und Gruppendiskussion mit Vertreter*innen der Labore

In den Interviews und der Diskussion mit Vertreter*innen der Labore wurde von allen Teilnehmer*innen die Etablierung eines funktionierenden Trackingsystems mit definierten Strukturen gefordert. Nur so könnten die aufwändigen Labormethoden in ein Gesamtsystem gestellt werden. Allerdings erfordere das Tracking sowohl finanzielle als auch personelle Ressourcen, die Vergütung müsse geklärt werden. Dabei wurde klargestellt, dass ein Abgleich mit den Geburtenmeldungen durch die Labore nicht geleistet werden könne und für bundeslandübergreifend arbeitende Labore auch nicht zielführend sei. Neue Methoden zur Überprüfung der Vollständigkeit sollten erarbeitet werden. Die Mehrheit der Teilnehmenden unterstützten die Idee, dass über eine zentrale digitale Plattform mit einheitlicher eineindeutiger Screening-ID als Pseudonym eine systematische Sicherstellung der Vollständigkeit ermöglicht werden kann. Zwei Teilnehmende könnten sich eine Verknüpfung mit dem Neugeborenen-Hörscreening vorstellen – auch um personelle und finanzielle Ressourcen im Tracking zu sparen.

Dringender Handlungsbedarf bestehe hinsichtlich einer datenschutzkonformen Regelung zum Austausch von medizinischen Informationen mit weiteren in die Diagnostik eingebundenen medizinischen Einrichtungen. Momentan könne ein laborübergreifendes Tracking oder die Weitergabe wichtiger Informationen für die Befundung einer Folgekarte oder bei der Bestätigungsdiagnose aus Datenschutzgründen in der Regel nicht erfolgen.

3.6.3 Best-Practice-Modelle

Ein zeitnahes Tracking auf Vollständigkeit über den Abgleich von Screening- mit Geburtenmeldungen, verbunden mit einem Handlungsschema zur Sicherstellung des NBS(-Angebots) für Kinder, die beim Abgleich als ungescreent identifiziert wurden, erfolgt z.B. in England [181], Belgien (Wallonien) [182] und den kanadischen Provinzen Alberta [183] und Ontario [184]. In diesen Ländern wird das Neugeborene unmittelbar nach der Geburt durch die geburtshilfliche Einrichtung gemeldet, dabei werden Bürger- oder Gesundheits-IDs vergeben, die nicht nur für das Tracking genutzt werden.

Ein erfolgreiches Trackingsystem ist in Bayern etabliert [22 (S.40)]: Das Tracking notwendiger Wiederholungs- und Kontrolluntersuchungen ist hier, ebenso wie das Tracking auf Vollständigkeit (Abgleich Screening- und Geburtenmeldungen), über ein öffentlich finanziertes Screeningzentrum im Öffentlichen Gesundheitsdienst vertraglich zwischen dem zuständigen Ministerium, der Kassenärztlichen Vereinigung Bayern (KVB), den Krankenkassen und den beiden bayerischen Screeninglaboren geregelt [177, 185]. Voraussetzung für das Tracking ist die Elterneinwilligung zur Datenübermittlung an das Screeningzentrum, die in über 98% erteilt wird. Durch das Tracking kann eine Abklärungsrate bei auffälligen Befunden von 99% sowie eine Teilnahmerate am Screening von knapp 99% erreicht werden [186].

In den meisten US-Bundesstaaten gibt es außerhalb der Labore koordinierende Zentren für das Neugeborenen-Screening, zu deren Aufgaben das Tracking von Kontrollkarten und auffälligen Befunden gehören, häufig durch eine*n „follow-up manager“ oder „follow-up coordinator“ [187, 188]. Zwei Staaten berichteten für das Jahr 2020 Abklärungsraten von 100% für auffällige Befunde [189]. Im Staat Kalifornien wird das Tracking unterstützt durch das zentrale, vom Screening-Programm bereitgestellte und verwaltete Tracking-Webportal „Screening Information System (SIS)“ [174], in das die Ergebnisse der Konfirmationsdiagnostik direkt von den durchführenden Einrichtungen eingegeben werden. Ein ähnliches System besteht in den Niederlanden. Dort wird der Screening-Prozess von drei Regionalzentren des staatlichen Gesundheitsinstituts RIVM koordiniert und überwacht [171]. Alle auffälligen Screeningbefunde werden über medizinische Berater*innen der RIVM-Regionalzentren in eine von RIVM bereitgestellte Datenbank übernommen. Die Ergebnisse der Konfirmationsdiagnostik werden durch die durchführende Einrichtung direkt in diese Datenbank eingegeben [176]. Die Einwilligung der Eltern zum Screening schließt die notwendige Datenübernahme ein; eine nachträgliche Ablehnung der Datenerfassung ist möglich und führt automatisch zu einer Anonymisierung der Ergebnisdaten [190].

3.6.4 Zusammenfassung

- Ein gut funktionierendes Trackingprogramm zur Sicherstellung einer hohen Teilnahmerate am NBS und der Durchführung aller notwendigen Folgeuntersuchungen ist für die Effektivität des NBS unabdingbar.
- Digitale Plattformen und ein datenschutzkonformer Austausch zwischen den Akteuren können das Tracking erleichtern.

3.6.5 Konzeptvorschläge: Tracking

Für die Sicherstellung einer möglichst hohen Teilnahme am ENS wäre ein bundesweites Trackingsystem zu etablieren und finanzieren. Jedes neugeborene Kind sollte mit dem gelben Kinderuntersuchungsheft eine einheitliche eineindeutige Screening-ID erhalten, mit der es in einer zentralen digitalen Plattform erfasst wird (s. Kapitel 3.8). Über diese Plattform könnte automatisiert geprüft werden, ob ein Screening unter dieser ID erfolgt ist. Mit standardisierten Anschreiben könnten Eltern auf fehlende Screeninguntersuchungen aufmerksam gemacht werden.

Das notwendige Tracking der Wiederholungs- und Kontrolluntersuchungen sowie der Ergebnisse der Konfirmationsdiagnostik könnte, mit entsprechender Finanzierung, über ein systematisches regionales Tracking durch Screeningzentren oder Befundkoordinator*innen in den Laboren, ggf. mit Supervision durch eine zentrale Koordinierungsstelle, vorgesehen werden.

3.7 Dokumentation und Evaluation

3.7.1 Ergebnisse der Literaturrecherche

Dokumentation und Evaluation von Screening-Programmen einschließlich Langzeitbeobachtung und gesundheitsökonomischer Aspekte [8, 31] gelten als essentiell für die kontinuierliche Qualitätsentwicklung der Programme [191]. Bei Implementierung neuer Verfahren bzw. Erweiterungen bestehender Programme wird durchgehend gefordert, Outcome-Studien von vornherein mit anzulegen [16, 32, 63, 192]. Für eine einheitliche Berichterstattung über NBS-Programme, die Beobachtung der Ergebnisse bei Neugeborenen mit bestätigten Erkrankungen und die Bewertung des Screening-Nutzens sind standardisierte Algorithmen für Strategien der Konfirmationsdiagnostik und Falldefinitionen für alle Zielkrankheiten des NBS-Programms entscheidend [30, 63, 66, 108, 162–164] (s. auch 3.5.1).

Zunächst sollten Zielparameter für die Qualitätsbewertung definiert werden, deren Zielerreichung regelmäßig überprüft und in Qualitätsberichten berichtet wird. Im Sinne des „Public Health Action Cycle" müssen aus nicht erreichten Zielen Konsequenzen folgen [28]. Dies kann mitunter auch die Überprüfung der Realisierbarkeit der Zielgröße beinhalten. Zusätzlich ist eine Evaluation des Screening-Programmes zumindest bei erheblichen Defiziten in der Zielerreichung (Qualitätsberichte) sowie nach größeren Anpassungen der Richtlinie oder der Einführung neuer Zielkrankheiten vorzusehen. Rückmeldungen zu den Ergebnissen der Konfirmationsdiagnostik von den medizinischen Einrichtungen an das Screeninglabor sind Basis für die Evaluation grundlegender Aspekte wie Prävalenz der Diagnosen und Gütekriterien der Screeningtests [32]. Die Kenntnis von Ergebnissen der Konfirmationsdiagnostik wird auch zur Qualitätssicherung und Optimierung der Analytik benötigt [26]. Die Sensitivität eines Screening-Programms kann nur über die Erfassung später diagnostizierter Fälle mit unauffälligem Screening (falsch-negatives Screening) bestimmt werden, z.B. über Patientenregister [26, 66].

Eine langfristige registerbasierte Nachbeobachtung (Long-Term Follow-up) ermöglicht die Bewertung des Nutzens des NBS für die Betroffenen, ihre Familien und die Gesellschaft [32, 66, 191, 193, 194] und kann zur Optimierung von NBS-Programmen sowie für die Harmonisierung von evidenzbasierten Empfehlungen beitragen [109, 195–197], wird aber weltweit bisher wenig durchgeführt [32, 194, 197]. Generell gelten Patientenregister bei „Seltenen Erkrankungen" als wesentlich [160]. Aufgrund der niedrigen Patientenzahlen sind vernetzte oder überregionale bzw. internationale Patientenregister am sinnvollsten [161, 197].

3.7.2 Situation in Deutschland

Die Labore haben nach §26 (2) der Kinder-Richtlinie [23] regelmäßige Qualitätsberichte abzugeben, die jährlich für alle deutschen Screeninglabore als Nationaler Screening-Report von der Deutschen Gesellschaft für Neugeborenen-Screening e. V. (DGNS) ehrenamtlich erstellt und öffentlich zugänglich publiziert werden [25]. Eine Verwendung dieses Qualitätsberichts für die Ableitung und Umsetzung kontinuierlicher qualitätssichernder Maßnahmen ist jedoch weder vorgesehen noch etabliert.

3.7.3 Best-Practice-Modelle

Definierte Indikatorensätze für die Evaluation des Screening-Prozesses und regelmäßige Qualitätsberichte gibt es z.B. in Neuseeland [198, 199], den Niederlanden [200–202], Ontario [203, 204] oder in England [205, 206]. Die erforderlichen Daten einschließlich der Ergebnisse der Konfirmationsdiagnostik werden systematisch erfasst und ausgewertet. Insbesondere zu prä-analytischen Qualitäts-Indikatoren (z.B. Versandzeiten, Qualität der Blutspots oder der übermittelten Daten) werden teilweise Berichte in kurzen Zeitabständen erstellt (z.B. vierteljährlich), auch regional oder einrichtungsbezogen. Jahresberichte enthalten sowohl Daten zu Qualitätsindikatoren wie Vollständigkeit oder Prozesszeiten als auch eine Erfassung der Fälle nach Diagnosen (auffälliges Screening, bestätigte Fälle) bzw. Angaben wie Recall-Raten, Vorhersagewerte oder Spezifität der Analysen.

Eine systematische Erfassung später detektierter Fälle mit falsch-negativem Screening ist z.B. in den Niederlanden über die Datenbank zur Konfirmationsdiagnostik [176, 190] (s auch 3.5.3) und in Schweden über das Register für angeborene Stoffwechselkrankheiten [207] etabliert (s. unten).

Besonders beeindruckende Beispiele für die Langzeitbeobachtung im Screening detektierter Patient*innen sind die Patientenregister in Schweden. Hier gibt es sowohl ein staatlich finanziertes digitales Register für angeborene Stoffwechselkrankheiten [145, 207] als auch ein nationales AGS-Register [208]. Auch in den USA gibt es verschiedene Langzeit-Follow-up-Programme, sowohl in Einzelstaaten, z.B. das kalifornische jährliche Follow-up bis zum Alter von 6 Jahren über das Tracking-Webportal SIS [174] als auch in Verbünden mehrerer Staaten [209–212]. Auf europäischer Ebene wird seit 2019 in Zusammenarbeit mit dem Europäischen Referenznetzwerk für seltene hereditäre Stoffwechselstörungen (MetabERN) das webbasierte Stoffwechsel-Register U-IMD (Unified European registry for Inherited Metabolic Diseases) vom Universitätsklinikum Heidelberg koordiniert [213, 214].

3.7.4 Zusammenfassung

- Die Dokumentation und Evaluation des Screening-Programms einschließlich Langzeit-beobachtung und gesundheitsökonomischer Aspekte gelten als essentiell für die kontinuierliche Qualitätsentwicklung des Programms. Eine Evaluation sollte insbesondere für neu aufgenommene Zielkrankheiten von Beginn an geplant werden.
- Für die Qualitätsbewertung müssen Zielparameter definiert werden, deren Zielerreichung regelmäßig überprüft wird.
- Im Sinne des „Public Health Action Cycle“ müssen aus nicht erreichten Zielen Konsequenzen folgen.
- Die Rückmeldung der Ergebnisse der Konfirmationsdiagnostik wird zur Qualitätssicherung benötigt.

3.7.5 Konzeptvorschläge: Dokumentation und Evaluation

Für die Qualitätssicherung des ENS ist eine standardisierte Dokumentation und regelmäßige Evaluation des gesamten Screening-Prozesses notwendig. Als Voraussetzung für die Evaluation müssen in der Kinder-Richtlinie Zielparameter für die Qualitätsbewertung definiert werden, deren Zielerreichung regelmäßig überprüft werden soll. Eine standardisierte Dokumentation der Ergebnisse der Konfirmationsdiagnostik sollte etabliert werden.

Regelmäßige Qualitätsberichte (z.B. DGNS-Report) müssten in der Richtlinie vorgesehen sein und finanziert werden. Darin aufgedeckte Schwächen und Qualitätsdefizite müssen im Sinne eines „Public Health Action Cycle“ innerhalb festgesetzter Fristen bearbeitet und daraus Konsequenzen wie z.B. eine weitergehende Evaluation und Maßnahmen zur Qualitätsverbesserung in den betreffenden Programmschritten gezogen werden.

Zur Evaluation des ENS und des Langzeit-Outcomes der betroffenen Kinder sollten Patientenregister in Kooperation mit den Fachgesellschaften geführt werden.

3.8 Digitalisierung im Screening-Prozess

3.8.1 Ergebnisse der Literaturrecherche

Digitale Übermittlungs- oder Abfragewege können den Screening-Prozess vereinfachen, beschleunigen und absichern. Dies gilt z.B. für die traditionell auf der Testkarte manuell eingetragenen Angaben zum Kind [127], die Nachverfolgung des Versands der Testkarte bis zum Eingang im Labor, die Übermittlung der Screeningbefunde und der Ergebnisse der Konfirmationsdiagnostik sowie die Langzeitbeobachtung bzw. Patientenregister [161, 174, 195, 212, 215]. Eine Datenplattform (Datenbank) dient dem Monitoring und der Qualitätssicherung sowie einer Evaluation des NBS [30].

3.8.2 Situation in Deutschland

Während des Screening-Prozesses müssen immer wieder Daten erfasst und übermittelt werden. Zunächst werden auf der Testkarte (Filterpapierkarte = Trockenblutkarte) Personendaten der Mutter und des Kindes, Angaben zu Schwangerschaftswoche, Geburtsgewicht, Medikamentengabe, Erst- oder Folgeuntersuchung sowie das genaue Abnahmedatum manuell eingetragen. Diese Daten werden zusammen mit einer Screening-ID im Labor in die Labordatenbank eingegeben. Die Screening-ID wird dem Einsender als Barcodeaufkleber vom kooperierenden Screeninglabor zur Verfügung gestellt und auf die Testkarte, in das gelbe Kinderuntersuchungsheft sowie in das Geburtenbuch bzw. die Patientenakte geklebt. Derzeit unterscheidet sich die Screening-ID in Format und Codierung zwischen den einzelnen Laboren und ist in vielen Regionen nicht eindeutig dem Kind, sondern dem Einsender zugeordnet. Daraus folgt, dass mitunter beim selben Kind für die Erst- und jede Folgeuntersuchung jeweils eine neue Screening-ID verwendet wird (z.B. wenn die erste Testkarte von der Geburtsklinik, die zweite Testkarte von einer pädiatrischen Praxis eingesandt wird) und eine Zusammenführung von mehreren Trockenblutkarten, selbst wenn sie ins gleiche Labor geschickt werden, nicht anhand der Screening-ID, sondern aufwändiger nur über Namen und Geburtstag des Kindes möglich ist. Eine ein-eindeutige Screening-ID, die einmalig und jedem Kind eindeutig zugeordnet ist, wird bislang nur in wenigen Bundesländern verwendet.

Die Screeningbefunde werden vom Labor schriftlich an den Einsender übermittelt, pathologische Befunde häufig telefonisch und per Fax. Über diese Befunde informiert nicht eine Pädiaterin oder ein Pädiater aus einer Praxis oder dem Konfirmationszentrum die Eltern, sondern der Einsender (z.B. die Geburtsklinik) und zwar in der Regel telefonisch. Erst danach können die pädiatrische Praxis oder das Konfirmationszentrum mit Einwilligung der Eltern die Befunde vom Screeninglabor erhalten. Auch die Befunde der Konfirmationsdiagnostik erhält das Labor nur nach Einwilligung der Eltern, und selbst dann nicht regelhaft.

Diese Abläufe sowie der Datenfluss sind nicht mehr zeitgemäß und dringend reformbedürftig. Sie sind nicht ausreichend standardisiert, aufwändig, fehleranfällig, datenschutzrechtlich problematisch und führen bei pathologischen Befunden mitunter zu kritischem Zeitverlust oder Missverständnissen.

3.8.2.1 Einzelinterviews und Gruppendiskussionen

In den Interviews und der Diskussion befürworteten die Vertreter*innen der meisten Screeninglabore eine pseudonyme, einheitliche eineindeutige Screening-ID für das Tracking auf eine hohe Teilnahmerate und das Tracking von Folgekarten bei Frühgeburt und Frühabnahme. Ein Teilnehmer eines Screeninglabors lehnte eine Zentralisierung ab, ein anderer hielt es für sinnvoller, anstatt eines zusätzlichen digitalen Systems das ENS in bestehende bzw. geplante digitale Formate wie die elektronische Gesundheitskarte oder die digitale Patientenakte zu integrieren. Einig waren sich alle, dass ein digitaler Austausch von Daten zwischen allen in der Versorgung des Kindes Beteiligten möglich sein sollte.

Aus den Zentren für die Konfirmationsdiagnostik (Behandlungszentren) kam der Wunsch nach einem digitalen System mit Erinnerungsfunktion für die Rückmeldung der Konfirmationsdiagnostik und der Möglichkeit des Trackings, um die Vollständigkeit der Konfirmationsdiagnostik zu gewährleisten. Das gewünschte System wurde an einer zentralen Stelle (z.B. DGNS, Fachgesellschaften oder Screeninglabor) gesehen. Medienbrüche (digital – Papierform – digital) sollten dadurch vermieden, die Vollständigkeit der Befunde erhöht, aber auch die Rückmeldung an das Labor beschleunigt und vereinfacht werden. Die Daten könnten in eine Gesamtdatenbank eingegeben werden, die wiederum die DGNS für die Auswertung der Daten und als Grundlage für die Erstellung der Qualitätsberichte nutzen könnte. Dies würde die Qualität der Daten verbessern und die Auswertung erleichtern. Das digitale System müsste mit den bestehenden Systemen in Zentren und Kliniken kompatibel sein und redundante Datenerfassung und andere sich wiederholende Aufgaben vermeiden. Vielmehr sollte es die Abläufe vereinheitlichen und vereinfachen. Die Bedeutung und Verwendung der Daten aus dem Screening müsste bei externen Einsendern deutlich gemacht werden, um sie zur Dateneingabe zu motivieren.

Einer Digitalisierung des Screening-Prozesses, vor allem um ein Trackingsystem aufzubauen, standen die Vertreter*innen der pädiatrischen Praxen prinzipiell offen gegenüber, wobei gewährleistet sein müsse, dass das System einfach in der Eingabe sei und zuverlässig laufe, da sonst der Aufwand zu groß sei. Den datenschutzkonformen schnellen Informationsaustausch über die verschiedenen Akteure im Screening hinweg und den Zugriff auf Befunde sahen die Pädiater*innen positiv. Sie sind auch an den Befunden der von ihnen behandelten Kinder interessiert, bei denen sie nicht selbst ein Screening abgenommen haben. Ein Arzt schlug die Einführung eines QR-Codes vor, über den Zugriff auf Befunde, auch für weitere Screening-Untersuchungen, zentral ermöglicht würde.

3.8.3 Best-Practice-Modelle

Beispiele für die Nutzung digitaler Wege der Datenübermittlung oder -erfassung im Screening-Prozess finden sich in vielen Ländern. Ein Personen-ID-Code wird im Screening-Prozess z.B. in England [181], Belgien [182], den kanadischen Provinzen Alberta [183] und Ontario [184], den Niederlanden [216], Polen [217], Dänemark [218], Finnland [152], Slowenien [219] und Ungarn [220] verwendet. In Dänemark [218], Finnland [152], Slowenien [219] und Ungarn [220] werden die erforderlichen Daten zur Testkarte (Personendaten, klinische Angaben wie Geschlecht, Geburtsgewicht, Gestationsalter)

mit der entsprechenden ID von der einsendenden Einrichtung an das Screeninglabor digital übermittelt. Kalifornien, Ontario, Österreich und Polen ermöglichen eine webbasierte digitale Eingangskontrolle der Testkarte im Labor: Kalifornien und Ontario über Versand-Tracking-Tools für die einsendenden Einrichtungen [221, 222], Österreich und Polen über eine allgemein zugängliche Abfragemaske zum Probeneingang (über die Screeningnummer) [217, 223]. Digitale Mitteilungs- oder Abfragemöglichkeiten für unauffällige Befunde stellen beispielsweise Finnland [152], Israel [153] und Portugal [154] für Eltern zur Verfügung. Die Übermittlung der Screeningbefunde an die zuständige Stelle erfolgt z.B. in Dänemark [218], Finnland [152], Schweden [224] und Slowenien [219] digital, in Ontario können die Befunde von Ärzten oder Ärztinnen über das digitale Labor-Informationssystem abgerufen werden [225, 226]. In den USA hat die Initiative „NewSteps" der APHL einen Leitfaden zur Unterstützung von NBS-Programmen bei Implementierung und Pflege von IT-Systemen für den Datenaustausch zwischen Einsendern und Screeninglaboren herausgegeben, der Lösungen für IT, Zugangsverwaltung, Datenschutz, Authentifizierung etc. darstellt [227]. Derartige IT-Systeme wurden z.B. in den US-Bundesstaaten Nevada, Louisiana, Texas und Utah in den letzten Jahren implementiert [189, 228, 229]. Vom Screening-Programm bereitgestellte Tracking-Datenbanken mit Web-Tools, in die die Ergebnisse der Konfirmationsdiagnostik direkt von der durchführenden Klinik eingegeben werden, gibt es z.B. in Kalifornien [174] und in den Niederlanden [176]. Das kalifornische Tracking-Webportal wird zusätzlich auch für ein jährliches Follow-up der detektierten Kinder bis zum Alter von sechs Jahren über direkte Eingaben durch die Kliniken genutzt [174, 175]. Weitere Langzeit-Follow-up-Datenbanken mit Web-Tools aus den USA sind z.B. die Datenbank "Longitudinal Pediatric Data Resource" (LPDR) des Newborn Screening Translational Research Network (NBSTRN) [212] oder das digitale Instrument "Digital Health Footprint" für Stoffwechselpatient*innen aus Georgia [215]. Letzteres soll sowohl der lebenslangen Sicherstellung von Therapie als auch der Outcome-Evaluation dienen. Das schwedische staatlich finanzierte digitale Register für angeborene Stoffwechselkrankheiten [145, 207] schöpft zahlreiche Nutzungsmöglichkeiten moderner IT aus. So werden beispielsweise die Daten aus der Konfirmations-Datenbank des Screeninglabors ebenso wie laufende Laborergebnisse direkt in das Register übertragen [224]. Das schwedische Register bietet Zugangsmöglichkeiten nicht nur für medizinisches Fachpersonal, sondern zu bestimmten Bereichen (z.B. Laborergebnisse, Fragebögen) auch für Patient*innen bzw. Familien [145].

3.8.4 Zusammenfassung

Eine zentrale digitale Plattform kann die Sicherstellung einer hohen Teilnahmerate und ein Tracking der auffälligen und wiederholungsbedürftigen Befunde auch laborübergreifend und datenschutzkonform vereinfachen. Auch die Rückmeldung der Konfirmationsdiagnostik an das Screeninglabor kann über eine digitale Plattform erleichtert werden. Wichtig sind dabei die digitale Dokumentation des gesamten Screening-Prozesses und ein eineindeutiger Schlüssel für jedes Neugeborene. Diese zentrale Datenbank stellt zugleich die Datengrundlage für Qualitätsberichte und Evaluation dar.

3.8.5 Konzeptvorschläge: Digitalisierung

Während des Screening-Prozesses müssen laufend Daten dokumentiert und übermittelt werden. Eine Digitalisierung ist hierfür unumgänglich. Zu diesem Zweck wird die Etablierung einer zentralen bundesweiten digitalen Plattform auf einem sicheren Server mit rollenbasierter Zugriffskontrolle und einem datenschutzkonformen Schlüsselkonzept empfohlen.

Das Konzept könnte folgendermaßen aussehen: Die Plattform enthält pseudonyme Daten aus dem gesamten Screening-Prozess. Als Pseudonym wird jedem Neugeborenen mit der Ausgabe des gelben Kinderuntersuchungsheftes durch den jeweiligen Kreißsaal bzw. bei nicht stationärer Geburt durch die Hebamme eine eineindeutige Screening-ID über Barcode-Etiketten zugeordnet. Diese Screening-ID muss für jedes Neugeborene kurz nach der Geburt zusammen mit einer Einsenderkennung in die zentrale Plattform eingegeben werden. Für einen evtl. notwendigen Kontakt mit den Eltern werden die Personendaten der Mutter und des Neugeborenen, nach Möglichkeit elektronisch aus dem Krankenhausinformationssystem (KIS), in einen gesonderten, extra gesicherten Bereich der zentralen Plattform eingelesen. Eine Zuordnung dieser Daten zu der Screening-ID kann nur über eine (ggf. digitale) Vertrauensstelle erfolgen und ist für ca. 98 % der Neugeborenen nicht erforderlich, da bei ihnen ein unauffälliger Screeningbefund vorliegt.

Für jede Untersuchung im Verlauf des Screening-Prozesses (erste Testkarte, Folgekarten, Konfirmationsdiagnostik) wird die eineindeutige Screening-ID des Neugeborenen verwendet und ein Barcode-Etikett z.B. auf die Testkarte, in das Geburtenbuch oder die Patientenakte und auf die Dokumentationsseite für das ENS im gelben Kinderuntersuchungsheft geklebt. Der Einsender jeder Testkarte gibt zusätzlich notwendige Informationen wie Abnahmezeit, Besonderheiten und die Kennung des Einsenders in die Plattform ein. Das Labor ergänzt (nach Eingang der Testkarte) in der Plattform die Laborkennung, den Eingang der Karte und das Ergebnis des Screenings. Ein Barcode kann auch für alle weiteren Screenings (z.B. Hörscreening) verwendet werden. Dieselbe eineindeutige Screening-ID wird auch bei einer notwendigen Konfirmationsdiagnostik verwendet. Das Konfirmationszentrum ergänzt die Kennung des Zentrums und die wichtigsten Parameter der Konfirmationsdiagnostik sowie die endgültige Diagnose. Auch später diagnostizierte im Screening unauffällige Fälle müssen unter der entsprechenden Screening-ID in die Plattform eingegeben werden.

Die Einrichtung einer zentralen Daten-Plattform bietet folgende Vorteile:

- Das Tracking fehlender Screeninguntersuchungen kann automatisiert erfolgen, indem den eineindeutigen Screening-IDs geborener Kinder die in den Laboren eingegangenen Testkarten zugeordnet und standardisierte Anschreiben an Eltern nicht gescreenter Kinder erzeugt werden. Eine Verknüpfung mit Personendaten wäre nur dann vorgesehen, wenn ein Anschreiben nötig ist, und würde z.B. über eine Vertrauensstelle abgewickelt.
- Eltern können über die Screening-ID und, je nach Datenschutzerfordernis, einen zweiten Schlüssel den Eingang der Testkarte und einen unauffälligen Befund einsehen. Dies ist ebenso für den Einsender und den betreuenden Kinderarzt bzw. die Kinderärztin möglich.
- Der Kinderarzt oder die Kinderärztin kann bei der U2, ggf. U3, das Screening über die Plattform überprüfen. Ist keine Karte in einem Labor eingegangen, so sollte das Screening nachgeholt werden, bei noch offenem Befund sollte der Grund überprüft werden (fehlende Folgekarte, noch nicht abgeklärter auffälliger Befund).
  ➔ Dies unterstützt ein Tracking auf eine hohe Teilnahmerate und der kontrollbedürftigen Befunde.
- Das Konfirmationszentrum (Behandlungszentrum) oder der Kinderarzt bzw. die Kinderärztin kann über die Screening-ID und ggf. einen weiteren Schlüssel sowie entsprechende Zugriffsrechte (z.B. rollenbasierte Zwei-Faktoren-Authentifizierung) direkt den für die Diagnostik notwendigen auffälligen Screeningbefund einsehen.
- Das Labor kann sehen, ob eine notwendige Kontrolluntersuchung durchgeführt wurde oder das Kind im Zentrum angekommen ist, und kann das Ergebnis der Konfirmationsdiagnostik zur

Qualitätssicherung heranziehen.
➔ Dies unterstützt ein Tracking der kontrollbedürftigen Befunde.
- Der pseudonyme Screeningdatensatz kann über eine Schnittstelle in die krankheitsspezifischen Register der Fachgesellschaften eingespielt werden und als Basisdatensatz die Vollständigkeit des Registers anzeigen und möglicherweise verbessern.
- Der Qualitätsbericht kann zu großen Teilen aus der Auswertung der anonymisierten Daten der Plattform erstellt werden, was die Datenqualität verbessern und den Arbeitsaufwand der Labore verringern dürfte.

3.9 Zentrale Koordination

3.9.1 Ergebnisse der Literaturrecherche

Da beim NBS die gesamte Population überwiegend gesunder Neugeborener ohne erhöhtes Risiko für bestimmte Erkrankungen untersucht wird, sind an die Prozessqualität im analytischen sowie prä- und postanalytischen Screening-Ablauf besonders hohe Anforderungen zu stellen [9] (s. auch Kapitel 1, 3.6).

Das Neugeborenen-Screening als Public-Health-Aufgabe [6, 56, 230] erfordert ein laufendes Qualitätsmanagement im Sinne eines „Public Health Action Cycle“ [28] (Abbildung 14). Dabei ist eine kontinuierliche Qualitätssicherung entscheidend für das Erreichen einer hohen Effektivität und Effizienz des Screening-Programms sowie die Minimierung potenzieller Risiken des Screenings [16, 22]. Dies erfordert sowohl ein Qualitätsmanagement der einzelnen Programm-Elemente und -Prozesse als auch die Evaluation des gesamten Programms inklusive der Zielkrankheiten (Re-Evaluation eingeschlossener Zielkrankheiten auch im Hinblick auf die klinische Relevanz milder Formen). Optimale Screeningergebnisse können nur bei sorgfältiger Organisation und Steuerung des gesamten Screeningpfads von der Aufklärung bis zur Therapie [7, 8, 10, 22, 63] erzielt werden. Qualitätsmanagement und Evaluation werden häufig im Vorfeld der Implementierung eines Screenings nicht genug beachtet, obwohl sie für den Erfolg essentiell sind [62]. Die WHO fordert, dass alle für ein Screening-Programm erforderlichen Komponenten angemessen finanziert werden. Dies ist von entscheidender Bedeutung insbesondere für die Programmkoordinierung, Erstellung von Leitlinien und Protokollen sowie die Auswertung der Programme, die Qualitätssicherung und Datenanalyse. Alle diese Komponenten sind als „entscheidende Hilfsfunktionen“ für die Effektivität des Screening-Programms maßgeblich [22].

In vielen Publikationen wird betont, dass das NBS ein Programm und nicht nur ein Test ist [7, 8, 10, 22, 63, 231]. Andermann unterstreicht in den überarbeiteten Screening-Kriterien: „Kein Screening ohne ein Screening-Programm“; „Screening ist mehr als ein Test“ [16]. Sie fordert in einem Vortrag ein integriertes Screening-Programm, das die Ebenen Aufklärung, Tests, klinische Dienste und Programmverwaltung umfasst und nennt dafür folgende drei Koordinationsebenen [232]:
- Programm-Management mit Aufsicht, Ressourcenmanagement, Organisation der Dienste, Qualitätsmanagement usw.
- Klinik mit Aufklärung, Einwilligung, Screening-Durchführung, Befundmitteilung, Konfirmationsdiagnostik, Follow-up
- Screeninglabor mit Analytik, Befundung und Qualitätssicherung

3.9.2 Situation in Deutschland

Die Grundsätze, die Durchführung und Laboranalytik des Screenings sind in der Kinder-Richtlinie geregelt, allerdings nicht im Sinne eines Screening-Programmes. Ein Screening-Programm sollte – wie von vielen Autoren, der EU und der WHO gefordert – alle Prozessschritte von der Rekrutierung über die Elterninformation bis zum Therapiebeginn umfassen sowie eine zentrale Koordination und ein Qualitätsmanagement enthalten. Die Regelungen der Richtlinie enden mit dem Screeningbefund, eine zentrale Koordination des Screening-Prozesses als Screening-Programm ist nicht vorgesehen.

Die Ergebnisse des Modellprojektes der GKV „Erweitertes Neugeborenen-Screening in Bayern“ (1999-2005) waren neben den Empfehlungen der Screening-Kommission der DGKJ die Grundlage für die Einführung des ENS in Deutschland [233]. Diesem Modellprojekt lag das Konzept eines „public private partnership“ zugrunde. Ein Screeningzentrum im Öffentlichen Gesundheitsdienst (ÖGD) übernahm als zentrale Koordinierungsstelle die Sicherstellung einer hohen Prozessqualität z.B. durch Tracking und die Evaluation des bayerischen Screening-Programms. Dieses Konzept hatte sich sehr bewährt [185, 234]. In der Folge wurde die Einrichtung von Screeningzentren von der Screening-Kommission der DGKJ empfohlen [235]. Bei der flächendeckenden Einführung des ENS im Jahr 2005 in Deutschland wurde die Empfehlung jedoch nicht berücksichtigt.

Das ENS wurde 2005 für zehn Stoffwechselkrankheiten und zwei Hormonstörungen und damit für nur zwei verschiedene Krankheitsentitäten eingeführt. Mittlerweile sind mit Mukoviszidose, SCID (Immundefekte), Sichelzellkrankheit (Hämatologie) und SMA (Neurologie) vier weitere Krankheiten aus jeweils eigenen Entitäten hinzugekommen. Zukünftige Erweiterungen des Screeningpanels werden voraussichtlich noch andere Fachbereiche betreffen. Dadurch ist das NBS seit seiner Einführung in verschiedenen Hinsichten stetig komplexer geworden. Dies betrifft neben der Laboranalytik und deren Befundung auch die Herausforderungen aufgrund vielfältigerer Verantwortlichkeits- und Organisationsstrukturen. Diese Komplexität muss auf Programmebene transparent reflektiert werden, weil ansonsten organisatorische und medizinische Probleme zu erwarten sind. Durch eine institutionalisierte zentrale Koordination könnte das NBS der Komplexität gerecht werden.

3.9.2.1 Auswertung der DGNS-Daten 2006 bis 2019

Die Auswertung der DGNS-Daten zeigt deutliche Unterschiede zwischen den Laboren (s. auch Kapitel 3.3.2.2). Dies betrifft die Recall-Raten (Abbildung 7, Abbildung 9), die Prozesszeiten (Abbildung 10, Abbildung 12) sowie die Rate an Loss to follow-up (Abbildung 11).

Abbildung 12 zeigt die Zeitdauer, die die Testkarten von der Blutabnahme bis zum Eingang im Labor benötigen. Sie hat im Laufe der Jahre deutlich zugenommen (Abbildung 13) und liegt 2019 in 30% der Fälle bei mehr als 3 Tagen, wobei regional große Unterschiede bestehen (5,24 % bis 47,85 %, s. Anhang 1, Abbildung 8). Ob lange Zeiten durch Organisationsschwierigkeiten beim Einsender oder lange Postlaufzeiten bedingt sind, ist aus den Daten nicht ersichtlich.

Auch die Anzahl der Testkarten, die wegen mangelhafter Qualität (schlecht oder nicht ausreichend mit Blut betropft) zur Anforderung von Folgekarten führt, unterscheidet sich zwischen den Laboren erheblich (Spannweite der Mittelwerte 0,04 % bis 0,98 %, s. Anhang 1, Abbildung 12) und ist mit Einführung des CF-Screenings insgesamt angestiegen.

Aus der Kenntnis dieser Qualitätsunterschiede müssten (auch im Sinne des „Public Health Action Cycle“) Konsequenzen im Sinne einer Qualitätsanpassung gezogen werden. Dies geschieht bislang nicht.

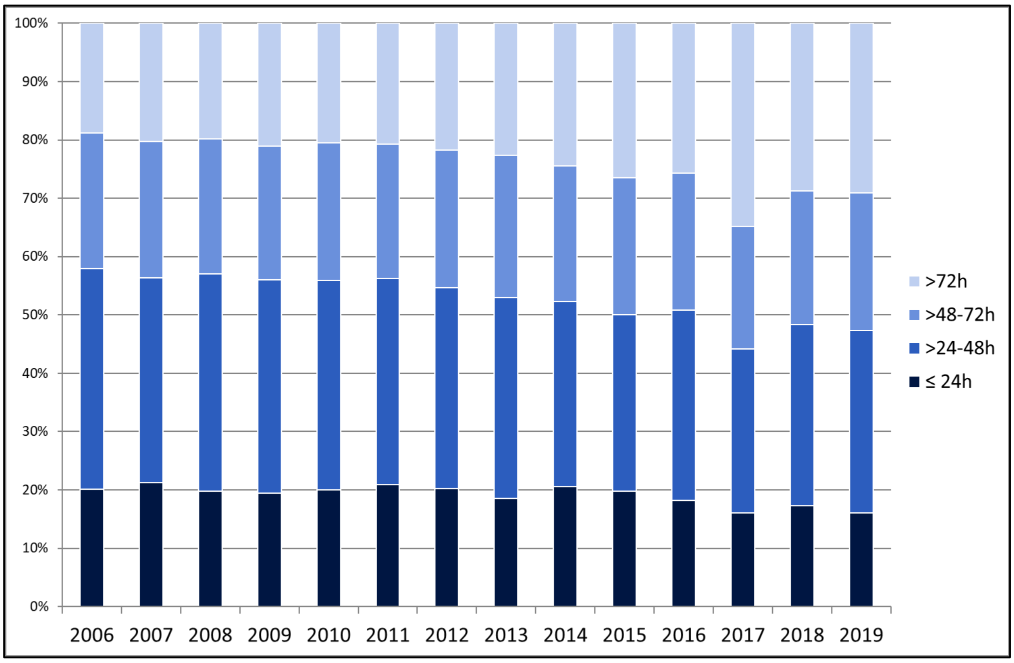

Abbildung 12 Zeitraum von der Blutabnahme bis zum Eingang im Labor

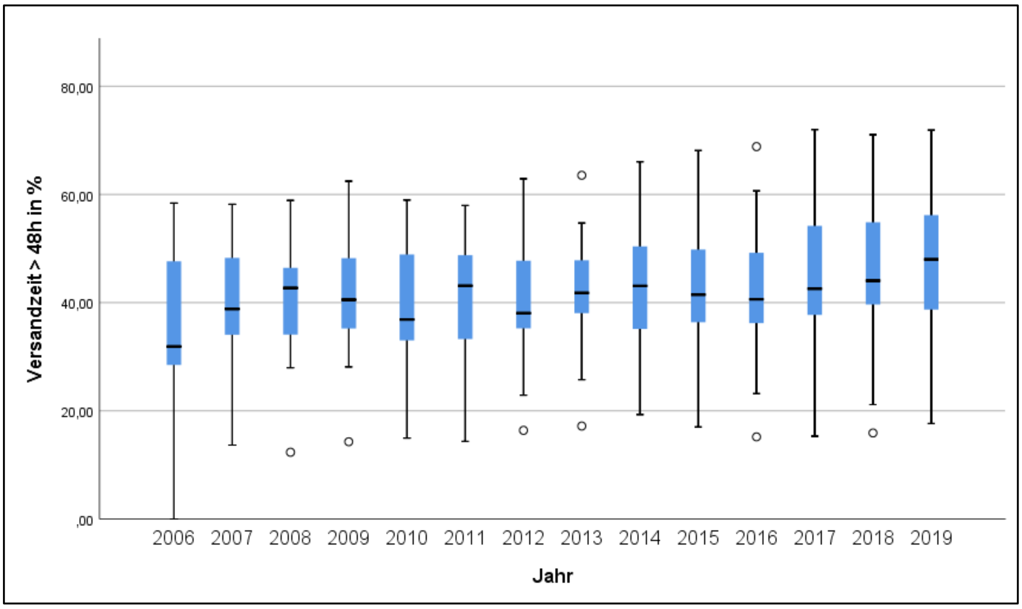

Abbildung 13 Spannweite der Versandzeiten zwischen den Laboren

3.9.2.2 Online-Befragung von Vertreter*innen der Geburtskliniken

in der Online-Befragung gaben nur 41,2% der Teilnehmenden einen Versand „immer innerhalb von 24 Stunden nach Blutentnahme“ an, 50,5% einen Versand „innerhalb von 24 Stunden nach Abnahme außer an Wochenenden und Feiertagen“ und 1,5% einen Versand „an festgelegten Wochentagen“ oder „2-3x pro Woche“. 67,5 % der Einrichtung versenden die Testkarten mit der Deutschen Post an das zuständige Labor, 23,7 % mit einem Kurierdienst der Klinik oder des Labors, 3,1% über andere Zusteller und 9,8 % haben die Frage nicht beantwortet.

3.9.2.3 Einzelinterviews und Gruppendiskussionen

Einige Interviewpartner*innen gaben explizit an, dass das ENS insgesamt gut funktioniere.
Dennoch wurden von den Einsendern Probleme z.B. bei Datenaustausch und Tracking benannt. Zur Verbesserung wurden insbesondere zentrale digitale Lösungen und eine zentrale koordinierende Stelle angeregt. Die Vertreter*innen der Labore sahen wenig Handlungsbedarf in ihrem Bereich, kritisierten aber mangelnde Koordination anderer Elemente des Screening-Prozesses in Deutschland (Beispiele: Aufklärung und Schulung der Einsender, Bereitstellung einer zentralen Internetseite mit Informationen für Eltern und Einsender, Erreichen aller Neugeborenen, Tracking, zeitgerechte Sicherstellung der Konfirmationsdiagnostik).

Teile des ENS-Prozesses, die in einem System von Anfang bis Ende koordiniert sein sollten, funktionierten in Deutschland nur durch das Engagement einzelner Akteure. So werde das Potential guter Laborleistungen in Deutschland aufgrund mangelhafter Qualität anderer Elemente des Screening-Prozesses nicht ausgeschöpft. Das Ziel des ENS – eine schnelle und kompetente Behandlung betroffener Neugeborener – werde teilweise durch Vorgaben der Kinder-Richtlinie, des GenDG und des Datenschutzes erschwert. Kritisiert wurde auch, dass aus dem jährlichen DGNS-Report mit seinen Screeningergebnissen kaum Konsequenzen gezogen und umgesetzt werden.
Unter den Expert*innen für die Konfirmationsdiagnostik bestand Konsens darüber, dass das Neugeborenen-Screening ein guter, schneller und recht sicherer Prozess sei. Jedoch sahen die Expert*innen im Screening-Prozess bei der Befundmitteilung und dem Zugang der Familien zum Zentrum sowie der Rückmeldung der Konfirmationsdiagnostik an das Screeninglabor Probleme, die eine übergeordnete Koordination erfordern.

Die Vertreter*innen der Labore kritisierten die Finanzierung des ENS aus der DRG-Geburtenpauschale und fehlende Anpassungen der DRG-Pauschalen bei ENS-Erweiterungen. Es fehle eine klare Regelung der Vergütung im stationären Bereich, die das ENS als gesonderte Leistung anerkennt. Andere Gruppen forderten Vergütungsregelungen auch für Aufklärung, Tracking, Befundmitteilung und Rückmeldung der Konfirmationsdiagnostik.

3.9.3 Best-Practice-Modelle

In einigen Ländern mit regionalisiertem NBS wurden in den letzten Jahren zentrale Koordinierungsstellen eingerichtet, z.B. in Italien [236, 237] oder Frankreich [238]. Die Grundlagen eines einheitlichen qualitätsgesicherten NBS sind im UK durch das National Screening Committee (NSC) [239, 240], in Australien durch den nationalen Rahmenleitplan „Newborn Bloodspot Screening – National Policy Framework" [241] staatlich geregelt. Die Umsetzung erfolgt in den einzelnen Provinzen bzw. Landesteilen entsprechend dieser Vorgaben. Auch die NBS-Programme in Neuseeland [242], Norwegen [40] und Ontario [151] sind gute Beispiele für zentral geregelte und koordinierte NBS-Programme mit laufendem Qualitätsmanagement. In diesen Ländern werden allerdings alle Screeningproben in nur einem Screeninglabor analysiert, so dass einige für die deutsche Situation besonders relevante Koordinationsprobleme entfallen. Beispiele für Korrekturmaßnahmen von Schwachstellen, die bei Evaluationen auffielen, sind die Einführung eines geänderten Follow-up-Protokolls zur Verkürzung der Abklärungszeiten nach grenzwertig auffälligen Befunden in Neuseeland [243] oder die Einführung von E-Learning-Tools und Online-Workshops für Einsender sowie des ausschließlichen Versands der Trockenblutkarten über einen Kurierdienst mit Trackingsystem zur Qualitätsverbesserung der Prä-Analytik in Ontario [244].

Als Best-Practice-Beispiel für ein NBS-Programm mit zentraler Koordination und Qualitätssicherung, mehreren Screeninglaboren und regionalen Screeningzentren wird im Folgenden das NBS-Programm der Niederlande kurz dargestellt [245, 246]. In den Niederlanden unterliegt das NBS dem Bevölkerungsscreeninggesetz [247] und ist Teil des nationalen Bevölkerungsscreening-Programms [248]. Es wird durch das Gesundheitsministerium über den Staatshaushalt finanziert und vom Staatsinstitut für öffentliche Gesundheit und Umwelt RIVM (Rijksinstituut voor Volksgezondheid en Milieu) koordiniert und organisiert [249]. Das nationale Zentrum für Bevölkerungsscreening des RIVM ist zuständig für Rahmenkonzept, Koordinierung, Kommunikation, Informationsmanagement (z.B. Webseiten), Organisation, Aufsicht und Qualitätskontrolle des NBS auf nationaler Ebene [249]. Dabei erfolgt eine kontinuierliche Beratung durch Programmausschüsse aus Experten der relevanten Berufsgruppen und Verbände (Gesundheitsdienste, Hebammen, Hausärzte, Geburtshilfe, Genetik, Spezialist*innen, Eltern- und Patientenorganisationen) mit Arbeitsgruppen zu verschiedenen Themenbereichen [250]. Das staatliche Labor des RIVM ist eines von insgesamt fünf NBS-Laboren und gleichzeitig als nationales NBS-Referenzlabor verantwortlich für die einheitliche Durchführung und Qualitätssicherung der NBS-Analytik und -Befundung [129]. Die praktische Organisation, Umsetzung und Koordination des NBS, inklusive Tracking, wird unter Verwendung der national bereitgestellten IT-Systeme von drei Regionalbüros des RIVM übernommen; das Vorgehen bei auffälligen Befunden wird von medizinischen Berater*innen dieser Regionalbüros koordiniert [251]. Die Qualitätssicherung erfolgt auf Basis regelmäßiger Qualitätsberichte (z.B. [201]) über entsprechende Gremien des RIVM, die auch das Ministerium zu Entwicklungen des NBS, die Maßnahmen oder politische Änderungen erfordern, informieren und beraten [249].

3.9.4 Zusammenfassung

- Das ENS muss als integriertes Screening-Programm begriffen und geregelt werden, indem eine enge Vernetzung aller in das ENS eingebundenen medizinischen Einrichtungen mit festgelegten Zuständigkeiten und Prozeduren, ein Trackingsystem für eine hohe Teilnahmerate am ENS und die notwendigen Wiederholungs- und Kontrolluntersuchungen sowie die verpflichtende Rückmeldung der Konfirmationsdiagnostik an das Labor enthalten sein sollten.
- Eine zentrale Koordination und Qualitätssicherung des gesamten Screening-Programmes ist für das Erreichen einer hohen Effizienz und die Minimierung von Risiken durch das Screening entscheidend.
- Im Sinne einer kontinuierlichen Qualitätsentwicklung müssen Konsequenzen aus den Ergebnissen im jährlichen DGNS-Report und ggf. weiterer Qualitätsüberprüfungen (Evaluation) zentral festgelegt und überprüft werden („Public Health Action Cycle“, Abbildung 14).
- Umfassende gesicherte Regelungen für die Finanzierung aller Komponenten des Screening-Prozesses sind notwendig.

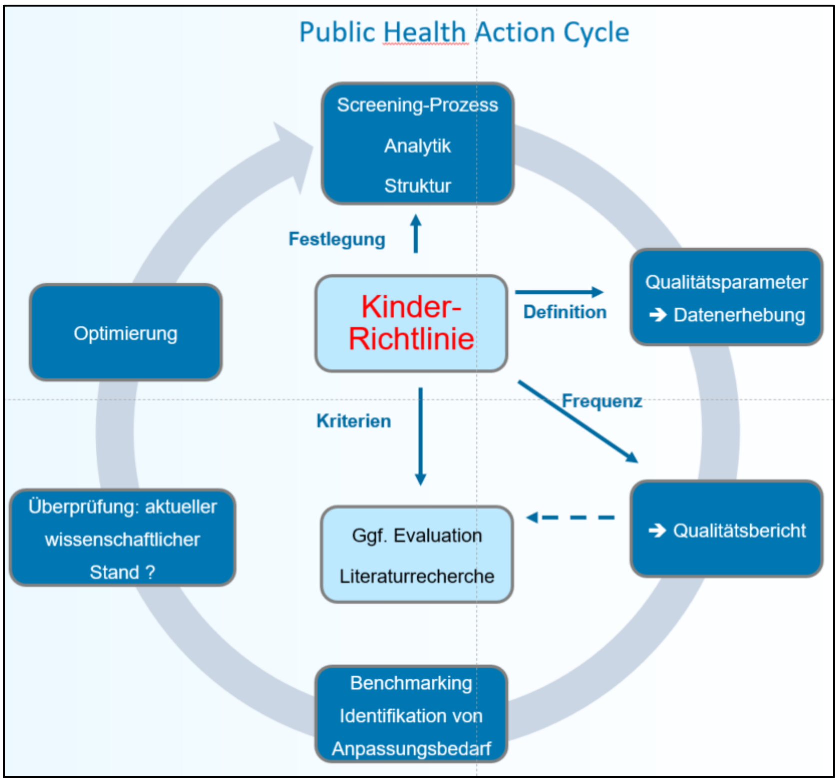

Abbildung 14 „Public Health Action Cycle"

3.9.5 Konzeptvorschläge: Zentrale Koordinierungsstelle

Zur Programmkoordination und Qualitätssicherung des ENS in Deutschland wird die Einrichtung einer zentralen Koordinierungsstelle vorgeschlagen. Diese könnte an einer öffentlichen (Public-Health) Institution angesiedelt werden, die eine Infrastruktur für die genannten Aufgaben zur Verfügung stellen kann, z.B. Robert Koch-Institut, das geplante Public-Health-Bundesinstitut oder eines der Landesämter für Gesundheit. Folgende Qualifikationen wären bei Mitarbeiter*innen wertvoll: Arzt oder Ärztin, Epidemiologie, Labormedizin oder klinische Chemie, Informatik, medizinische Dokumentation, evtl. Kommunikation.

Folgende Aufgaben könnte die Koordinierungsstelle übernehmen:

1. Öffentlichkeitsarbeit:
    - Erstellen und Pflegen einer Website für Eltern und Mitarbeiter*innen im Gesundheitssystem (verschiedene Professionen) mit einem internen Bereich für Screeninglabore und ggf. Behandlungszentren
    - Erstellen und Aktualisieren von Informationsmaterialien für Eltern (auch in Fremdsprachen / Bereitstellen von Videos etc.)
    - Pressearbeit, wenn nötig (z.B. neue Zielkrankheiten / Abläufe / nachlassende Vollständigkeit)
    - Koordinierte Aufklärungsarbeit zur Bewusstseinsbildung für die Bedeutung des ENS
    - Ansprechpartner:
        - G-BA / Politik / Stakeholder / (werdende) Eltern / Öffentlichkeit
        - Beantwortung von Fragen bezüglich der spezialisierten Zentren für die Konfirmationsdiagnostik (Behandlungszentren)

2. Lotsenfunktion und zentrales Qualitätsmanagement des gesamten ENS-Prozesses als Public-Health-Aufgabe:
    - Bereitstellung von Schulungsmaterialien für alle am ENS-Prozess Beteiligten sowie auf den besten verfügbaren Erkenntnissen basierende Handlungsleitfäden (SOPs) für jeden Schritt des Screening-Programms, die in Kooperation mit entsprechenden Expert*innen bzw. Fachgremien erarbeitet und regelmäßig angepasst werden
    - Regelmäßige Analyse der Qualitätsberichte
        - Darstellung der erreichten Zielparameter und Diskussion von Optimierungsbedarf und -möglichkeiten mit den zuständigen Professionen
        - Beschreibung notwendiger Konsequenzen bei identifizierten Qualitätsunterschieden und -mängeln
    - Koordination von Laborworkshops zur Qualitätssicherung und Diskussion zwischen den Laboren z.B. zu neuen Zielkrankheiten oder Analysemethoden, zu Beteiligung an Ringversuchen und post-analytischen multivariaten digitalen Interpretationswerkzeugen
    - Koordination und Organisation von Qualitätszirkeln aus Vertreter*innen der Labore, behandelnden Ärzten und Ärztinnen (Expert*innen) und ggf. weiteren Stakeholdern mit Einbeziehung von Eltern-/Patientenvertreter*innen zur Erarbeitung oder Anpassung von Vorschlägen für Qualitätsziele des ENS inklusive der benötigten personellen und finanziellen Ressourcen
    - Koordination eines Expertengremiums zur Bewertung möglicher neuer Zielkrankheiten, die dem G-BA zur Beratung vorgeschlagen werden
    - Regelmäßige Evaluation des gesamten Screening-Programms mit Überprüfung (Re-Evaluation) und ggf. Empfehlungen zu notwendigen Anpassungen der Kinder-Richtlinie an den aktuellen wissenschaftlichen Stand (z.B. Zielkrankheiten, Screening-Algorithmen, notwendige Kontrolluntersuchungen)

3. Digitalisierung und Verwaltung einer bundesweiten zentralen Datenplattform:
    - Betreuung und Aktualisierung der Plattform und Supervision der Anwender
    - Zentrale Verwaltung einer einheitlichen und eineindeutigen Screening-ID für jedes Neugeborene mit Barcodeklebern im Kinderuntersuchungsheft
    - Automatisiertes Tracking auf Vollständigkeit über die eineindeutige Screening-ID
    - Supervision des Trackings notwendiger Wiederholungsuntersuchungen und der Konfirmationsdiagnostik, das von den Laboren oder regionalen Screeningzentren über die digitale Plattform übernommen wird
    - Beantwortung von Fragen zu Datenbankschnittstellen zu Krankheitsregistern der Fachgesellschaften

4. Einbindung weiterer Screening-Programme (z.B. Neugeborenen-Hörscreening)

4 Literaturverzeichnis

[1] Hoffmann GF, Lindner M, Loeber JG. 50 years of newborn screening. J Inherit Metab Dis 2014; 37: 163–164. doi:10.1007/s10545-014-9688-5

[2] Gramer G, Nennstiel-Ratzel U, Hoffmann GF. 50 Jahre Neugeborenenscreening in Deutschland [[50 years of newborn screening in Germany]]. Monatsschr Kinderheilkd 2018; 166: 987–993. doi:10.1007/s00112-017-0355-4

[3] Dikow N, Ditzen B, Kölker S, et al. From newborn screening to genomic medicine: challenges and suggestions on how to incorporate genomic newborn screening in public health programs. medgen 2022; 34: 13–20. doi:10.1515/medgen-2022-2113

[4] Nennstiel-Ratzel U, Lüders A, Blankenstein O. Neugeborenenscreening: ein Paradebeispiel für effektive Sekundärprävention. Bundesgesundheitsblatt Gesundheitsforschung Gesundheitsschutz 2015; 58: 139–145. doi:10.1007/s00103-014-2092-3

[5] Deutsche Gesellschaft für Neugeborenenscreening e.V. (DGNS). Nationaler Screeningreport Deutschland 2019. Im Internet: https://www.screening-dgns.de/reports.php

[6] Marsden D, Larson C, Levy HL. Newborn screening for metabolic disorders. J Pediatr 2006; 148: 577–584. doi:10.1016/j.jpeds.2005.12.021

[7] Boyle CA, Bocchini JA JR, Kelly J. Reflections on 50 years of newborn screening. Pediatrics 2014; 133: 961–963. doi:10.1542/peds.2013-3658

[8] Cornel M, Rigter T, Weinreich S, Burgard P, Hoffmann GF, Lindner M, Loeber JG, Rupp K, Taruscio D, Vittozzi L. Newborn screening in Europe. Expert Opinion Document [**2011**] (2011). Im Internet: https://www.isns-neoscreening.org/guidelines/; Stand: 15.10.2021

[9] Gramer G, Hoffmann GF, Nennstiel-Ratzel U. Das erweiterte Neugeborenenscreening. Wiesbaden: Springer Fachmedien Wiesbaden; 2015. doi:10.1007/978-3-658-10493-1

[10] González-Irazabal Y, Hernandez de Abajo G, Martínez-Morillo E. Identifying and overcoming barriers to harmonize newborn screening programs through consensus strategies. Crit Rev Clin Lab Sci 2021; 58: 29–48. doi:10.1080/10408363.2020.1781778

[11] Liebl B, Kries Rv, Nennstiel-Ratzel U, et al. Ethisch-rechtliche Aspekte des Neugeborenenscreenings. Monatsschrift Kinderheilkunde 2001; 149: 1326–1335

[12] Gray JAM, Patnick J, Blanks RG. Maximising benefit and minimising harm of screening. BMJ 2008; 336: 480–483. doi:10.1136/bmj.39470.643218.94

[13] Wilcken B. Expanded newborn screening: reducing harm, assessing benefit. J Inherit Metab Dis 2010; 33: S205-10. doi:10.1007/s10545-010-9106-6

[14] Nicholls SG, Wilson BJ, Etchegary H, et al. Benefits and burdens of newborn screening: public understanding and decision-making. Personalized Medicine 2014; 11: 593–607. doi:10.2217/pme.14.46

[15] Goldenberg AJ, Comeau AM, Grosse SD, et al. Evaluating Harms in the Assessment of Net Benefit: A Framework for Newborn Screening Condition Review. Matern Child Health J 2016; 20: 693–700. doi:10.1007/s10995-015-1869-9

[16] Andermann A, Blancquaert I, Beauchamp S, et al. Revisiting Wilson and Jungner in the genomic age: a review of screening criteria over the past 40 years. Bull World Health Organ 2008; 86: 317–319. doi:10.2471/blt.07.050112

[17] Andermann A, Blancquaert I, Beauchamp S, et al. Guiding policy decisions for genetic screening: developing a systematic and transparent approach. Public Health Genomics 2011; 14: 9–16. doi:10.1159/000272898

[18] Wilson J, Jungner G. PRINCIPLES AND PRACTICE OF SCREENING FOR DISEASE [WHO **1968**]. Im Internet: https://apps.who.int/iris/handle/10665/37650; Stand: 11.11.2021

[19] Ombrone D, Giocaliere E, Forni G, et al. Expanded newborn screening by mass spectrometry: New tests, future perspectives. Mass Spectrom Rev 2016; 35: 71–84. doi:10.1002/mas.21463

[20] Jansen ME, Lister KJ, van Kranen HJ, et al. Policy Making in Newborn Screening Needs a Structured and Transparent Approach. Front Public Health 2017; 5: 53. doi:10.3389/fpubh.2017.00053

[21] Bonham J. NBS Is Pandora's Box; New Techniques Make Anything Possible [Abstract]. Int J Neonatal Screen 2018; 4: 2. doi:10.3390/ijns4030028

[22] WHO. Vorsorgeuntersuchung und Screening: ein kurzer Leitfaden. Wirksamkeit erhöhen, Nutzen maximieren und Schaden minimieren [**2020**]. Im Internet: https://apps.who.int/iris/bitstream/handle/10665/330853/9789289054805-ger.pdf; Stand: 12.11.2021

[23] Gemeinsamer Bundesausschuss (G-BA). Richtlinie des Gemeinsamen Bundesausschusses über die Früherkennung von Krankheiten bei Kindern (Kinder-Richtlinie). zuletzt geändert am 16. September 2021, veröffentlicht im Bundesanzeiger AT 03.11.2021 B4, in Kraft getreten am 1. Januar 2022. Im Internet: https://www.g-ba.de/richtlinien/15/; Stand: 03.02.2022

[24] Bundesgesundheitsministerium. Gesetz über genetische Untersuchungen bei Menschen (Gendiagnostikgesetz, GenDG) (31.07.2009). Im Internet: https://www.bundesgesundheitsministerium.de/service/begriffe-von-a-z/g/gendiagnostikgesetz.html; Stand: 09.02.2022

[25] Deutsche Gesellschaft für Neugeborenenscreening e.V. (DGNS). DGNS Screeningreports. Im Internet: https://www.screening-dgns.de/reports.php; Stand: 13.04.2022

[26] Lüders A, Blankenstein O, Brockow I, et al. Neugeborenen-Screening auf angeborene Stoffwechsel- und Hormonstörungen. Ergebnisse der Jahre 2006–2018 aus Deutschland. Dtsch Arztebl Int 2021; 118: 101–108. doi:10.3238/arztebl.m2021.0009

[27] Committee for the Study of the Future of Public Health. Future of Public Health. Washington: National Academies Press; 1988

[28] Ruckstuhl B, Somaini B, Twisselmann W. Förderung der Qualität in Gesundheitsprojekten. Der Public Health Action Cycle als Arbeitsinstrument [Zürich **2008**] (2008). Im Internet: http://www.quint-essenz.ch/de/files/Foerderung_der_Qualitaet.pdf; Stand: 03.12.2021

[29] Mayring P. Qualitative Inhaltsanalyse. Grundlagen und Techniken. Weinheim: Belz; 2007

[30] Cornel MC, Rigter T, Weinreich SS, et al. A framework to start the debate on neonatal screening policies in the EU: an Expert Opinion Document. EJHG 2013; 22: 12–17. doi:10.1038/ejhg.2013.90

[31] Dobrow MJ, Hagens V, Chafe R, et al. Consolidated principles for screening based on a systematic review and consensus process. Canadian Medical Association journal 2018; 190: E422-E429. doi:10.1503/cmaj.171154

[32] Cornel MC, Rigter T, Jansen ME, et al. Neonatal and carrier screening for rare diseases: how innovation challenges screening criteria worldwide. J Community Genet 2021; 12: 257–265. doi:10.1007/s12687-020-00488-y

[33] Wiley V, Webster D, Loeber G. Screening Pathways through China, the Asia Pacific Region, the World. Int J Neonatal Screen 2019; 5: 26. doi:10.3390/ijns5030026

[34] Howson CP, Cedergren B, Giugliani R, et al. Universal newborn screening: A roadmap for action. Mol Genet Metab 2018; 124: 177–183. doi:10.1016/j.ymgme.2018.04.009

[35] Therrell BL, Padilla CD, Loeber JG, et al. Current status of newborn screening worldwide: 2015. Semin Perinatol 2015; 39: 171–187. doi:10.1053/j.semperi.2015.03.002

[36] Gemeinsamer Bundesausschuss (G-BA). Verfahrensordnung des Gemeinsamen Bundesausschusses. in der Fassung vom 18. Dezember 2008, veröffentlicht im Bundesanzeiger Nr. 84a (Beilage) vom 10. Juni 2009, in Kraft getreten am 1. April 2009, zuletzt geändert durch den Beschluss vom 18. März 2022, veröffentlicht im Bundesanzeiger BAnz AT 24.06.2022 B2, in Kraft getreten am 25. Juni 2022 (18.03.2022). Im Internet: https://www.g-ba.de/richtlinien/42/; Stand: 28.06.2022

[37] Robert Koch-Institut (RKI). Richtlinie der Gendiagnostik-Kommission (GEKO) für die Anforderungen an die Durchführung genetischer Reihenuntersuchungen gemäß § 23 Abs. 2 Nr. 6 GenDG : Revidierte Fassung vom 26.06.2020, veröffentlicht und in Kraft getreten am 16.07.2020, ersetzt die Fassung vom 16.11.2012. Bundesgesundheitsblatt Gesundheitsforschung Gesundheitsschutz 2020; 63: 1311–1317. doi:10.1007/s00103-020-03204-w

[38] Health Resources & Services Administration (HRSA). Recommended Uniform Screening Panel (RUSP). Im Internet: https://newbornscreening.hrsa.gov/about-newborn-screening/recommended-uniform-screening-panel; Stand: 25.01.2022

[39] RIVM (Staatsinstitut für öffentliche Gesundheit und Umwelt, Niederlande). Fact Sheet on Neonatal Heel Prick Screening 2021. Im Internet: https://www.pns.nl/sites/default/files/2022-04/Newborn%20blood%20spot%20screening%20fact%20sheet%202021%20-%20UK.pdf; Stand: 15.04.2022

[40] Oslo universitetssykehus Nyfødtscreeningen (Universitätsklinik Oslo Neugeborenenscreening). Nyfødtscreening - blodprøve av nyfødte (Neugeborenenscreening - Bluttest bei Neugeborenen). Im Internet: https://oslo-universitetssykehus.no/behandlinger/nyfodtscreening-blodprove-av-nyfodte; Stand: 10.08.2021

[41] Röschinger W, Sonnenschein S, Schuhmann E, et al. Neue Zielerkrankungen im Neugeborenenscreening. Monatsschr Kinderheilkd 2015; 163: 142–149. doi:10.1007/s00112-014-3297-0

[42] Gramer G, Hoffmann GF. Second-tier strategies in newborn screening – Potential and limitations. medgen 2022; 34: 21–28. doi:10.1515/medgen-2022-2117

[43] Wilcken B. Newborn Screening for Lysosomal Disease: Mission Creep and a Taste of Things to Come? Int J Neonatal Screen 2018; 4: 21. doi:10.3390/ijns4030021

[44] Sanders KA, Gavrilov DK, Oglesbee D, et al. A Comparative Effectiveness Study of Newborn Screening Methods for Four Lysosomal Storage Disorders. Int J Neonatal Screen 2020; 6. doi:10.3390/ijns6020044

[45] Moser AB, Seeger E, Raymond GV. Newborn Screening for X-Linked Adrenoleukodystrophy: Past, Present, and Future. Int J Neonatal Screen 2022; 8. doi:10.3390/ijns8010016

[46] Schaaf CP, Kölker S, Hoffmann GF. Genomic newborn screening: Proposal of a two-stage approach. J Inherit Metab Dis 2021; 44: 518–520. doi:10.1002/jimd.12381

[47] Berg JS, Powell CM. Potential Uses and Inherent Challenges of Using Genome-Scale Sequencing to Augment Current Newborn Screening. Cold Spring Harb Perspect Med 2015; 5. doi:10.1101/cshperspect.a023150

[48] Remec ZI, Trebusak Podkrajsek K, Repic Lampret B, et al. Next-Generation Sequencing in Newborn Screening: A Review of Current State. Front Genet 2021; 12: 662254. doi:10.3389/fgene.2021.662254

[49] Downie L, Halliday J, Lewis S, et al. Principles of Genomic Newborn Screening Programs: A Systematic Review. JAMA Netw Open 2021; 4: e2114336. doi:10.1001/jamanetworkopen.2021.14336

[50] Morava E, Baumgartner M, Patterson M, et al. Newborn screening: To WES or not to WES, that is the question. J Inherit Metab Dis 2020; 43: 904–905. doi:10.1002/jimd.12303

[51] Adhikari AN, Gallagher RC, Wang Y, et al. The role of exome sequencing in newborn screening for inborn errors of metabolism. Nat Med 2020; 26: 1392–1397. doi:10.1038/s41591-020-0966-5

[52] Tangeraas T, Sæves I, Klingenberg C, et al. Performance of Expanded Newborn Screening in Norway Supported by Post-Analytical Bioinformatics Tools and Rapid Second-Tier DNA Analyses. Int J Neonatal Screen 2020; 6. doi:10.3390/ijns6030051

[53] Wojcik MH, Zhang T, Ceyhan-Birsoy O, et al. Discordant results between conventional newborn screening and genomic sequencing in the BabySeq Project. Genet Med 2021; 23: 1372–1375. doi:10.1038/s41436-021-01146-5

[54] Lund AM, Wibrand F, Skogstrand K, et al. Use of Molecular Genetic Analyses in Danish Routine Newborn Screening. Int J Neonatal Screen 2021; 7: 50. doi:10.3390/ijns7030050

[55] Franková V, Driscoll RO, Jansen ME, et al. Regulatory landscape of providing information on newborn screening to parents across Europe. EJHG 2021; 29: 67–78. doi:10.1038/s41431-020-00716-6

[56] Dhondt J-L. Neonatal screening: from the 'Guthrie age' to the 'genetic age'. J Inherit Metab Dis 2007; 30: 418–422. doi:10.1007/s10545-007-0624-9

[57] Ulph F, Wright S, Dharni N, et al. Provision of information about newborn screening antenatally: a sequential exploratory mixed-methods project. Health Technol Assess 2017; 21: 1–240. doi:10.3310/hta21550

[58] Potter BK, Etchegary H, Nicholls SG, et al. Education and parental involvement in decision-making about newborn screening: understanding goals to clarify content. J Genet Couns 2015; 24: 400–408. doi:10.1007/s10897-014-9780-x

[59] Howell RR. Ethical Issues Surrounding Newborn Screening. Int J Neonatal Screen 2021; 7. doi:10.3390/ijns7010003

[60] Orzalesi M, Danhaive O. Ethical problems with neonatal screening. Ann Ist Super Sanita 2009; 45: 325–330

[61] Moyer VA, Calonge N, Teutsch SM, et al. Expanding newborn screening: process, policy, and priorities. Hastings Cent Rep 2008; 38: 32–39. doi:10.1353/hcr.0.0011

[62] Cragun D, DeBate RD, Pal T. Applying public health screening criteria: how does universal newborn screening compare to universal tumor screening for Lynch syndrome in adults with colorectal cancer? J Genet Couns 2015; 24: 409–420. doi:10.1007/s10897-014-9769-5

[63] Bonham JR. Impact of new screening technologies: should we screen and does phenotype influence this decision? J Inherit Metab Dis 2013; 36: 681–686. doi:10.1007/s10545-013-9598-y

[64] Rothwell E, Johnson E, Wong B, et al. Comparison of Video, App, and Standard Consent Processes on Decision-Making for Biospecimen Research: A Randomized Controlled Trial. J Empir Res Hum Res Ethics 2020; 15: 252–260. doi:10.1177/1556264620913455

[65] Loeber JG, Burgard P, Cornel MC, et al. Newborn screening programmes in Europe; arguments and efforts regarding harmonization. Part 1. From blood spot to screening result. J Inherit Metab Dis 2012; 35: 603–611. doi:10.1007/s10545-012-9483-0

[66] Clinical and Laboratory Standards Institute (CLSI). NBS02-A2: Newborn Screening Follow-up; Approved Guideline—Second Edition; 2013

[67] Vass CM, Georgsson S, Ulph F, et al. Preferences for aspects of antenatal and newborn screening: a systematic review. BMC Pregnancy Childbirth 2019; 19: 131. doi:10.1186/s12884-019-2278-7

[68] Anderson R, Rothwell E, Botkin JR. Newborn screening: ethical, legal, and social implications. Annu Rev Nurs Res 2011; 29: 113–132. doi:10.1891/0739-6686.29.113

[69] White AL, Boardman F, McNiven A, et al. Absorbing it all: A meta-ethnography of parents' unfolding experiences of newborn screening. Soc Sci Med 2021; 287: 114367. doi:10.1016/j.socscimed.2021.114367

[70] IJzebrink A, van Dijk T, Franková V, et al. Informing Parents about Newborn Screening: A European Comparison Study. Int J Neonatal Screen 2021; 7. doi:10.3390/ijns7010013

[71] Cocanour CS. Informed consent-It's more than a signature on a piece of paper. Am J Surg 2017; 214: 993–997. doi:10.1016/j.amjsurg.2017.09.015

[72] Rosas-Blum E, Shirsat P, Leiner M. Communicating genetic information: a difficult challenge for future pediatricians. BMC Med Educ 2007; 7: 17. doi:10.1186/1472-6920-7-17

[73] MacDougall DR. Must Consent Be Informed? Patient rights, state authority, and the moral basis of the physician's duties of disclosure 2021; 31: 247–270

[74] Kegley JAK. An ethical imperative: genetics education for physicians and patients. Medicine and Law 2003; 22: 275–283

[75] National Screening Unit New Zealand. Guidelines for practitioners providing services within the Newborn Metabolic Screening Programme in New Zealand (2010). Im Internet: https://www.nsu.govt.nz/health-professionals/newborn-metabolic-screening-programme/procedures-guidelines-and-reports; Stand: 14.11.2021

[76] RIVM (Staatsinstitut für öffentliche Gesundheit und Umwelt, Niederlande). Checklist voorlichtingsgesprek verloskundig zorgverleners (Checkliste Informationsgespräch für Anbieter von Geburtshilfeleistungen) (28.11.2021). Im Internet: https://draaiboekhielprikscreening.rivm.nl/documenten/checklist-voorlichtingsgesprek-verloskundig-zorgverleners; Stand: 01.12.2021

[77] Oslo universitetssykehus Nyfødtscreeningen (Universitätsklinik Oslo Neugeborenenscreening). RETNINGSLINJER FOR HÅNDTERING AV PRØVER TIL NYFØDTSCREENING. RICHTLINIEN FÜR DEN UMGANG MIT PROBEN FÜR DAS NEUGEBORENENSCREENING. Im Internet: https://oslo-universitetssykehus.no/PublishingImages/avdelinger/barne-og-ungdomsklinikken/nyfodtscreeningen/nyfodtscreening/Retningslinjer%20for%20h%C3%A5ndtering%20av%20pr%C3%B8ver%20til%20Nyf%C3%B8dtscreeningen.pdf; Stand: 16.11.2021

[78] Public Health England. Collection Newborn blood spot screening programme: supporting publications. Guidance and resources for health professionals and commissioners working in the NHS newborn blood spot (NBS) screening programme. (03.09.2021). Im Internet: https://www.gov.uk/government/collections/newborn-blood-spot-screening-programme-supporting-publications#programme-pathway; Stand: 03.12.2021

[79] Newborn Screening Ontario. Im Internet: https://www.newbornscreening.on.ca/en; Stand: 07.12.2021

[80] RIVM (Staatsinstitut für öffentliche Gesundheit und Umwelt, Niederlande). Hielprik en gehoortest bij pasgeborenen (Fersenblutentnahme und Hörtest bei Neugeborenen) (26.07.2021). Im Internet: https://www.pns.nl/documenten/hielprik-en-gehoortest-bij-pasgeborenen; Stand: 16.01.2022

[81] Public Health England. Screening tests for you and your baby - Documents (03.05.2019). Im Internet: https://www.gov.uk/government/publications/screening-tests-for-you-and-your-baby; Stand: 16.01.2022

[82] Victorian Clinical Genetics Services VCGS. Newborn Bloodspot Screening. Im Internet: https://www.vcgs.org.au/tests/newborn-bloodspot-screening; Stand: 24.08.2021

[83] Saske (Screeninglabor Finnland). Esitteet perheille (Broschüren für Familien) (16.06.2021). Im Internet: https://www.vsshp.fi/fi/saske/esitteet/Sivut/default.aspx; Stand: 16.01.2022

[84] Provincial Health Services Authority British Columbia. Newborn Screening. Im Internet: http://www.perinatalservicesbc.ca/our-services/screening-programs/newborn-screening-program; Stand: 25.01.2022

[85] Newborn Screening Ontario. Screening Resources. Im Internet: https://www.newbornscreening.on.ca/en/%C3%A0-propos-du-d%C3%A9pistage/screening-resources; Stand: 17.01.2022

[86] Karolinska Universitetslaboratoriet (Universitätslabor Karolinska, Stockholm). Föräldrainformation PKU-provet för på-sidan-översättning (Elterninformation PKU-Test für On-Page-Übersetzung) (13.04.2022). Im Internet: https://www.karolinska.se/for-vardgivare/karolinska-universitetslaboratoriet/centrum-for-medfodda-metabola-sjukdomar/foraldrainformation-pku-provet/; Stand: 17.05.2022

[87] Public Health England. Blood spot tests: easy guider. Im Internet: https://www.gov.uk/government/publications/screening-tests-for-you-and-your-baby-easy-guides; Stand: 14.08.2021

[88] RIVM (Staatsinstitut für öffentliche Gesundheit und Umwelt, Niederlande). Heel prick for Amanda and Jack's baby. Im Internet: https://www.pns.nl/hielprik/beeldverhaal/heel-prick-for-amanda-and-jacks-baby; Stand: 24.10.2021

[89] Victoria State Government, Department of Health. Newborn bloodspot screening - Better Health Channel. Im Internet: https://www.betterhealth.vic.gov.au/health/conditionsandtreatments/newborn-screening; Stand: 24.08.2021

[90] Sydney Children's Hospitals Network. NSW Newborn Screening Program (24.04.2020). Im Internet: https://www.schn.health.nsw.gov.au/find-a-service/laboratory-services/newborn-screening; Stand: 24.08.2021

[91] Agentschap zorg & gezondheid Vlaanderen (Amt für Pflege und Gesundheit Flandern, Belgien). Bevolkings Onderzoek aangeboren aandoeningen - Filmpjes (Bevölkerungs-Untersuchung angeborener Erkrankungen - Filme) (22.03.2022). Im Internet: https://aangeboren.bevolkingsonderzoek.be/nl/aa/filmpjes; Stand: 17.04.2022

[92] Programa Nacional de Tamizaje Neonatal Costa Rica (Nationales Neugeborenen-Screening-Programm Costa Rica) (14.12.2021). Im Internet: http://www.tamizajecr.com/; Stand: 08.04.2022

[93] National Screening Unit New Zealand. Newborn Metabolic Screening Programme - heel prick test (02.12.2021). Im Internet: https://www.nsu.govt.nz/pregnancy-newborn-screening/newborn-metabolic-screening-programme-heel-prick-test; Stand: 02.12.2021

[94] RIVM (Staatsinstitut für öffentliche Gesundheit und Umwelt, Niederlande). Video: de hielprik bij ouders thuis (Video: der Fersenstich im Elternhaus). Im Internet: https://www.pns.nl/hielprik/video-hielprik-bij-ouders-thuis/video-hielprik-bij-ouders-thuis; Stand: 01.04.2022

[95] Oslo universitetssykehus Nyfødtscreeningen (Universitätsklinik Oslo Neugeborenenscreening). Nyfødtscreening (Neugeborenenscreening). Im Internet: https://oslo-universitetssykehus.no/avdelinger/barne-og-ungdomsklinikken/nyfodtscreeningen/nyfodtscreening; Stand: 23.08.2021

[96] Karolinska Universitetslaboratoriet (Universitätslabor Karolinska, Stockholm). PKU test. Film om PKU-prov på engelska (Film zum PLU-Test auf englisch). Im Internet: https://karolinska.mediaflowportal.com/extern/watch/698035/; Stand: 17.01.2022

[97] Newborn Screening Ontario. NSO YouTubeChannel. Im Internet: https://www.youtube.com/user/NBSOntario; Stand: 17.01.2022

[98] Public Health England. Guidance Blood spot. Im Internet: https://www.gov.uk/government/publications/screening-tests-for-you-and-your-baby/blood-spot; Stand: 17.01.2022

[99] www.BabysFirstTest.org. Baby's First Test: Newborn Screening Playlist. Im Internet: https://www.youtube.com/user/BabysFirstTest; Stand: 16.01.2022

[100] Lüders A. „U0“ – Pilotprojekt zur Elternberatung vor der Geburt in der Kinder- und Jugendarztpraxis. Bayerisches Ärzteblatt; 2021: 330

[101] Vittozzi L, Hoffmann GF, Cornel M, Loeber G. Evaluation of population newborn screening practices for rare disorders in member states of the European Union (2011). Im Internet: https://www.isns-neoscreening.org/guidelines/; Stand: 15.12.2021

[102] Chen B, Mei J, Kalman L, et al. Good laboratory practices for biochemical genetic testing and newborn screening for inherited metabolic disorders. Morbidity and Mortality Weekly Report 2012; 61: 1–37

[103] Jesús VR de, Mei JV, Cordovado SK, et al. The Newborn Screening Quality Assurance Program at the Centers for Disease Control and Prevention: Thirty-five Year Experience Assuring Newborn Screening Laboratory Quality. Int J Neonatal Screen 2015; 1: 13–26. doi:10.3390/ijns1010013

[104] Bonham JR. The organisation of training for laboratory scientists in inherited metabolic disease, newborn screening and paediatric clinical chemistry. Clin Biochem 2014; 47: 763–764. doi:10.1016/j.clinbiochem.2014.05.050

[105] Public Health England. NHS Newborn Blood Spot Screening Programme. A laboratory guide to newborn blood spot screening for inherited metabolic diseases. Im Internet: https://assets.publishing.service.gov.uk/government/uploads/system/uploads/attachment_data/file/642333/IMD_laboratory_handbook_2017.pdf; Stand: 28.02.2022

[106] Public Health England. Laboratory guide to screening for CHT in the UK - GOV.UK. Im Internet: https://www.gov.uk/government/publications/congenital-hypothyroidism-screening-laboratory-handbook; Stand: 28.02.2022

[107] Webster D. Quality performance of newborn screening systems: strategies for improvement. J Inherit Metab Dis 2007; 30: 576–584. doi:10.1007/s10545-007-0639-2

[108] Bhattacharya K, Wotton T, Wiley V. The evolution of blood-spot newborn screening. Transl Pediatr 2014; 3: 63–70. doi:10.3978/j.issn.2224-4336.2014.03.08

[109] Vittozzi L, Burgard P, Cornel M, Hoffmann GF, Lindner M, Loeber JG, Rigter T, Rupp K, Taruscio D, Weinreich S. Executive Report to the European Commission on newborn screening in the European Union (02.07.2011). Im Internet: https://www.isns-neoscreening.org/guidelines/; Stand: 15.12.2021

[110] Marquardt G, Currier R, McHugh DMS, et al. Enhanced interpretation of newborn screening results without analyte cutoff values. Genet Med 2012; 14: 648–655. doi:10.1038/gim.2012.2

[111] Fleischman A, Thompson JD, Glass M. Systematic Data Collection to Inform Policy Decisions: Integration of the Region 4 Stork (R4S) Collaborative Newborn Screening Database to Improve MS/MS Newborn Screening in Washington State. JIMD Rep 2014; 13: 15–21. doi:10.1007/8904_2013_266

[112] Hall PL, Marquardt G, McHugh DMS, et al. Postanalytical tools improve performance of newborn screening by tandem mass spectrometry. Genet Med 2014; 16: 889–895. doi:10.1038/gim.2014.62

[113] Maase R, Bouva M, Rinaldo P, et al. Striving towards a Better Predictive Value from the Dutch Newborn Screening Results [Abstract]. Int J Neonatal Screen 2018; 4: 31. doi:10.3390/ijns4030028

[114] Jones D, Garcia S, Ruiz-Schultz N, Gaviglio A, Cuthbert C. A Strong Start: Enhancing Newborn Screening for Precision Public Health | Blogs | CDC (15.11.2021). Im Internet: https://blogs.cdc.gov/genomics/2020/10/13/a-strong-start/; Stand: 15.11.2021

[115] Gavrilov DK, Piazza AL, Pino G, et al. The Combined Impact of CLIR Post-Analytical Tools and Second Tier Testing on the Performance of Newborn Screening for Disorders of Propionate,

Methionine, and Cobalamin Metabolism. Int J Neonatal Screen 2020; 6: 33. doi:10.3390/ijns6020033

[116] Sörensen L, Döbeln U von, Åhlman H, et al. Expanded Screening of One Million Swedish Babies with R4S and CLIR for Post-Analytical Evaluation of Data. Int J Neonatal Screen 2020; 6: 42. doi:10.3390/ijns6020042

[117] Rowe AD, Stoway SD, Åhlman H, et al. A Novel Approach to Improve Newborn Screening for Congenital Hypothyroidism by Integrating Covariate-Adjusted Results of Different Tests into CLIR Customized Interpretive Tools. Int J Neonatal Screen 2021; 7. doi:10.3390/ijns7020023

[118] Caggana M, Jones EA, Shahied SI, et al. Newborn screening: from Guthrie to whole genome sequencing. Public Health Rep 2013; 128 Suppl 2: 14–19. doi:10.1177/00333549131280S204

[119] Peng G, Tang Y, Cowan TM, et al. Reducing False-Positive Results in Newborn Screening Using Machine Learning. Int J Neonatal Screen 2020; 6. doi:10.3390/ijns6010016

[120] Mayoclinics. CLIR - About CLIR (22.09.2021). Im Internet: https://clir.mayo.edu/Home/About; Stand: 22.09.2021

[121] Rinaldo P. Precision Newborn Screening Driven by Results Adjustments for Multiple Covariates [Abstract]. Int J Neonatal Screen 2018; 4: 5. doi:10.3390/ijns4030028

[122] Janzen N, Peter M, Sander S, et al. Newborn screening for congenital adrenal hyperplasia: additional steroid profile using liquid chromatography-tandem mass spectrometry. J Clin Endocrinol Metab 2007; 92: 2581–2589. doi:10.1210/jc.2006-2890

[123] Gesellschaft für Neonatologie und Pädiatrische Intensivmedizin (GNPI). AWMF-S2k-Leitlinie 024/012 "Neugeborenen-Screening auf angeborene Stoffwechselstörungen, Endokrinopathien, schwere kombinierte Immundefekte (SCID), Sichelzellkrankheit, 5q-assoziierte spinale Muskelatrophie (SMA) und Mukoviszidose" (10.02.2022). Im Internet: https://www.awmf.org/leitlinien/detail/ll/024-012.html; Stand: 07.03.2022

[124] Centers for Disease Control and Prevention. Newborn Screening Quality Assurance Program (NSQAP). About the Program. Im Internet: https://www.cdc.gov/labstandards/nsqap_about.html; Stand: 07.02.2022

[125] Pickens CA, Sternberg M, Seeterlin M, et al. Harmonizing Newborn Screening Laboratory Proficiency Test Results Using the CDC NSQAP Reference Materials. Int J Neonatal Screen 2020; 6: 75. doi:10.3390/ijns6030075

[126] Hall PL, Wittenauer A, Hagar A. Post-Analytical Tools for the Triage of Newborn Screening Results in Follow-up Can Reduce Confirmatory Testing and Guide Performance Improvement. Int J Neonatal Screen 2020; 6: 20. doi:10.3390/ijns6010020

[127] Loeber JG, Platis D, Zetterström RH, et al. Neonatal Screening in Europe Revisited: An ISNS Perspective on the Current State and Developments Since 2010. Int J Neonatal Screen 2021; 7: 15. doi:10.3390/ijns7010015

[128] United Kingdom National External Quality Assessment Service (UK NEQAS). Newborn Screening (16.05.2017). Im Internet: https://ukneqas.org.uk/programmes/result/?programme=newborn-screening; Stand: 09.02.2022

[129] RIVM (Staatsinstitut für öffentliche Gesundheit und Umwelt, Niederlande). Referentielaboratorium (Referenzlabor) (06.05.2022). Im Internet: https://draaiboekhielprikscreening.rivm.nl/rol-ketenpartners/referentielaboratorium; Stand: 09.12.2021

[130] RIVM (Staatsinstitut für öffentliche Gesundheit und Umwelt, Niederlande). Afkapgrenzen en beslissingscriteria neonatale screening (Grenzwerte und Entscheidungskriterien für das Neugeborenenscreening) (01.01.2022). Im Internet: https://webshare.iprova.nl/lv3tvdk3gr61tbnx/Document.aspx; Stand: 26.01.2022

[131] Strand J. Second Tier Genetic Testing in the Norwegian National Screening Program [Abstract]. Int J Neonatal Screen 2021; 7: 2–3. doi:10.3390/ijns7040071

[132] Moody L, Atkinson L, Kehal I, et al. Healthcare professionals' and parents' experiences of the confirmatory testing period: a qualitative study of the UK expanded newborn screening pilot. BMC Pediatr 2017; 17: 121. doi:10.1186/s12887-017-0873-1

[133] Schmidt JL, Castellanos-Brown K, Childress S, et al. The impact of false-positive newborn screening results on families: a qualitative study. Genet Med 2012; 14: 76–80. doi:10.1038/gim.2011.5

[134] Finan C, Nasr SZ, Rothwell E, et al. Primary care providers' experiences notifying parents of cystic fibrosis newborn screening results. Clin Pediatr (Phila) 2015; 54: 67–75. doi:10.1177/0009922814545619

[135] Salm N, Yetter E, Tluczek A. Informing parents about positive newborn screen results: parents' recommendations. J Child Health Care 2012; 16: 367–381. doi:10.1177/1367493512443906

[136] Tangeraas T. What Happens after Recall, Who Should Give the Results to Families? [Abstract]. Int J Neonatal Screen 2021; 7: 10. doi:10.3390/ijns7040071

[137] Viall S, Jain S, Chapman K, et al. Short-term follow-up systems for positive newborn screens in the Washington Metropolitan Area and the United States. Mol Genet Metab 2015; 116: 226–230. doi:10.1016/j.ymgme.2015.11.002

[138] Chudleigh J, Holder P, Moody L, et al. Process evaluation of co-designed interventions to improve communication of positive newborn bloodspot screening results. BMJ Open 2021; 11: e050773. doi:10.1136/bmjopen-2021-050773

[139] Barben J, Chudleigh J. Processing Newborn Bloodspot Screening Results for CF. Int J Neonatal Screen 2020; 6: 25. doi:10.3390/ijns6020025

[140] Brockow I, Nennstiel U. Parents' experience with positive newborn screening results for cystic fibrosis. Eur J Pediatr 2019; 178: 803–809. doi:10.1007/s00431-019-03343-6

[141] Chudleigh J, Ren CL, Barben J, et al. International approaches for delivery of positive newborn bloodspot screening results for CF. J Cyst Fibros 2019; 18: 614–621. doi:10.1016/j.jcf.2019.04.004

[142] Rosenau H, Steffen F. Legal aspects of Newborn Screening. medgen 2022; 34: 3-11. doi:10.1515/medgen-2022-2110

[143] Statens Serum Institut (Staatliches Serum-Institut Dänemark). Screening af nyfødte (Neugeborenen-Screening). Im Internet: https://nyfoedte.ssi.dk/; Stand: 24.08.2021

[144] Rocha H, Marcão A, Sousa C, et al. PROGRAMA PORTUGUÉS DE CRIBADO NEONATAL / Portuguese Newborn Screening Program. Rev Esp Salud Publica 2021; 95: e1-7

[145] Zetterström R, Sörensen L. IT Infrastructure for Long-Term Follow-Up for Newborn Screening in Sweden [Abstract]. Int J Neonatal Screen 2021; 7: 7. doi:10.3390/ijns7040071

[146] Jacob H. Update on expanded newborn screening. Arch Dis Child Educ Pract Ed 2016; 101: 139. doi:10.1136/archdischild-2015-309429

[147] Rueegg CS, Barben J, Hafen GM, et al. Newborn screening for cystic fibrosis - The parent perspective. J Cyst Fibros 2016; 15: 443–451. doi:10.1016/j.jcf.2015.12.003

[148] National Screening Unit New Zealand. Newborn Metabolic Screening Resources (02.12.2021). Im Internet: https://www.nsu.govt.nz/health-professionals/newborn-metabolic-screening-programme/newborn-metabolic-screening-resources; Stand: 02.12.2021

[149] Public Health England. Newborn screening: information leaflets. These publications explain NHS newborn screening programme tests, possible results and follow-up tests. (29.07.2021). Im Internet: https://www.gov.uk/government/collections/newborn-screening-information-leaflets; Stand: 14.08.2021

[150] Newborn Screening Ontario. Disease Information (10.12.2021). Im Internet: https://www.newbornscreening.on.ca/en/disease; Stand: 10.12.2021

[151] Newborn Screening Ontario. A healthy start leads to a healthier life. Early detection saves lives. Im Internet: https://www.newbornscreening.on.ca/en; Stand: 22.11.2021

[152] Kurkijärvi R. The Status of Newborn Screening in Finland—Country Report [Abstract]. Int J Neonatal Screen 2021; 7: 49–50. doi:10.3390/ijns7040071

[153] Ministry of Health Israel. Get your baby's genetic screening results. Im Internet: https://www.gov.il/en/service/newborn-national-genetic-testing-results; Stand: 11.11.2021

[154] Instituto Nacional de Saúde (Nationales Institut für Gesundheit Portugal). Resultados do teste do pezinho (Ergebnisse des Fersenstich-Tests). Im Internet: https://resultados-dp-insa.min-saude.pt/; Stand: 19.08.2021

[155] RIVM (Staatsinstitut für öffentliche Gesundheit und Umwelt, Niederlande). Factsheet Newborn Blood spot screening 2020. Im Internet: https://www.pns.nl/sites/default/files/2021-02/Newborn%20blood%20spot%20screening%20fact%20sheet%20dec2020_UK_0.pdf; Stand: 02.12.2021

[156] National Health Service NHS. Newborn blood spot test (19.03.2021). Im Internet: https://www.nhs.uk/conditions/baby/newborn-screening/blood-spot-test/; Stand: 03.08.2021

[157] Robert Koch-Institut (RKI). Richtlinie der Gendiagnostik-Kommission (GEKO) für die Anforderungen an die Inhalte der Aufklärung bei genetischen Untersuchungen zu medizinischen Zwecken gemäß 23 Abs. 2 Nr. 3 GenDG. revidierte Fassung vom 24.06.2022, veröffentlicht und in Kraft getreten am 01.07.2022, ersetzt die Fassung vom 28.04.2017. Im Internet: https://www.rki.de/DE/Content/Kommissionen/GendiagnostikKommission/Richtlinien/RL_Aufklaerung_med_Zwecke.html; Stand: 28.07.2022

[158] Wilcken B. Screening for disease in the newborn: the evidence base for blood-spot screening. Pathology 2012; 44: 73–79. doi:10.1097/PAT.0b013e32834e843f

[159] Deutsche Gesellschaft für Kinder- und Jugendmedizin (DGKJ). AWMF-S1-Leitlinie 027-021 "Konfirmationsdiagnostik bei Verdacht auf angeborene Stoffwechselkrankheiten aus dem Neugeborenenscreening". Im Internet: https://www.awmf.org/leitlinien/detail/ll/027-021.html; Stand: 20.04.2022

[160] Deutscher Ethikrat. Herausforderungen im Umgang mit seltenen Erkrankungen. AD-HOC-EMPFEHLUNG [Berlin 2018]. Im Internet: https://www.ethikrat.org/publikationen/publikationsdetail/?tx_wwt3shop_detail%5Bproduct%5D=116&tx_wwt3=&cHash=b3e78fc99b523a5226a74aca8f971b95; Stand: 13.12.2021

[161] Nationales Aktionsbündnis für Menschen mit Seltenen Erkrankungen (NAMSE). Nationaler Aktionsplan für Menschen mit Seltenen Erkrankungen. Handlungsfelder, Empfehlungen und Maßnahmenvorschläge [2013]. Im Internet: https://www.namse.de/; Stand: 03.02.2022

[162] Sontag MK, Sarkar D, Comeau AM, et al. Case Definitions for Conditions Identified by Newborn Screening Public Health Surveillance. Int J Neonatal Screen 2018; 4: 16. doi:10.3390/ijns4020016

[163] Heather N, Webster D. It All Depends What You Count-The Importance of Definitions in Evaluation of CF Screening Performance. Int J Neonatal Screen 2020; 6: 47. doi:10.3390/ijns6020047

[164] Blom M, Zetterström RH, Stray-Pedersen A, et al. Recommendations for uniform definitions used in newborn screening for severe combined immunodeficiency. Journal of Allergy and Clinical Immunology 2021; 149: 1428–1436. doi:10.1016/j.jaci.2021.08.026

[165] Kronn D, Mofidi S, Braverman N, et al. Diagnostic guidelines for newborns who screen positive in newborn screening. Genet Med 2010; 12: S251-5. doi:10.1097/GIM.0b013e3181fe5d8b

[166] Gesellschaft für Pädiatrische Pneumologie (GPP). AWMF-S2-Konsensus-Leitlinie 026-023 "Diagnose der Mukoviszidose". Im Internet: https://www.awmf.org/uploads/tx_szleitlinien/026-023lS_2k_Diagnose_der_Mukoviszidose_2013-07-abgelaufen.pdf; Stand: 10.04.2022

[167] Deutsche Gesellschaft für Kinderendokrinologie und -diabetologie (DGKED). AWMF-S1-Leitlinie 174-003 "Adrenogenitales Syndrom (AGS) im Kindes- und Jugendalter"; Stand: 10.04.2022

[168] Gesellschaft für Pädiatrische Onkologie und Hämatologie (GPOH). AWMF-S2k-Leitlinie 025/016 „Sichelzellkrankheit“. Im Internet: https://www.awmf.org/leitlinien/detail/ll/025-016.html; Stand: 10.04.2022

[169] Deutsche Gesellschaft für Immunologie (DGfI), Arbeitsgemeinschaft Pädiatrische Immunologie (API). AWMF-S2k-Leitlinie „Diagnostik auf Vorliegen eines primären Immundefekts“. Abklärung von Infektionsanfälligkeit, Immundysregulation und weiteren Symptomen von primären Immundefekten. Im Internet: https://www.awmf.org/leitlinien/detail/ll/112-001.html; Stand: 10.04.2022

[170] Gesellschaft für Neuropädiatrie (GNP). AWMF-S1-Leitlinie 022-030 "Spinale Muskelatrophie (SMA), Diagnostik und Therapie". Im Internet: https://www.awmf.org/leitlinien/detail/ll/022-030.html; Stand: 10.04.2022

[171] RIVM (Staatsinstitut für öffentliche Gesundheit und Umwelt, Niederlande). Hielprik voor professionals (Fersenstich für Fachleute) (30.11.2021). Im Internet: https://www.pns.nl/professionals/hielprik; Stand: 30.11.2021

[172] American College of Medical Genetics and Genomics (ACMG). ACT Sheets and Algorithms. Im Internet: https://www.acmg.net/ACMG/Medical-Genetics-Practice-Resources/ACT_Sheets_and_Algorithms.aspx; Stand: 21.01.2022

[173] Association of Public Health Laboratories (APHL). Case Definitions for Newborn Screening. Im Internet: https://www.newsteps.org/case-definitions; Stand: 07.02.2022

[174] Feuchtbaum L, Dowray S, Lorey F. The context and approach for the California newborn screening short- and long-term follow-up data system: preliminary findings. Genet Med 2010; 12: S242-50. doi:10.1097/GIM.0b013e3181fe5d66

[175] Feuchtbaum L, Yang J, Currier R. Follow-up status during the first 5 years of life for metabolic disorders on the federal Recommended Uniform Screening Panel. Genet Med 2018; 20: 831–839. doi:10.1038/gim.2017.199

[176] RIVM (Staatsinstitut für öffentliche Gesundheit und Umwelt, Niederlande). Neonatale registratie afwijkende hielprikscreening Neorah (Neugeborenenregistrierung abnormales Fersenblut-Screening Neorah). Im Internet: https://bronnen.zorggegevens.nl/Bron?naam=Neonatale-registratie-afwijkende-hielprikscreening; Stand: 22.09.2021

[177] Bräuer P. Rechtsfragen des Neugeborenen-Screenings unter besonderer Beachtung des Datenschutzrechts. Tübingen: Eberhard Karls Universität Tübingen; 2020

[178] Darby E, Thompson J, Johnson C, et al. Establishing a National Community of Practice for Newborn Screening Follow-Up. Int J Neonatal Screen 2021; 7: 49. doi:10.3390/ijns7030049

[179] Brosco JP, Grosse SD, Ross LF. Universal state newborn screening programs can reduce health disparities. JAMA Pediatr 2015; 169: 7–8. doi:10.1001/jamapediatrics.2014.2465

[180] Zimmer K-P. Neugeborenen-Screening: noch Platz für Verbesserungen: Editorial. Dtsch Arztebl Int 2021; 118: 99–100. doi:10.3238/arztebl.m2021.0008

[181] Public Health England. Guidance Newborn blood spot screening: failsafe solution user guide. How to use the newborn blood spot screening failsafe solution (NBSFS), the national IT system, to minimise risk to babies. (17.09.2021). Im Internet: https://www.gov.uk/government/publications/newborn-blood-spot-screening-failsafe-solution-user-guide; Stand: 03.12.2021

[182] Office de la Naissance et de l'Enfance ONE (Amt für Geburt und Kindheit, Belgien). Depistage des maladies metaboliques et endocriniennes chez les nouveaux-nes - Professionnels. Documents scientifiques (29.12.2021). Im Internet: http://www.depistageneonatal.be/pro_anomalies_congenitales/index.htm; Stand: 29.12.2021

[183] Souza A de, Wolan V, Battochio A, et al. Newborn Screening: Current Status in Alberta, Canada. Int J Neonatal Screen 2019; 5: 37. doi:10.3390/ijns5040037

[184] Newborn Screening Ontario. Newborn Screening Manual (2018). Im Internet: https://www.newbornscreening.on.ca/sites/default/files/newborn_screening_manual_2018_-_web_0.pdf; Stand: 10.12.2021

[185] Liebl B, Nennstiel-Ratzel U, Kries R von, et al. Expanded newborn screening in Bavaria: tracking to achieve requested repeat testing. Prev Med 2002; 34: 132–137. doi:10.1006/pmed.2001.0954

[186] Bayerisches Landesamt für Gesundheit und Lebensmittelsicherheit. Gesundheit: Neugeborenen-Screening (29.12.2021). Im Internet: https://www.lgl.bayern.de/gesundheit/praevention/kindergesundheit/neugeborenenscreening/index.htm; Stand: 07.03.2022

[187] BabysFirstTest.org. What Your State Offers (09.05.2022). Im Internet: https://www.babysfirsttest.org/newborn-screening/states; Stand: 09.02.2022

[188] Percenti L, Vickery G. Newborn Screening Follow-up. N C Med J 2019; 80: 37–41. doi:10.18043/ncm.80.1.37

[189] Association of Public Health Laboratories (APHL). NewSTEPs Annual Report 2020 (2021). Im Internet: https://www.newsteps.org/sites/default/files/resources/download/NewSTEPs%20Annual%20Report%209%2022%2021.pdf; Stand: 07.12.2021

[190] RIVM (Staatsinstitut für öffentliche Gesundheit und Umwelt, Niederlande). Registratie in NEORAH (Registrierung in NEORAH) (30.11.2021). Im Internet: https://www.pns.nl/documenten/registratie-in-neorah; Stand: 30.11.2021

[191] Kemper AR, Boyle CA, Brosco JP, et al. Ensuring the Life-Span Benefits of Newborn Screening. Pediatrics 2019; 144. doi:10.1542/peds.2019-0904

[192] Hinman AR, Mann MY, Singh RH. Newborn dried bloodspot screening: mapping the clinical and public health components and activities. Genet Med 2009; 11: 418–424. doi:10.1097/GIM.0b013e31819f1b33

[193] Sheller R, Ojodu J, Griffin E, et al. The Landscape of Severe Combined Immunodeficiency Newborn Screening in the United States in 2020: A Review of Screening Methodologies and Targets, Communication Pathways, and Long-Term Follow-Up Practices. Front Immunol 2020; 11: 577853. doi:10.3389/fimmu.2020.577853

[194] Mütze U, Garbade SF, Gramer G, et al. Long-term Outcomes of Individuals With Metabolic Diseases Identified Through Newborn Screening. Pediatrics 2020; 146: e20200444. doi:10.1542/peds.2020-0444

[195] Zuckerman AE, Badawi D, Brosco JP, Brower A, Finitzo T, Flannery D, Green N, Greene C, Hassell K, Leslie ND, Mann S, Mistry KB, Monaco J, Ream MA, Schneider J, Sciortino S, Singh R, Sontag M, Thomas J. The Role of Quality Measures to Promote Long-Term Follow-up of Children Identified by Newborn Screening Programs. Presented by the Follow-up and Treatment Workgroup to the Advisory Committee on Heritable Disorders in Newborns and Children on February 8, 2018 (2018). Im Internet: https://www.hrsa.gov/sites/default/files/hrsa/advisory-committees/heritable-disorders/reports-recommendations/reports/role-of-quality-measures-in-nbs-sept2018-508c.pdf; Stand: 12.10.2021

[196] Wright EL, van Hove JLK, Thomas J. Mountain States Genetics Regional Collaborative Center's Metabolic Newborn Screening Long-term Follow-up Study: a collaborative multi-site approach to newborn screening outcomes research. Genet Med 2010; 12: S228-41. doi:10.1097/GIM.0b013e3181fe5d50

[197] Kölker S. The Value of European Registries and Networks for Changing Practice and Informing Guidelines [Abstract]. Int J Neonatal Screen 2021; 7: 7. doi:10.3390/ijns7040071

[198] Ministry of Health New Zealand. Newborn Metabolic Screening Programme monitoring indicators. Im Internet: https://www.nsu.govt.nz/system/files/page/newborn-metabolic-screening-programme-monitoring-indicators-feb18.pdf; Stand: 25.10.2021

[199] National Screening Unit New Zealand. Procedures, guidelines and reports. Im Internet: https://www.nsu.govt.nz/health-professionals/newborn-metabolic-screening-programme/procedures-guidelines-and-reports; Stand: 14.12.2021

[200] TNO - Nederlandse Organisatie voor toegepast-natuurwetenschappelijk onderzoek (Niederländische Organisation für angewandte wissenschaftliche Forschung). The newborn blood spot screening in the Netherlands, monitor 2019 (2021). Im Internet: https://www.pns.nl/documenten/newborn-blood-spot-screening-monitor-2019; Stand: 01.12.2021

[201] TNO - Nederlandse Organisatie voor toegepast-natuurwetenschappelijk onderzoek (Niederländische Organisation für angewandte wissenschaftliche Forschung). The Newborn Blood Spot Screening Monitor 2020. Im Internet: https://www.pns.nl/sites/default/files/2022-01/The%20Newborn%20Blood%20Spot%20Screening%20Monitor%202020.pdf; Stand: 11.05.2022

[202] RIVM (Staatsinstitut für öffentliche Gesundheit und Umwelt, Niederlande). Indicatorenset Neonatale Hielprikscreening (Indikatorensatz Neugeborenen-Fersenstichscreening) (2020); Stand: 01.12.2021

[203] Newborn Screening Ontario. Report Cards. The Newborn Screening Ontario Submitter Quality Indicator Report. Im Internet: https://www.newbornscreening.on.ca/en/health-care-providers/submitters/report-cards; Stand: 12.02.2022

[204] Newborn Screening Ontario. Annual Reports. Im Internet: https://www.newbornscreening.on.ca/en/about-nso/annual-reports; Stand: 12.02.2022

[205] Public Health England. Newborn blood spot screening standards (12.03.2021). Im Internet: https://www.gov.uk/government/publications/standards-for-nhs-newborn-blood-spot-screening; Stand: 11.03.2022

[206] Public Health England. Newborn blood spot screening: data collection and performance analysis reports (16.03.2021). Im Internet: https://www.gov.uk/government/publications/newborn-blood-spot-screening-data-collection-and-performance-analysis-report; Stand: 11.02.2022

[207] Registret för Medfödda Metabola Sjukdomar RMMS (Register für angeborene Stoffwechselkrankheiten, Schweden). Om RMMS (Über RMMS) (15.11.2021). Im Internet: https://rmms.se/om-rmms/; Stand: 15.11.2021

[208] Lajic S, Karlsson L, Zetterström RH, et al. The Success of a Screening Program Is Largely Dependent on Close Collaboration between the Laboratory and the Clinical Follow-Up of the Patients. Int J Neonatal Screen 2020; 6: 68. doi:10.3390/ijns6030068

[209] Berry SA, Jurek AM, Anderson C, et al. The inborn errors of metabolism information system: A project of the Region 4 Genetics Collaborative Priority 2 Workgroup. Genet Med 2010; 12: S215-9. doi:10.1097/GIM.0b013e3181fe5d23

[210] Berry SA, Leslie ND, Edick MJ, et al. Inborn Errors of Metabolism Collaborative: large-scale collection of data on long-term follow-up for newborn-screened conditions. Genet Med 2016; 18: 1276–1281. doi:10.1038/gim.2016.57

[211] Hinton CF, Mai CT, Nabukera SK, et al. Developing a public health-tracking system for follow-up of newborn screening metabolic conditions: a four-state pilot project structure and initial findings. Genet Med 2014; 16: 484–490. doi:10.1038/gim.2013.177

[212] Brower A, Chan K, Hartnett M, et al. The Longitudinal Pediatric Data Resource: Facilitating Longitudinal Collection of Health Information to Inform Clinical Care and Guide Newborn Screening Efforts. Int J Neonatal Screen 2021; 7. doi:10.3390/ijns7030037

[213] Opladen T, Gleich F, Kozich V, et al. U-IMD: the first Unified European registry for inherited metabolic diseases. Orphanet J Rare Dis 2021; 16: 95. doi:10.1186/s13023-021-01726-3

[214] U-IMD. Unified Registry for Inherited Metabolic Disorders. Im Internet: https://u-imd.org/; Stand: 13.01.2022

[215] Singh RH, Brown SJ, Hale PM, et al. Using Informatics to Build a Digital Health Footprint of Patients Living With Inherited Metabolic Disorders Identified by Newborn Screening. J Public Health Manag Pract 2020; 28: E340-E344. doi:10.1097/PHH.0000000000001250

[216] RIVM (Staatsinstitut für öffentliche Gesundheit und Umwelt, Niederlande). Privacy en registratie van gegevens | Draaiboek Hielprikscreening (Datenschutz und Registrierung von Daten | Skript Fersenstich-Screening). Im Internet: https://draaiboekhielprikscreening.rivm.nl/proces/privacy-en-registratie-van-gegevens; Stand: 18.11.2021

[217] Instytut Matki i Dziecka (Institut für Mutter und Kind, Polen). Badania przesiewowe noworodków (Neugeborenen-Screening-Tests) (14.11.2021). Im Internet: https://przesiew.imid.med.pl/badaniaprzesiewowe.html; Stand: 18.01.2022

[218] Statens Serum Institut (Staatliches Serum-Institut Dänemark). Screening af nyfødte. For sundhedspersonale (Neugeborenenscreening. Für Gesundheitspersonal) (15.07.2021). Im Internet: https://nyfoedte.ssi.dk/for-sundhedspersonale; Stand: 10.12.2021

[219] Repič Lampret B, Remec ŽI, DroleTorkar A, et al. Expanded Newborn Screening Program in Slovenia using Tandem Mass Spectrometry and Confirmatory Next Generation Sequencing Genetic Testing. Zdr Varst 2020; 59: 256–263. doi:10.2478/sjph-2020-0032

[220] Egészségügyi Minisztérium (Gesundheitsministerium Ungarn). Információk ÚJSZÜLÖTTKORI ANYAGCSERESZŰRÉS (Information SCREENING DES NEUGEBORENEN STOFFWECHSELS). Im Internet: https://anyagcsere.sed.hu/; Stand: 23.08.2021

[221] California Department of Public Health. Online Specimen Tracking (OST). Im Internet: https://www.cdph.ca.gov/Programs/CFH/DGDS/Pages/nbs/ost.aspx; Stand: 18.02.2022

[222] Newborn Screening Ontario. Track-Kit - Sign In - Track-Kit. Im Internet: https://track.newbornscreening.on.ca/; Stand: 18.11.2021

[223] Medizinische Universität Wien. Abfrage "Screeningkärtchen im Labor eingelangt?". Im Internet: https://kinderklinik.meduniwien.ac.at/index.php?id=13366; Stand: 18.01.2022

[224] Sörensen L, Zetterström R. IT Infrastructure for Screening, Diagnosis and Long-Term Follow-up for Newborn Screening in Sweden. Int J Neonatal Screen 2020; 6: 18. doi:10.3390/ijns6010012

[225] Newborn Screening Ontario. Promoting the Use of OLIS to Access Newborn Screening Results. Im Internet: https://www.newbornscreening.on.ca/sites/default/files/bulletins/2021-6_1.pdf; Stand: 18.11.2021

[226] eHealth Ontario. Ontario Laboratories Information System (OLIS) Standard | eHealth Ontario | It's Working For You. Im Internet: https://ehealthontario.on.ca/en/standards/ontario-laboratories-information-system-standard; Stand: 18.03.2022

[227] Association of Public Health Laboratories (APHL). Building Blocks: Newborn Screening Health IT Implementation Guide and Toolkit (07.12.2021). Im Internet: https://www.newsteps.org/resources/toolkits/building-blocks-newborn-screening-health-it-implementation-guide-and-toolkit; Stand: 07.12.2021

[228] Jones D, Shao J, Wallis H, et al. Towards a Newborn Screening Common Data Model: The Utah Newborn Screening Data Model. Int J Neonatal Screen 2021; 7. doi:10.3390/ijns7040070

[229] Watkins M, Au A, Vuong T, et al. ResultsMyWay: combining Fast Healthcare Interoperability Resources (FHIR), Clinical Quality Language (CQL), and informational resources to create a newborn screening application. AMIA Joint Summits on Translational Science proceedings 2021; 2021: 615–623

[230] Miller FA, Cressman C, Hayeems R. Governing population screening in an age of expansion: The case of newborn screening. Can J Public Health 2015; 106: e244-8. doi:10.17269/cjph.106.4897

[231] Ficicioglu C. New tools and approaches to newborn screening: ready to open Pandora's box? Cold Spring Harb Mol Case Stud 2017; 3: a001842. doi:10.1101/mcs.a001842

[232] Andermann A. Presentation: Revisiting Wilson and Jungner in the genomic age: a review of screening criteria over the past 40 years. VHPB Budapest; 2010

[233] Gemeinsamer Bundesausschuss (G-BA). Beschlussbegründung über eine Änderung der Kinder-Richtlinien zur Einführung des erweiterten Neugeborenen-Screenings vom 21. Dezember 2004; Stand: 18.04.2022

[234] Liebl B, Nennstiel-Ratzel U, Roscher A, et al. Data required for the evaluation of newborn screening programmes. Eur J Pediatr 2003; 162 Suppl 1: S57-61. doi:10.1007/s00431-003-1354-0

[235] Schnakenburg K von, Zoubek A. Neue Screening-Richtlinien. Monatsschrift Kinderheilkunde 2002; 150: 1424–1440. doi:10.1007/s00112-002-0611-z

[236] La Marca G. The Newborn Screening Program in Italy: Comparison with Europe and other Countries. Rev Esp Salud Publica 2021; 95

[237] Ministero della Salute (Gesundheitsministerium Italien). Screening neonatali (Neugeborenen-Screening) (19.11.2021). Im Internet: https://www.salute.gov.it/portale/saluteBambinoAdolescente/dettaglioContenutiSaluteBambinoAdolescente.jsp?lingua=italiano&id=1920&area=saluteBambino&menu=nascita; Stand: 25.01.2022

[238] Munck A, Gauthereau V, Czernichow P. Organisation du dépistage néonatal en France. M/S 2021; 37: 457–460. doi:10.1051/medsci/2021063

[239] Downing M, Pollitt R. Newborn bloodspot screening in the UK--past, present and future. Ann Clin Biochem 2008; 45: 11–17. doi:10.1258/acb.2007.007127

[240] GOV.UK. UK National Screening Committee. Im Internet: https://www.gov.uk/government/organisations/uk-national-screening-committee; Stand: 18.11.2021

[241] Australian Government Department of Health. Newborn Bloodspot Screening – National Policy Framework. Im Internet: https://www.health.gov.au/resources/publications/newborn-bloodspot-screening-national-policy-framework; Stand: 24.08.2021

[242] National Screening Unit New Zealand. Newborn Metabolic Screening Programme. Im Internet: https://www.nsu.govt.nz/health-professionals/newborn-metabolic-screening-programme; Stand: 14.11.2021

[243] Heather N, Knoll D, Shore K, et al. Application of a Protocol for Structured Follow-Up and Texting of Inadequate or Bordeline-Positive Screen Results [Abstract]. Int J Neonatal Screen 2021; 7: 12. doi:10.3390/ijns7040071

[244] McRoberts C. Optimizing Pre-Analytical Processes to Ensure the Reliability of Newborn Screening Results [Abstract]. Int J Neonatal Screen 2018; 4: 11–12. doi:10.3390/ijns4030028

[245] RIVM (Staatsinstitut für öffentliche Gesundheit und Umwelt, Niederlande). Hielprik (Fersenstich) (30.11.2021). Im Internet: https://www.pns.nl/hielprik; Stand: 30.11.2021

[246] van Dijk T, Kater A, Jansen M, et al. Expanding Neonatal Bloodspot Screening: A Multi-Stakeholder Perspective. Front Pediatr 2021; 9: 706394. doi:10.3389/fped.2021.706394

[247] Ministerie van Volksgezondheid, Welzijn en Sport (Gesundheitsministerium Niederlande). Wet op het bevolkingsonderzoek (WBO, Bevölkerungsscreeninggesetz der Niederlande) (01.07.2021). Im Internet: https://wetten.overheid.nl/BWBR0005699/2021-07-01; Stand: 22.03.2022

[248] RIVM (Staatsinstitut für öffentliche Gesundheit und Umwelt, Niederlande). Welke bevolkingsonderzoeken zijn er? (Welche Bevölkerungsscreenings gibt es?). Im Internet: https://www.rivm.nl/bevolkingsonderzoeken-en-screeningen/bevolkingsonderzoek-en-u/welke-bevolkingsonderzoeken-zijn-er; Stand: 18.11.2021

[249] RIVM (Staatsinstitut für öffentliche Gesundheit und Umwelt, Niederlande). Policy framework for Prenatal and Neonatal Screening (2018). Online: https://www.rivm.nl/bibliotheek/rapporten/2018-0043.pdf; accessed: März 31, 2022

[250] RIVM (Staatsinstitut für öffentliche Gesundheit und Umwelt, Niederlande). Organisatie Prenatale en neonatale screeningen (Organisation prä- und neonatale Screenings). Im Internet: https://www.pns.nl/hielprik/professionals/organisatie; Stand: 18.01.2022

[251] RIVM (Staatsinstitut für öffentliche Gesundheit und Umwelt, Niederlande). RIVM-DVP. Im Internet: https://draaiboekhielprikscreening.rivm.nl/rol-ketenpartners/rivm-dvp; Stand: 18.01.2022
